# Dimerization-Dependent Trans-Domain Coupling Enables Intermediate Transfer in Fungal Haloacid Dehalogenase-Like Terpene Cyclases

**DOI:** 10.1021/jacs.6c04151

**Published:** 2026-05-15

**Authors:** Tzu-Ho Chen, Kai-Fa Huang, Ting-Hung Chou, Cheng-Chung Tseng, Rou-Jie Huang, Tzu-Ping Ko, Suh-Yuen Liang, Rong-Jie Chein, Hsiao-Ching Lin

**Affiliations:** † 38017 Institute of Biological Chemistry, Academia Sinica, Taipei 115, Taiwan R.O.C; ‡ Biomedical Translation Research Center (BioTReC), Academia Sinica, Taipei 11529, Taiwan R.O.C; § School of Pharmacy, College of Medicine, 33561 National Taiwan University, Taipei 100, Taiwan R.O.C; ∥ Department of Chemistry, National Taiwan University, Taipei 106, Taiwan R.O.C; ⊥ Institute of Chemistry, Academia Sinica, Taipei 11529, Taiwan R.O.C

## Abstract

Drimane-type sesquiterpenes (DTSs) are a widely distributed terpenoid family with diverse and potent bioactivities. Although DTS synthases occur in plants, bacteria, and fungi, they share little sequence identity across kingdoms, obscuring the mechanistic principles that govern drimane scaffold formation and product phosphorylation states. Haloacid dehalogenase (HAD)-like terpene cyclases (TCs), TC domains fused to HAD-like phosphatases, are especially intriguing because they couple cyclization with dephosphorylation, yet how these enzymes control scaffold outcomes and phosphorylation states across kingdoms remains unresolved. Here, we identify the fungal enzyme AacA as a bifunctional albicanoyl monophosphate synthase that catalyzes class II cyclization of farnesyl pyrophosphate, followed by Mg^2+^-dependent dephosphorylation. We further determine the X-ray crystal structures of the fungal drim-8-ene-11-yl pyrophosphate synthase AstC, representing the first structures of fungal HAD-like terpene cyclases. These structures capture substrate- and product-mimic states and reveal a head-to-tail homodimer in which the partner HAD-like domain caps the TC active site and positions the pyrophosphate at the intersubunit interface, consistent with *trans*-domain intermediate transfer. In addition, phosphate-release kinetics support cross-monomer TC-to-HAD coupling in dimeric AacA. Structure-guided mutagenesis and assays with farnesyl mono- and thiopyrophosphate analogues further define the determinants of product selectivity and the distinct dephosphorylation capacities of these enzymes. These findings expand fungal DTS enzymology and guide TC engineering.

## Graphical abstract

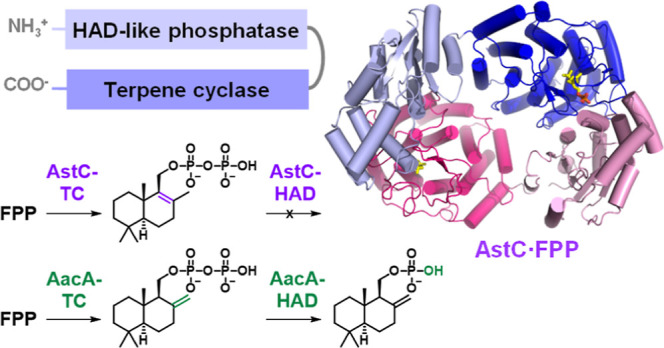

## Introduction

Terpenoids are one of the largest and most structurally diverse classes of natural products.^1^ They are produced across all domains of life and are fundamental to key biological functions, including plant defense, microbial communication, and signaling in animals. Terpenoids are widely used as fragrances, flavoring agents, nutraceuticals, pesticides, and pharmaceuticals. Growing demand for complex terpenoids, together with sustainability concerns over extraction from natural sources, has stimulated intense interest in understanding and engineering the enzymes that build their carbon skeletons.

All terpenoids derive from the universal C_5_ precursors dimethylallyl pyrophosphate and isopentenyl pyrophosphate. Sequential head-to-tail condensations catalyzed by prenyltransferases generate linear prenyl pyrophosphates, geranyl (C_10_), farnesyl (C_15_), and geranylgeranyl (C_20_) pyrophosphates.^2^ These intermediates are then converted into diverse hydrocarbon frameworks by terpene cyclases (TCs).^3^ TCs catalyze carbocationic cascades that produce multicyclic scaffolds with multiple stereocenters from a single acyclic substrate. Mechanistically, TCs are grouped into class I and class II, which initiate cyclization through pyrophosphate ionization and double-bond protonation, respectively, using conserved DDxxD/NSE–DTE (class I) and DxDD (class II) motifs embedded in characteristic α, β, and γ domain architectures.^4^

Drimane-type sesquiterpenes (DTSs) constitute a prominent terpenoid family defined by a bicyclic C_15_ “drimane” scaffold (Figure 1A).^5^ DTSs are produced by plants, fungi, bacteria, and marine organisms and display broad bioactivities, including antifeedant, insecticidal, antibacterial, antifungal, antiviral, and antiproliferative effects.^6^ Some DTSs contribute to the pungent taste of culinary herbs such as water pepper, whereas others, exemplified by (−)-antrocin from the medicinal mushroom *Antrodia cinnamomea*, are key bioactive metabolites with promising anticancer properties.^7^

**Figure 1:**
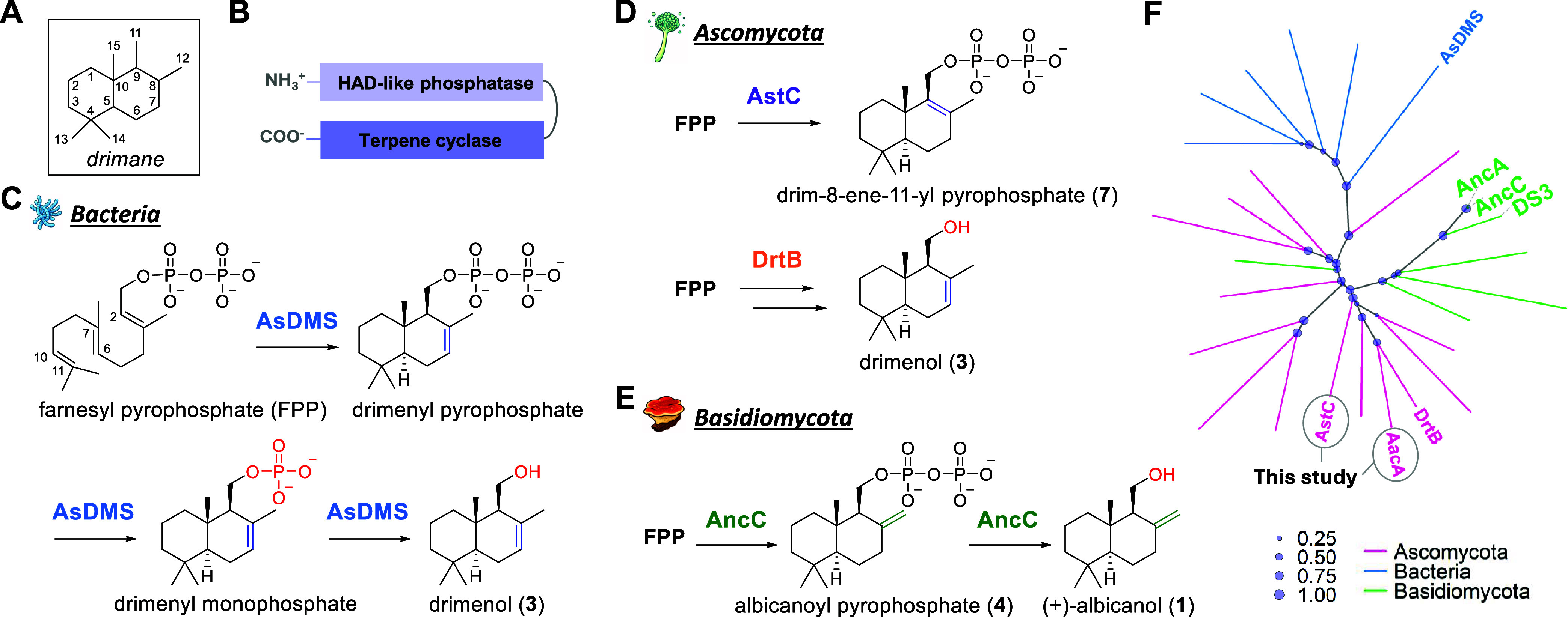
Drimane-type sesquiterpenes and haloacid dehalogenase (HAD)-like terpene cyclases (TCs). (A) Drimane scaffold. (B) HAD-like TC domain architecture. (C–E) Representative HAD-like TCs from bacteria (C), Ascomycota (D), and Basidiomycota (E) and their functions. (F) Phylogeny of bacterial and fungal HAD-like TCs; simplified tree (27 homologues) generated as described in the Supporting Information Methods.

Drimane-type sesquiterpene synthases occur in plants, bacteria, and fungi but share little amino acid sequence identity.^5b^ Plant drimenol synthases such as VoTPS3 ^8^ and PhDS^9^ are canonical class I sesquiterpene cyclases that convert farnesyl pyrophosphate (FPP) into drimenol. By contrast, bacterial drimenyl pyrophosphate synthases (SsDMS/ScDMS) from *Streptomyces* are class II enzymes with βγ-didomain architecture and a catalytic DxDD motif at the β–γ interface.^10^ A recently emerged noncanonical family of haloacid dehalogenase (HAD)-like terpene cyclases (TCs) features a TC domain is fused to a HAD-like phosphatase domain (HAD–TC) (Figure 1B–F).

Representative members include the bacterial drimenol synthase AsDMS^11^ and the basidiomycete (+)-albicanol synthase AncC,^7^ and the ascomycete enzymes AstC^12^ and DrtB,^13^ which produce drimane pyrophosphate or drimenol intermediates (Figure 1). Recent structural work on AsDMS revealed a dimeric HAD–TCβ architecture that supports metal-dependent dephosphorylation and an electrostatic environment that promotes intermediate transfer through bulk solution.^14^ Despite these advances, it remains unclear how HAD-like TCs across kingdoms, with minimal amino acid sequence identity, converge on drimane scaffolds yet exhibit distinct product selectivities. In particular, the molecular logic by which the fungal TC domain controls scaffold formation, double-bond placement, and deprotonation, and how the HAD-like domain recognizes and dephosphorylates cyclized intermediates, is still unclear.

Here we discovered and characterized the ascomycete enzyme AacA (albicanoyl monophosphate synthase) and compared it with AstC (drim-8-ene-11-yl pyrophosphate synthase). X-ray structures, mutagenesis, and biochemistry define the TC acid–base pair and aromatic determinants of cyclization/deprotonation, while structures and kinetics indicate head-to-tail dimers enable *trans*-domain intermediate transfer. These insights provide a mechanistic framework to understand and engineer fungal HAD-like TCs for biocatalysis.

## Results and Discussion

### Genome-Mined AacA is a Bifunctional Albicanoyl Monophosphate Synthase from *Aspergillus aculeatus*

Through genome mining of ascomycete fungal databases, we identified a HAD-like TC from *A. aculeatus*, designated AacA (Figures 1F and 2A). AacA shares 39.6% and 41.6% sequence identity with the previously characterized AstC^12^ and AncC,^7^ respectively. To assess in vivo function, an intron-free *aacA* was expressed in *Saccharomyces cerevisiae* BJ5464-NpgA.^15^ Gas chromatography–electron ionization mass spectrometry (GC–EI/MS) of yeast extracts detected (+)-albicanol (**1**), showing AacA cyclizes FPP to a drimane scaffold in vivo. Under the same conditions, AstC and DrtB produced drim-8-ene-11-ol (**2**) and drimenol (**3**), respectively, confirmed by EI/MS comparisons (Figure S2).^7,12,13^

**Figure 2:**
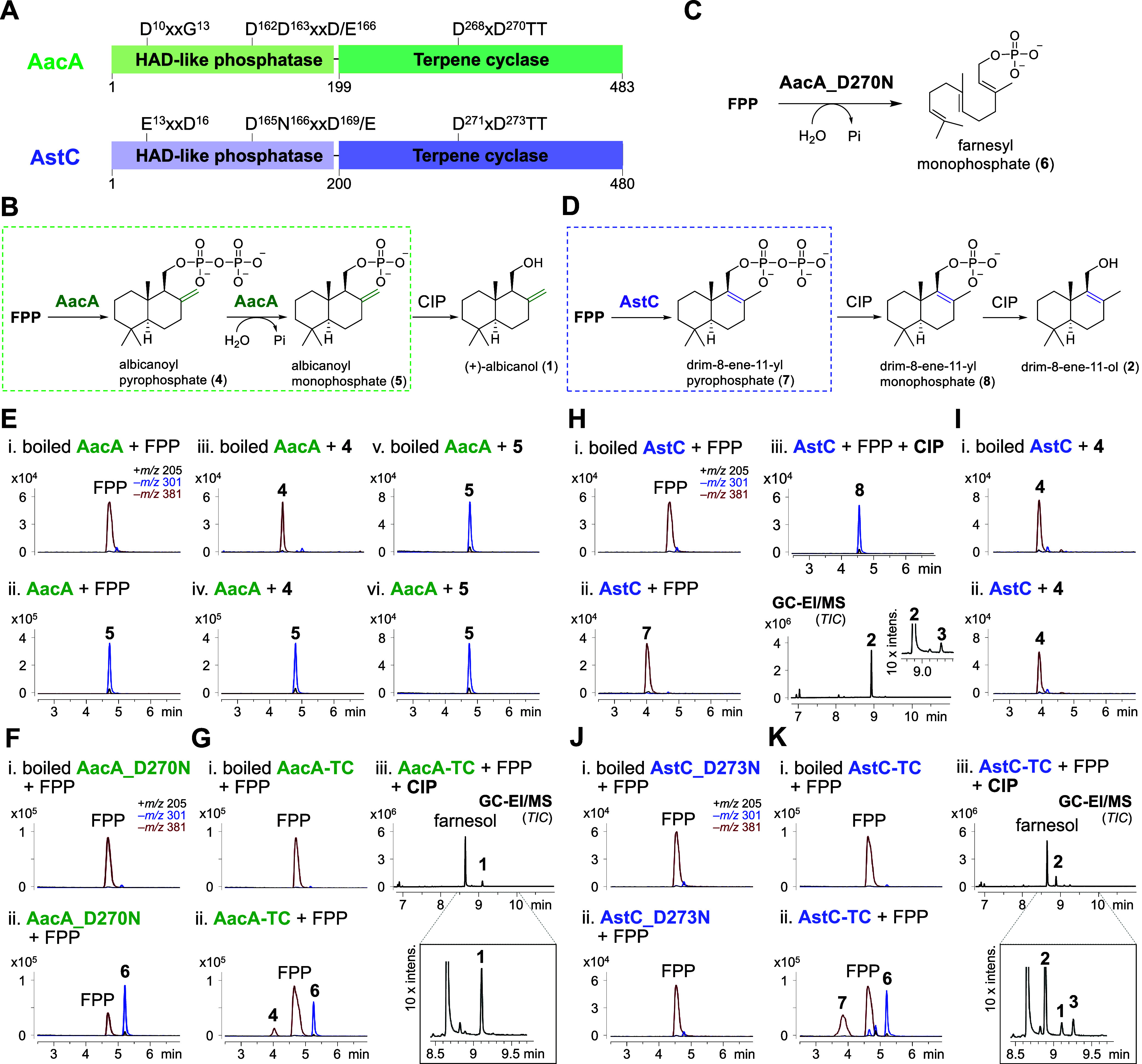
In vitro functional verification of AacA, AstC, and variants. (A) Domain architectures of AacA and AstC. Enzymatic functions of (B) AacA (C) AacA_D270N, and (D) AstC. (E–K) LC–MS and GC–EI/MS chromatograms of in vitro assays: (E) AacA with FPP, **4**, or **5**; (F) AacA_D270N with FPP; (G) AacA-TC with FPP; (H) AstC with FPP; (I) AstC with **4**; (J) AstC_D273N with FPP; (K) AstC-TC with FPP. The selected-ion monitoring MS traces are shown, with each *m*/*z* value indicated by a different color. For reactions with calf intestinal alkaline phosphatase (CIP), CIP was added 5 min after initiation and incubated for an additional 30 min.

To verify AacA activity in vitro, N-His-tagged recombinant AacA was purified from *Escherichia coli* (Figure S3). Incubation with FPP and MgCl_2_ produced albicanoyl monophosphate (**5**), detected by liquid chromatography–mass spectrometry (LC–MS), whereas **1** was not observed by LC–MS or GC–EI/MS (Figures 2B,E and S4). Compound **5** matched a synthetic standard (Supporting Information) by retention time and mass spectrum (Figure S31). Using synthetic albicanoyl pyrophosphate (**4**)^7^ as substrate, AacA efficiently converted **4** to **5**, confirming phosphatase activity, but **5** was not converted to **1** (Figure 2E). Thus, AacA is bifunctional, catalyzing class II cyclization (FPP to **4**) followed by dephosphorylation (**4** to **5**).

### Dissecting the Catalytic Roles of TC and HAD-Like Domains in AacA

To dissect AacA catalysis, we expressed and purified the isolated TC domain AacA-TC (residues 196–466) and a site-directed mutant AacA_D270N in the conserved D^268^xD^270^T motif (Figure S5). In vitro, AacA_D270N incubated with FPP and MgCl_2_ produced farnesyl monophosphate (**6**) but not albicanoyl monophosphate (**5**), showing Asp270 is essential for cyclization (Figures 2C,F, and S6). When supplied with **4**, the mutant still yielded **5** (Figure S7), whereas **5** was not converted to **1** (Figure S8). EDTA suppresses phosphatase activity but not cyclization (Figure S9). Thus, the HAD-like domain acts as a divalent cation–dependent phosphatase, cleaving one phosphate from FPP or **4**. Incubating the isolated AacA-TC domain with FPP produced **4**, and calf intestinal alkaline phosphatase (CIP) treatment enabled GC–MS detection of **1** (Figure 2G). Minor **6** formation likely arose from trace copurifying *E. coli* proteins, including phosphatase-related proteins^16,17^ identified by proteomic analysis (Tables S4 and S5). The above results demonstrate that AacA-TC first cyclizes FPP to **4**, and then the HAD-like domain cleaves one phosphate group to yield **5**.

### AstC Lacks Phosphatase Activity but Its HAD-Like Domain Coregulates Cyclase Product Selectivity

Because AacA and the previously characterized AstC differ functionally, we examined AstC for domain-specific functions. Full-length AstC was reconstituted and purified from *E. coli* (Figure S10). In vitro assays showed that AstC cyclizes FPP to drim-8-ene-11-yl pyrophosphate (**7**), but does not dephosphorylate **7** to the corresponding monophosphate (**8**) or alcohol **2** (Figures 2D,H and S11), consistent with the previous report.^12^ Likewise, incubating AstC with **4** or **5** did not produce any dephosphorylated products (Figures 2I and S12), indicating that AstC lacks phosphatase activity.

To probe the HAD-like region, we generated AstC_D273N in the conserved D^271^xD^273^T motif. This mutant failed to convert FPP to any cyclized product, showing Asp273 is essential for cyclization (Figure 2J). AstC_D273N also did not dephosphorylate **4** (Figure S13), supporting that AstC’s HAD-like domain is inactive in phosphate hydrolysis. With the isolated AstC-TC domain, LC–MS showed accumulation of **7** with **6** and minor monophosphate species (same *m*/*z* as **6**), likely arising from nonspecific hydrolysis by trace copurifying phosphatase-related proteins from *E. coli* in the enzyme preparation^16,17^ (Figure 2K; Tables S4 and S5). After CIP treatment, GC–EI/MS detected **2** as the major product with minor **1** and **3**. In contrast, full-length AstC plus CIP yielded mainly **2** with trace **3** (Figure 2H, iii). Thus, the nonhydrolytic HAD-like domain coregulates cyclization together with the TC domain in AstC.

### Crystal Structures of AstC Reveal a Two-Domain HAD-like Terpene Cyclase with a Noncanonical Phosphatase Site

Given the functional diversity of HAD-like TCs, structural information are needed to understand carbocation handling in the TC domain and whether the HAD-like region supports dephosphorylation. To define the catalytic basis of fungal HAD-like TCs, we solved X-ray structures of AstC^•^FPP, the *apo* AstC-TC, and AstC-TC^•^ **5**. These structures provide mechanistic snapshots of TC-domain cyclization and show how AstC’s HAD-like domain, despite lacking phosphatase activity, shapes the fate of downstream phosphate-containing intermediates.

The AstC^•^FPP complex (AstC Ile^4^–Asn^475^_D273N bound to FPP) crystallized in a primitive orthorhombic lattice and was solved at 2.95 Å resolution by molecular replacement (Table S1). Two AstC protomers occupy the asymmetric unit and form an intertwined, antiparallel dimer (Figure 3A). Electron density supported modeling of Pro^6^–Gln^473^ (chain A) and Pro^6^–Ala^474^ (chain B), with FPP bound in both chains (Figure 3B). Dimerization is supported by PISA (Protein Interfaces, Surfaces and Assemblies) analysis (interface ∼ 2260 Å^2^)^18^ and size-exclusion chromatography with multiangle light scattering (SEC–MALS), which gave an apparent molecular weight of 113.2 kDa (Figure S14). Each protomer contains two well-separated domains: an N-terminal HAD-like Rossmann α/β fold (Pro^6^–Tyr^199^)^19^ and a C-terminal compact 12-helix (α_6_–α_6_ barrel) bundle (Asp^200^–Gln^473^) resembling the class II terpene cyclase β-domain (Figure 3C,D).^20^

**Figure 3:**
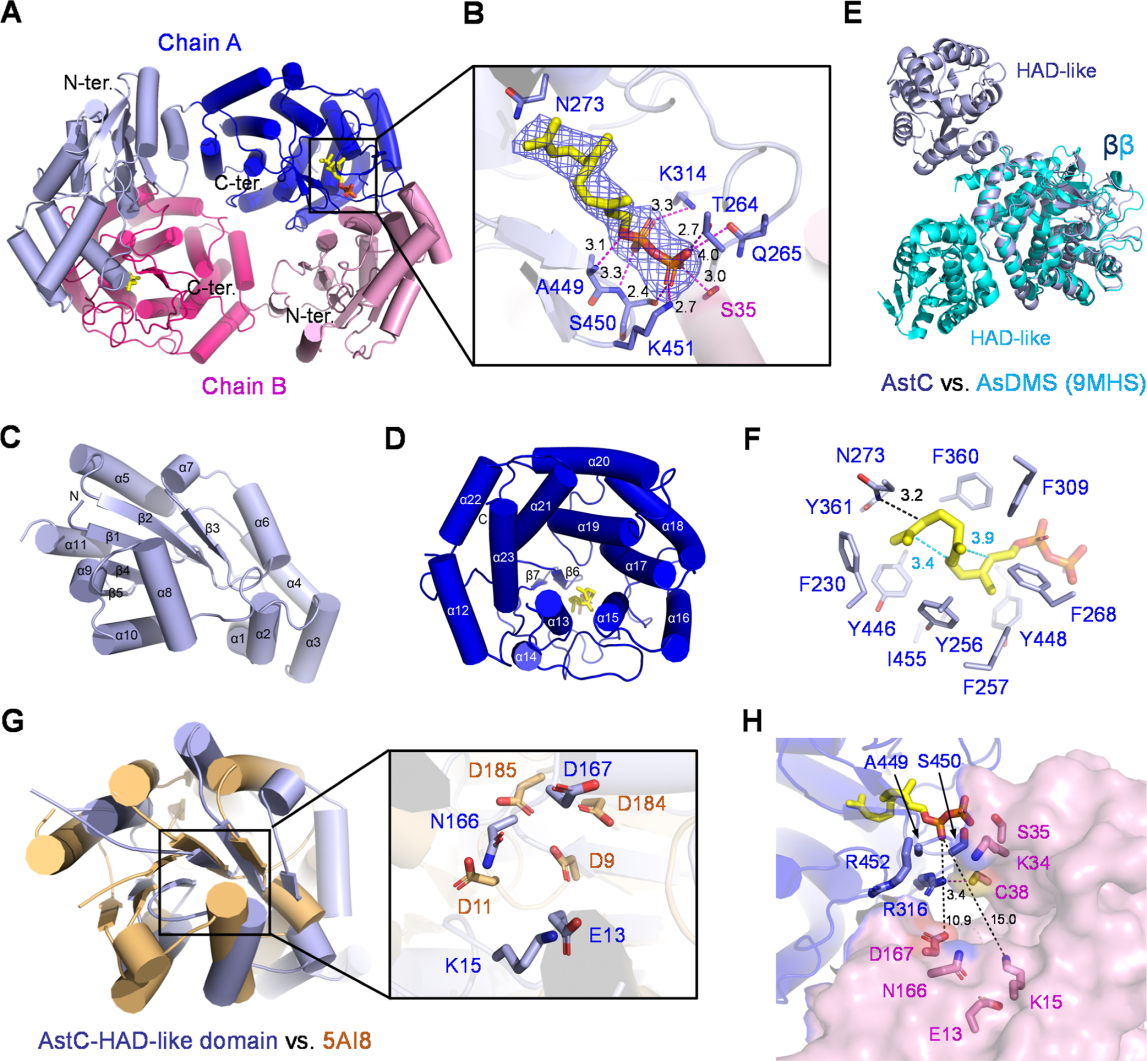
Structures of AstC in complex with FPP. (A) Head-to-tail homodimer architecture of the AstC^•^FPP quaternary complex (Protein Data Bank identification: 22TZ). (B) Electron density map of the AstC-bound FPP (*F*_0_-*F*_c_ omit map contoured at 3.0σ). (C) Rossmannoid fold of the AstC HAD-like domain. (D) α_6_–α_6_ barrel fold of the AstC TC domain. (E) Superposition of AstC and AsDMS monomers. (F) Active-site cavity showing key aromatic residues and N273 surrounding FPP. (G) Superposition of the AstC HAD-like domain with human epoxide hydrolase (PDB: 5AI8). (H) Dimer interface between the Chain A TC domain (blue) and Chain B HAD-like domain (magenta) in the AstC^•^FPP complex.

A Dali^21^-search using full-length AstC identified a closely related two-domain architecture in the recently reported *Streptomyces*drimenol synthase AsDMS (PDB 9MHS; Z-score 34.1; RMSD 6.7 Å over 286 Cα; 26% sequence identity) (Figure 3E).^14^ Despite this overall similarity, the relative orientation of the HAD-like and TC domains differs substantially between AstC and AsDMS (Figure S16), suggesting that interdomain packing is a major variable within the family.

When analyzed separately, the C-terminal TC domain of AstC also aligns with established class II cyclase β-domains, including squalene–hopene cyclase (PDB 1H3B; Z-score 21.3; RMSD 3.6 Å over 261 Cα; 13% identity),^20^ MstE (PDB 6SBC; Z-score 21.3; RMSD 3.1 Å over 251 Cα; 13% identity),^22^ and SsDMS (PDB 7XRA; Z-score 20.7; RMSD 3.4 Å over 257 Cα; 13% identity)^10^ (Figure S15). Thus, AstC preserves the canonical class II cyclase fold, yet the local architecture surrounding bound FPP differs markedly from these enzymes, consistent with specialized substrate positioning and intermediate control.

By contrast, the N-terminal HAD-like domain resembles the HAD-like region of human soluble epoxide hydrolase (PDB 5AI8; Z-score 20.2; RMSD 2.4 Å over 185 Cα; 18% identity) (Figure 3G).^19^ In canonical HAD-like phosphatases, three conserved Asp residues coordinate an essential Mg^2+^ cofactor for phosphate chemistry (Figures 3G and S19). In AstC, the corresponding sites are noncanonical (e.g., Glu13, Asp166, Asp167), and no divalent metal is observed in the putative active site. This structural divergence provides a rationale for AstC’s lack of phosphatase activity in vitro.

### Dimer-Mediated Intermediate Transfer between the TC and HAD-like Domains

In the AstC^•^FPP structure, FPP binds at the dimer interface between the C-terminal TC domain of chain A and the N-terminal HAD-like domain of chain B (Figure 3A). The C_15_ farnesyl chain inserts into a pronounced cavity, forming an elongated substrate tunnel (16 Å deep, 12 Å wide), whereas the pyrophosphate points toward the channel opening and lies near the partner HAD-like domain, with interdomain distances of 10.9–15.0 Å (Figure 3H). The tunnel constricts to 3.4 Å at C38 and R316, which interact via a thiolate hydrogen bond. An R316–D169 salt bridge further orients the R316 guanidinium toward C38.

This head-to-tail, intertwined dimer provides a structural rationale for *trans*-domain intermediate transfer: following TC-domain cyclization, a pyrophosphate-bearing intermediate could be directly transferred to the opposing HAD-like domain for phosphate processing, even though AstC itself lacks hydrolytic activity. Consistent with a similar architecture in AacA, an AlphaFold2 ^23^ model predicts stabilizing TC/HAD contacts, including an R356–E166 salt bridge and an R313–S36 hydrogen bond (Figure S17). SEC–MALS further supports dimerization of AacA, yielding an apparent molecular weight of 114.3 kDa (Figure S18), comparable to AstC.

Together, these data reveal that cross-monomer coupling of TC and HAD-like domains within a head-to-tail dimer is a shared feature of ascomycete HAD-like terpene cyclases, enabling efficient cyclization and coordinated handling of downstream phosphate-containing intermediates.

### FPP Recognition in the AstC TC Domain and Capping by the HAD-Like Domain

In each AstC subunit, FPP binds within the TC domain at the center of the 12-helix bundle and adopts an *E*,*E*-configuration (Figure 3D). Its pyrophosphate group extends toward the HAD-like domain of the opposing subunit (Figure 3B), consistent with an intersubunit arrangement that partially encloses the substrate. The pyrophosphate is stabilized by ionic interactions with K314 and K451, hydrogen bonding with T264, Q265, A449, and S450, and an additional hydrogen bond contributed by S35 from the neighboring HAD-like domain. This cross-subunit contact effectively caps the FPP pocket, limiting access to the active site.

Mutagenesis supported the functional importance of these interactions (Figures 4G and S20). Substituting K314 with arginine reduced formation of product **2** by <25%, whereas K314A nearly eliminated activity, indicating that K314 is critical for electrostatic anchoring of the α-phosphate. Mutations K451A and S35A also decreased product formation, consistent with roles in β-phosphate positioning and substrate alignment. In contrast to the polar headgroup, the farnesyl chain is buried in a hydrophobic cavity formed by F230, Y256, F257, F268, F309, F360, Y361, Y446, Y448, and I455 (Figure 3F). In the AstC_D273N variant, N273 lies 3.2 Å from the terminal olefin, placing it near the reactive end of the substrate.

**Figure 4:**
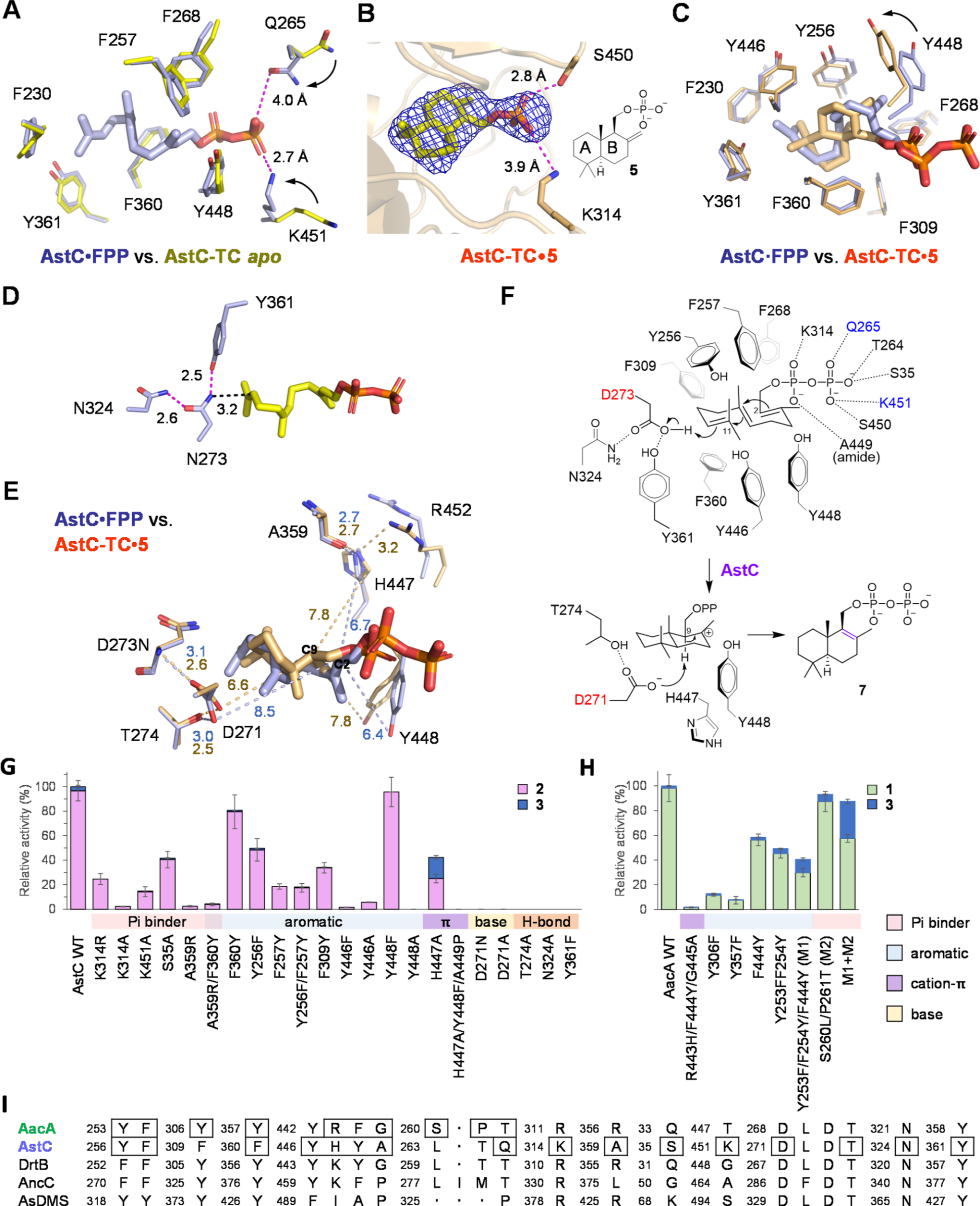
Structural basis and mechanism of AstC TC-domain cyclization and structure-guided mutagenesis. (A–E) Structural snapshots of AstC in complex with FPP (AstC^•^FPP), AstC-TC with substrate mimic **5** (AstC-TC^•^ **5**) (PDB: 22TY), and *apo* AstC-TC (PDB: 22UA). (A) Q265 and K451 as pyrophosphate sensors. (B) Electron density (Fo-Fc omit map contoured at 3.0σ) for bound **5**; S450 and K314 coordinate the α-phosphate. (C) Superposition of AstC^•^FPP and AstC-TC^•^ **5** reveals repositioning of aromatic residues during cyclization. (D) N324 and Y361 activate the Brønsted acid (D273) for protonation of the terminal C=C bond of FPP. (E) D271 as the putative catalytic base for deprotonation of the final carbocation intermediate. (F) Proposed TC-domain cyclization mechanism of AstC. (G,H) Relative activities of AstC (G) and AacA (H) mutants expressed in yeast. (I) Alignment of key cyclization residues from AacA, AstC, DrtB, AncC, and AsDMS. Boxed residues were targeted for mutagenesis.

### Ligand-Induced Active-Site Remodeling in AstC and an Aromatic Gatekeeper for Product Selectivity

To capture conformational changes linked to FPP binding and cyclization, we solved the *apo* structure of the isolated TC domain (AstC-TC, Asp^200^–Asn^475^), because full-length AstC could not be crystallized in the absence of FPP. Superposition with the FPP-bound structure revealed marked rearrangements of Q265 and K451 (Figure 4A). In the *apo* state, both side chains are flexible and oriented outward; upon FPP binding, they rotate inward to engage the β-phosphate through hydrogen bonding, consistent with a pyrophosphate-sensing “gate” similar to that described for SsDMS.^10^ In parallel, multiple aromatic residues lining the active site undergo subtle inward rotations, tightening packing around the farnesyl chain of bound FPP (Figure 4A).

We next determined a 2.85 Å structure of AstC-TC in complex with **5** (AstC-TC^•^5; Figure 4B). Compound **5**, introduced by soaking with synthetic **5** (Supporting Information), serves as a structural mimic of the cyclized intermediate **7**. Relative to the FPP-bound state, binding of **5** induces a pronounced inward rotation of Y448 and modest compensatory shifts of nearby aromatics (Figure 4C), reshaping the pocket to accommodate a bicyclic ligand rather than elongated FPP. The monophosphate is stabilized primarily by an ionic interaction with K314 and a hydrogen bond with S450 (Figure 4B). The bicyclic scaffold is positioned by an aromatic network that frames the A-ring (F230, F360, Y361, Y446) and stabilizes the B-ring (Y256, F257, F268, F309, Y448) (Figure 4C), suggesting that aromatic packing is a key determinant of intermediate orientation.

To test this hypothesis, we performed site-directed mutagenesis followed by functional analysis in yeast (Figures 4G and S20). F360Y retained 85% of wild-type production of **2**, whereas Y256F, F257Y, Y256F/F257Y, and F309Y each reduced titers to <50%, indicating that perturbing the hydrophobic/aromatic environment disrupts productive binding and alignment. Notably, Y448F maintained overall yield but eliminated minor formation of **3**, producing **2** exclusively, while Y448A abolished activity. Together, these data identify Tyr448 as a key determinant of product selectivity, likely by stabilizing a carbocationic intermediate via a C–H···π interaction.

### An Asp273/Asp271 Acid–Base Pair Drives Class II Cyclization in AstC

The TC domain of AstC contains a conserved D^271^xD^273^T motif rather than the canonical DxDD sequence found in many class II TCs.^4c^ Structural evidence supports Asp273 as the Brønsted acid that initiates cyclization by protonating the terminal double bond of FPP (Figure 4D). Asp273 is positioned in a productive geometry through hydrogen bonds to N324 and Y361, which orient its carboxylate toward the terminal olefin. This protonation network closely mirrors that proposed for the bacterial drimenol synthase AsDMS, where an analogous Asp/Asp/Tyr arrangement plays the same initiating role,^14^ and similar positioning interactions have been described in other class II TCs, including SsDMS^10^ and MstE.^22^ Consistent with this assignment, the N324A and Y361F mutants completely abolished product formation (Figures 4G and S20).

By contrast, the identity of the deprotonating base varies across class II TCs (e.g., water-mediated bases,^10^ tyrosine,^24^ histidine,^25^ or aspartate^26^). Inspection of the AstC^•^FPP and AstC-TC^•^5 structures revealed no water molecules within 8 Å of the C2 position of bound FPP or the corresponded C9 of **5**, arguing against a water-based mechanism. Although Y256, Y446, and Y448 lie within 8 Å of the substrate, their phenolic groups point away from the reactive center (Figure 4C), and phenylalanine substitutions caused only moderate or variable effects on formation of **2** (Y256F ∼50% reduction; Y446F ∼23% reduction; Y448F no change; Figures 4G and S20), consistent with roles in ligand packing rather than general-base catalysis.

A nearby H447 (hydrogen-bonded to A359) resides within 8 Å of the substrate in both ligand-bound structures and is further stabilized upon **5** binding via a hydrogen bond to repositioned R452 (Figure 4E). However, H447A only partially decreased activity and altered product outcome by allowing formation of **3** alongside **2**, suggesting H447 primarily modulates product selectivity rather than serving as the principal base.

Instead, both structures and mutagenesis implicate Asp271 as the deprotonating base. Asp271 is strictly conserved among HAD-like TCs and lies 8.5 Å from C2 in the FPP-bound state, shortening to 6.6 Å (from the corresponding C9) in the **5**-bound state (Figure 4E), consistent with a trajectory that brings the cyclized intermediate closer to the base. Neighboring residues within the conserved D^271^xD^273^T motif, particularly D273 and T274, appear to help position the Asp271 carboxylate toward the substrate. Consistent with this model, D271A, D271N, and T274A substitutions abolished product formation (Figures 4G and S20), supporting an Asp273 (acid)/Asp271 (base) pair in AstC-catalyzed class II cyclization.

### Proposed Class II Cyclization Mechanism of AstC

Upon FPP binding, the AstC class II TC active site closes through coordinated rearrangements of K451 and Q265. K451 rotates into the pocket as a pyrophosphate-sensing residue, while Q265 reorients around the pyrophosphate (Figure 4F). The pyrophosphate is electrostatically neutralized by K314/K451 and further stabilized by a hydrogen-bond network involving T264, A449, and S450 (TC domain) plus S35 from the HAD-like domain of the partner monomer, resulting in tight anchoring at the TC/HAD-domain cleft. The farnesyl chain adopts a preorganized, cyclization-ready conformation, with C2–C7 and C6–C11 separations of 3.9 Å and 3.4 Å, respectively (Figure 3F).

Catalysis is initiated by Asp273 of the conserved D^271^xD^273^T motif. Positioned 3.2 Å from C10 and polarized by hydrogen bonds to N324 and Y361, Asp273 protonates the C10–C11 double bond to generate the initiating farnesyl cation. A cationic cascade then constructs the *trans*-decalin (drimane) scaffold. During this process, an aromatic environment including H447 and Y448 likely stabilizes developing carbocations via cation–π interactions and contributes to product selectivity. Termination occurs via site-specific deprotonation at C9 (drimane numbering) to yield **7**. We assign Asp271 as the Brønsted base for this final step: it lies 6.6 Å from C9 in the product-mimic structure, and T274 is positioned to orient and polarize the Asp271 carboxylate for proton abstraction. The observed separation likely reflects a prereactive state, with local side-chain motions bringing Asp271 into productive proximity during turnover.

An alternative possibility is a water-assisted quenching pathway, in which a transient water molecule, unresolved in the present structures, is positioned between Asp271 and the cationic intermediate as the immediate proton acceptor. In this scenario, Asp271, together with T274, may organize and polarize the water to enable deprotonation, thereby accounting for their critical roles despite the relatively long Asp271–C9 distance observed in the product-mimic structure.

### Structure-Guided Mutagenesis of AacA Reveals Cooperative Control of Drimane Product Selectivity

To probe how fungal HAD-like TCs control drimane scaffold formation, particularly double-bond placement after deprotonation, we performed structure-guided mutagenesis of AacA. Mutations were designed using a superposition of the AstC crystal structure with an AlphaFold2-predicted AacA model and sequence alignments of HAD-TC homologues (Figures 4I and S21). In AstC, H447 and Y448 flank C8 of the bicyclic intermediate and are implicated in carbocation stabilization and product selectivity. However, reciprocal swapping of the corresponding positions between AacA and AstC (AacA-R443H/F444Y/G445A and AstC-H447R/Y448F/A449G) abolished detectable product formation (Figures 4H and S22), indicating that these positions are not simply interchangeable and that carbocation stabilization alone does not dictate product outcome.

We next targeted aromatic residues lining the farnesyl-binding pocket. Three positions in AacA (Y306, Y357, and F444) differ from AstC, which forms product **2**. Substituting the AstC counterparts into AacA markedly reduced formation of **1**: Y306F and Y357F each retained 15% of wild-type titer, F444Y retained 60%, and all double/triple combinations produced <15% of **1**. Guided by differences between AacA and the DrtB enzyme that favors product **3**, we also examined Y253 and F444. Y253F alone had minimal impact, whereas Y253F/F444Y altered the product profile to a **1**:**3** ratio of 2.8:1.

To test whether phosphate recognition contributes to selectivity, we replaced S260 and P261 with the corresponding AstC residues. The S260L/P261T variant showed a near wild-type product profile. However, combining this substitution with Y253F and F444Y decreased overall titer and further shifted distribution (**1**:**3** = 1.9:1). A related mutant set restored total yield to near wild-type while maintaining this altered ratio. Together, these results indicate that farnesyl-chain positioning, pyrophosphate recognition, and carbocation-stabilizing residues act cooperatively to tune product selectivity in fungal HAD-like TCs.

### Phosphate-Release Kinetics Support *Trans* TC–HAD Coupling in Dimeric AacA

Steady-state phosphate (Pi) release kinetics were used to probe functional coupling between the TC and HAD domain activities of AacA (Table 1; Figures S23 and S24). Because Pi is generated only during the HAD-catalyzed hydrolysis of the phosphorylated intermediate (**4** to **5** and Pi), reactions initiated with **4** primarily report HAD turnover, whereas reactions initiated with FPP report the overall coupled pathway (FPP to **4** via TC, followed by **4** hydrolysis by HAD) and therefore depend on both TC chemistry and the efficiency of intermediate handoff/capture.

**Table 1:** Steady-State Kinetic Parameters of FPP and **4** in Reactions Catalyzed by AacA, AacA_D270N and AacA-HAD

enzyme	substrate	*K*_m_ (μM)	*k*_cat_ (s^–1^)	*k*_cat_/*K*_m_ (μM^–1^s^–1^)
**AacA**	FPP	31.8	0.041	12.84 × 10^–4^
	Low-FPP (25–75 μM)	11.2	0.031	27.49 × 10^–4^
	High-FPP (100–250 μM)	98.4	0.055	5.60 × 10^–4^
	**4**	125.9	1.936	153.77 × 10^–4^
**AacA_D270N**	FPP	336.4	0.096	2.84 × 10^–4^
	**4**	102.2	3.572	349.51 × 10^–4^
**AacA-HAD**	FPP	176.9	0.023	1.27 × 10^–4^
	**4**	42.86	2.511	585.86 × 10^–4^

In the solution homodimer, the TC site of protomer A is functionally coupled to the HAD site of protomer B, enabling interprotomer (*trans*) transfer of **4** across the dimer interface. Full-length AacA hydrolyzes **4** rapidly (*k*_cat_ = 1.936 s^–1^) but with a relatively high apparent *K*_m_ for **4** (125.9 μM), consistent with reduced accessibility of externally supplied **4** in the intact, coupled assembly. In contrast, Pi formation from FPP is much slower (*k*_cat_ = 0.041 s^–1^; *K*_m_ = 31.8 μM), indicating that TC-mediated **4** generation and interprotomer delivery is rate-limiting for overall turnover. This is reinforced by the large difference in catalytic efficiency (*k*_cat_/*K*_m_ = 153.77 × 10^–4^ for **4** versus 12.84 × 10^–4^ s^–1^ μM^–1^ for FPP), implying that HAD chemistry is intrinsically fast but upstream formation/transfer of **4** constrains flux from FPP.

Notably, the FPP dependence is biphasic, with a low-FPP regime (*K*_m_ = 11.2 μM; *k*_cat_ = 0.031 s^–1^) and a high-FPP regime (*K*_m_ = 98.4 μM; *k*_cat_ = 0.055 s^–1^), consistent with a coupled multistep mechanism and/or a shift in the dominant limitation as substrate increases. The standalone HAD domain provides an important control: it hydrolyzes **4** efficiently (*K*_m_ = 42.86 μM; *k*_cat_ = 2.511 s^–1^) but shows weaker activity on FPP (*K*_m_ = 176.9 μM; *k*_cat_ = 0.023 s^–1^), indicating that direct HAD-on-FPP turnover could contribute to Pi release when coupling is disrupted.

Accordingly, the TC-impaired mutant AacA_D270N shows a markedly increased apparent *K*_m_ for FPP (336.4 μM) yet retains Pi-releasing activity (*k*_cat_ = 0.096 s^–1^), while still turning over **4** rapidly (*K*_m_ = 102.2 μM; *k*_cat_ = 3.572 s^–1^). Overall, these trends support kinetically coupled TC and HAD functions in a dimeric (*trans*) architecture that favors interprotomer use of **4** relative to diffusion-based capture. This behavior is conceptually analogous to tryptophan synthase, in which the channeling-competent complex shows reduced accessibility to an externally supplied intermediate relative to the uncoupled domain.^27^

### Divergent Processing of Farnesyl Mono- and Thiopyrophosphate Analogues by AstC and AacA

Guided by structural and mechanistic insights from AstC, we probed the reactivity of AstC and AacA toward **6** and farnesyl thiopyrophosphate (FSPP) (Figure 5). The AstC-TC^•^5 structure shows that a monophosphate can be stabilized in an FPP-like pose via interactions with K314 and S450, implying that **6** can bind in the same pocket. Next, incubating **6** with AacA or AstC produced **5** and **8**, respectively, demonstrating that both enzymes cyclize **6** while preserving their distinct product selectivities (Figures 5A, S25 and S26). Time-course experiments (1–15 min) revealed only partial turnover of **6**, whereas FPP was fully consumed within 2 min, establishing **6** as a substantially less efficient substrate. Notably, in both cases the cyclized monophosphate esters were not further dephosphorylated.

**Figure 5:**
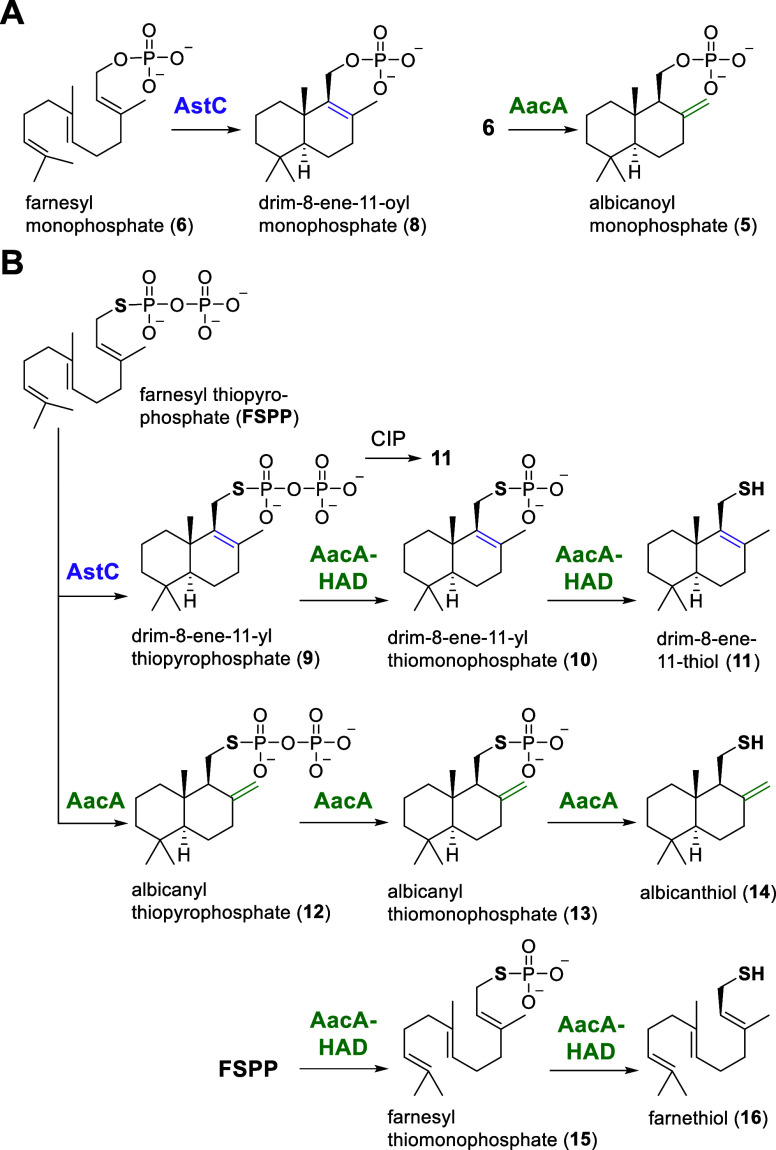
Function of AstC, AacA and AacA-HAD with (A) farnesyl mono- and (B) thiopyrophosphate analogues.

Using FSPP, AstC generated drim-8-ene-11-yl thiopyrophosphate (**9**) (Figures 5B and S27). CIP converted **9** to drim-8-ene-11-thiol (**11**), and EI–MS of **11** closely matched that of **2** while exhibiting a diagnostic thiol molecular ion (*m*/*z* 238.2), confirming thiol formation (Figure S30). Thus, AstC efficiently cyclizes FSPP to a thiopyrophosphate that can be hydrolyzed to the corresponding thiol by CIP.

In contrast, AacA displayed expanded reactivity toward FSPP. LC–MS detected albicanyl thiomonophosphate (**13**) and farnesyl thiomonophosphate (**15**), while GC–MS revealed albicanthiol (**14**) and farnethiol (**16**) (Figure S28), again with thiol-derived ions at *m*/*z* 238.2 (Figure S30). Importantly, EDTA led to accumulation of albicanyl thiopyrophosphate (**12**), indicating that Mg^2+^ is required for sequential dephosphorylation by the AacA HAD-like domain. This differs from FPP-derived intermediates (β-phosphate cleavage only) and likely reflects altered electronics/metal interactions imposed by the bridging P–S bond. Finally, pairing AstC with the AacA HAD-like domain in FSPP reactions yielded drim-8-ene-11-yl thiomonophosphate (**10**) alongside **11** (Figure S27), showing that non-native TC/HAD coupling can tune both scaffold output and phosphorylation state (thiopyrophosphate, thiomonophosphate, or free thiol).

To investigate the distinct processing of oxo- and thio-substrates by AacA, we performed molecular docking using an AlphaFold3-predicted AacA model (Figure S29). The phosphate/metal-binding center is conserved relative to AsDMS, whereas the prenyl-binding pocket shows local differences. Both pyrophosphate and thiopyrophosphate substrates adopt similar prehydrolytic poses, with the β-phosphate positioned near N106/Mg^2+^ and catalytic D10. A key difference emerges after the first cleavage: native monophosphates dock away from the catalytic center, whereas thiomonophosphates remain productively oriented for α-phosphate cleavage. These results suggest that sulfur substitution alters headgroup electronics and interactions with the Mg^2+^-coordinating phosphate-binding center, thereby providing a structural rationale for the stepwise hydrolysis of thiophosphate substrates by AacA.

## Conclusion

We identified AacA from *A. aculeatus* as a bifunctional albicanoyl monophosphate synthase. Biochemical reconstitution showed that AacA catalyzes protonation-initiated (class II) cyclization of FPP to form intermediate **4**, followed by Mg^2+^-dependent hydrolysis of the terminal phosphate to yield **5**. In contrast, AstC functions as a drim-8-ene-11-yl pyrophosphate synthase that lacks phosphatase activity, although its HAD-like domain coregulates cyclization together with the TC domain.

Notably, we determined the X-ray crystal structures of AstC, representing the first structures of a fungal HAD-like terpene cyclase. The substrate- and product-mimic structures reveal a head-to-tail homodimer in which the partner HAD-like domain caps the TC active site and positions the pyrophosphate at the intersubunit interface, consistent with trans TC-to-HAD intermediate transfer. Within the AstC TC active site, a ligand-responsive phosphate-sensing pair (Q265/K451), an aromatic cage in which H447/Y448 play key roles, and an Asp271/Asp273 acid–base pair together rationalize carbocation initiation, stabilization, and controlled termination of the drimane-forming cascade.

Structure-guided mutagenesis of AacA further showed that residues shaping the farnesyl-binding pocket, phosphate-recognition network, and cation-stabilizing environment cooperate to control double-bond placement and product outcome. In addition, phosphate-release kinetics for AacA support an analogous cross-monomer coupling mechanism in the bifunctional enzyme.

Finally, assays with farnesyl mono- and thiophosphate analogues revealed distinct dephosphorylation capacities: AacA supports stepwise thiophosphate hydrolysis, whereas AstC halts at the thiopyrophosphate stage, and non-native TC/HAD pairing can reprogram both terpene skeleton and phosphorylation state. Together, these findings provide new mechanistic insights into fungal DTS biosynthesis and open new opportunities for TC engineering and synthetic biology applications.

## Supplementary Material



## Notes

Experimental details and spectroscopic data (PDF)
