# Supplementary material for: Dimerization-Dependent Trans-Domain Coupling Enables Intermediate Transfer in Fungal Haloacid Dehalogenase-Like Terpene Cyclases
Source: J Am Chem Soc. 2026 May 15;148(21):22120–30. doi: 10.1021/jacs.6c04151 (PMC13244436; doi:10.1021/jacs.6c04151)
Supplement: Supplementary file 1 [file ja6c04151_si_001.pdf]

Supporting Information

Dimerization-Dependent Trans-Domain Coupling Enables Intermediate Transfer in Fungal Haloacid Dehalogenase-Like Terpene Cyclases

Tzu-Ho Chen,[‡] Kai-Fa Huang,[‡] Ting-Hung Chou,[§] Cheng-Chung Tseng,[‡+] Rou-Jie Huang,[¶] Tzu-Ping Ko,[‡] Suh-Yuen Liang,[‡] Rong-Jie Chein,[§※], and Hsiao-Ching Lin[‡+]*

[‡]Institute of Biological Chemistry, Academia Sinica, Taipei 115, Taiwan R.O.C.

[§]Biomedical Translation Research Center (BioTReC), Academia Sinica, Taipei 11529, Taiwan R.O.C.

[+]School of Pharmacy, College of Medicine, National Taiwan University, Taipei 100, Taiwan R.O.C.

[¶]Department of Chemistry, National Taiwan University, Taipei 106, Taiwan R.O.C.

[※]Institute of Chemistry, Academia Sinica, Taipei 11529, Taiwan R.O.C.

Table of Contents

Table of Contents ii

Experimental Procedures 4

General culture conditions 4

General molecular cloning 4

Homologs identification, sequence clustering, and phylogenetic tree analysis 4

Chemical Analysis 5

Construction of plasmids for *drtB*, *aacA*, and *astC* expression in *S. cerevisiae* 5

Construction of plasmids for full-length, mutant, and truncated *aacA* and *astC* expression in *E. coli* 5

Purification of recombinant full-length, mutant, and truncated AacA and AstC 5

Protein identification 6

In vitro enzyme reactions 7

Chemical Analysis of Transformed *S. cerevisiae* 8

Absolute protein molecular weight determination by SEC-MALS 8

Protein Crystallization, data collection, and structure determination 8

General information on the chemical synthesis 9

((8aS)-5,5,8a-trimethyl-2-methylenedecahydronaphthalen-1-yl)methyl dihydrogen phosphate bisammonium salt 9

(2E,6E)-3,7,11-trimethyldodeca-2,6,10-trien-1-yl phosphate bisammonium salt 10

(2E,6E)-3,7,11-trimethyldodeca-2,6,10-trien-1-yl diphosphate triammonium salt 10

Supplementary Tables 11

Table S1. X-ray data collection and refinement statistics. 11

Table S2. Homologous HAD-like TCs in the phylogenetic tree in Figure 1F. 12

Table S3. Primers used in this study 13

Table S4. Mass spectrometric protein identification of AacA and AstC variants. 16

Table S5. Mass spectrometric protein identification of major co-purified *E. coli* host proteins in AacA-TC and AstC-TC. 17

Supplementary Figures 18

Figure S1. Overview of the architectures of class I and class II terpene cyclases. 18

Figure S2. GC–MS analysis of fungal HAD-like terpene cyclase homologs expressed in *S. cerevisiae*. 19

Figure S3. SDS-PAGE of purified recombinant proteins AacA and its variants. 20

Figure S4. LC–MS and GC–MS analysis of i*n vitro* FPP conversion by AacA. 21

Figure S5. Amino acid sequence alignment of HAD-like TCs. 22

Figure S6. LC–MS analysis of *in vitro* FPP conversion by AacA_D270N time course. 23

Figure S7. LC–MS analysis of *in vitro* compound **4** conversion by AacA_D270N time course. 24

Figure S8. LC–MS and GC–MS analysis of *in vitro* assays of **5** with AacA_D270N time course. 25

Figure S9. LC-MS analysis of EDTA effects on AacA- and AacA_D270N-catalyzed FPP conversion. 26

Figure S10. SDS-PAGE of purified recombinant AstC and its variants. 27

Figure S11. LC–MS and GC–MS analysis of *in vitro* assays of FPP with AstC time course. 28

Figure S12. LC–MS and GC–MS analysis of *in vitro* assays of **4**, or **5** with AstC. 29

Figure S13. LC–MS and GC–MS analysis of *in vitro* assays of FPP or **4** with AstC_D273N. 30

Figure S14. SEC–MALS analysis of AstC_D273N. 31

Figure S15. Structural comparison of AstC with other class II terpene cyclases. 32

Figure S16. Comparison of the dimeric architectures of AstC and AsDMS. 33
Figure S17. Structural superposition of the AstC crystal structure and an AlphaFold2-predicted AacA model. 34
Figure S18. SEC–MALS analysis of AacA_D270N. 35
Figure S19. Amino acid sequence alignment of HAD-like enzymes. 36
Figure S20. GC-MS of AstC mutants expressed in *S. cerevisiae*. 37
Figure S21. Superposition of the AstC-TC•**5** structure with an AlphaFold2-predicted AacA model docked with **4**. 38
Figure S22. GC-MS of AacA mutants expressed in *S. cerevisiae*. 39
Figure S23. Michaelis-Menten plots of AacA, AacA_D270N, and AacA-HAD with substrate **4**. 40
Figure S24. Michaelis-Menten plots of AacA, AacA_D270N, and AacA-HAD with FPP. 41
Figure S25. LC–MS and GC–MS analysis of *in vitro* assays of **6** with AstC time course. 42
Figure S26. LC–MS and GC–MS analysis of *in vitro* assays of **6** with AacA time course. 43
Figure S27. LC–MS and GC–MS analysis of *in vitro* assays of FSPP with AacA-HAD, or AstC with or without AacA-HAD. 44
Figure S28. LC–MS and GC–MS analysis of *in vitro* assays of FSPP with AacA, or AacA and EDTA. 45
Figure S29. Structural comparison and docking analysis rationalizing selective α-phosphate hydrolysis of thiophosphate substrates by AacA. 46
Figure S30. GC–MS analysis and the EI mass spectra of FSPP or FPP derived compounds, **11**, **2**, **14**, **1**, **16**, and farnesol. 48
Figure S31. LC–MS analysis and the mass spectra of FPP, **6**, **4**, **5**, and FSPP. 49
Figure S32. ^{1}H NMR spectrum of **4** (CD_3OD, 600 MHz). 50
Figure S33. ^{13}C NMR spectrum of **4** (CD_3OD, 150 MHz). 51
Figure S34. ^{31}P NMR spectrum of **4** (CD_3OD, 243 MHz). 52
Figure S35. ^{1}H NMR spectrum of **6** (CD_3OD, 600 MHz). 53
Figure S36. ^{31}P NMR spectrum of **6** (CD_3OD, 243 MHz). 54
Figure S37. ^{1}H NMR spectrum of FPP (D_2O, 600 MHz). 55
Figure S38. ^{31}P NMR spectrum of FPP (D_2O, 243 MHz). 56
References 57

Experimental Procedures

General culture conditions

Escherichia coli DH10B (Invitrogen) was used for cloning, while BL21 was employed for protein expression and purification. *E. coli* strains were cultured in LB medium (25 g/L Luria–Bertani broth powder in D.I. water) at 37 °C with constant orbital shaking at 200 rpm. For yeast expression, *Saccharomyces cerevisiae* BJ5464-NpgA yeast strain was used to heterologously express HAD-like terpene synthase genes. Yeast cells were cultured in YPD medium (10 g/L yeast extract, 20 g/L peptone, 1% glucose) at 28 °C under shaking at 200 rpm.

General molecular cloning

Standard molecular cloning procedures were performed as previously described.[1] Briefly, gene fragments were amplified using Q5® High-fidelity DNA polymerase (New England Biolabs). PCR products were resolved by agarose gel electrophoresis and purified using Zymoclean™ Gel DNA Recovery Kit (Zymo Research). DNA fragments were assembled using NEBuilder® HiFi DNA Assembly Cloning kit (New England Biolabs) and transformed *E. coli* DH10B competent cells. Plasmids were extracted and subjected to DNA sequencing to confirm the correct constructs.

Homologs identification, sequence clustering, and phylogenetic tree analysis

Homologs of AstC were identified with BLASTp[2] against NCBI nr database at https://blast.ncbi.nlm.nih.gov/. A total of 2250 homologs with expected p values < E-10 were identified and protein sequences were downloaded from the website. The homologs of AstC were combined with the six proteins of interest (total 2257 sequences) to build a database with which each of the 2257 sequences was aligned to all in this database with BLASTp on in-house server. The expected p values calculated for each paired sequence alignment from BLASTp were transformed by taking the logarithm in base 10 and subsequently negating the sign. The transformed values are capped so that any E-value below 1e-1000 is set to a maximum allowed edge weight of 1000. A data matrix of 2363 by 2363 with the transformed p values was used for sequence clustering using Markov Cluster algorithm v14-137 [3] with the inflation rate (I) set to 2.5. One of the sequence clusters from MCL which contained 1598 homologs and included all seven proteins of interest was considered for phylogenetic tree analysis. To further reduce the number of homologs, the redundant or similar sequences with identity > 0.7 were first grouped and the longest sequence was chosen as the representative for the group using CD-hit at http://weizhong-lab.ucsd.edu/cdhit_suite/cgi-bin/index.cgi [4]. A total of 625 homologs of AstC with the sequence length greater or equal to 250 amino acids were used to build the phylogenetic tree with ETE3 v.3.1.2 [5] using MAFFT v6.861b [6] for multiple sequence alignment and FastTree v.2.1.8 [7] for tree building. Treeshrink v 1.3.9 [8] was used with alpha equal to 0.1 to remove the outliers (five) which had unrealistically long branch lengths in the phylogenetic tree. Final tree plot in the fan layout was generated with ggtree [9] package in R v. 4.5.1 [10] and the tree branches were colored according to the phylum which the protein homolog is belong to based on the NCBI taxonomy database. The protein homologs missing the information of phylum in the database were removed from the tree. The phylogenetic tree was further trimmed down with Treemmer v. 0.3 [11] to generate a simplified tree (27 homologs) based on the clades found in the tree with pre-determined maximal number of branches per clade.

Chemical analysis

All solvents and chemicals used were of analytical grade. All LC-ESIMS analyses were performed on a Shimadzu 2050 LC-MS (Kinetex® 2.6 µm Polar C18 100 Å, 100 x 2.1 mm column) using positive and negative mode electrospray ionization with linear gradients of 5–50% acetonitrile (MeCN)-H_2O in 5 minutes, and 50–70% MeCN-H_2O in 5 minutes followed by 70% MeCN-H_2O for 4 minutes with a flow rate of 0.5 mL/min, the mobile phase is constantly supplied with 10 mM ammonium bicarbonate.

All GC-EIMS analyses were performed on an Agilent 7890B GC with a 5977B MSD and a PAL RTC autosampler with Cyclosil-B column (30 m, 0.25 mm i.d., 0.25 µm film, Agilent Technologies). The inlet and MSD transfer line temperature were both set at 250°C. A flow rate of 2 mL/min of helium was used as carrier gas and electronic impact was 70 eV. An overall GC cycle run time was set for 13 minutes as followed: oven temperature was programmed at 80°C hold for 1 minute, from 80°C to 220°C in 7 minutes (ramp 20°C/min), and 220°C hold for 5 minutes. For solid-phase microextraction (SPME), the samples were extracted for 5 minutes at 80°C and desorbed for 2 minutes at 240°C in the injection port.

Construction of plasmids for *drtB*, *aacA*, and *astC* expression in *S. cerevisiae*

The cDNA of *drtB*[12] was obtained by gene synthesis. The *aacA* and *astC* were obtained by RT-PCR using ImProm-IITM Reverse Transcription System (Invitrogen) to synthesize complementary DNA (cDNA) from total RNA. Target genes were cloned into pXW55 yeast expressing vector by assembly the insert gene with linearized vector using the method described in General molecular cloning section. To generate point-mutated variants of *aacA* and *astC*, site-directed mutagenesis was performed using specific primers (summarized in **Table S3**) and the wild-type constructs serving as templates. The resulting PCR fragments were cloned into the XW55 vector to generate mutant variants for yeast expression.

Construction of plasmids for full-length, mutant, and truncated *aacA* and *astC* expression in *E. coli*

The full-length, single-point mutant variant, or truncated *aacA and astC* genes were obtained by standard PCR amplification from the as-described pXW55 constructs, and cloned into linearized pCold^{TM} I vector (Takara Bio) for *E. coli* expression. Truncated *aacA* and *astC* compose of only the terpene cyclase domain sequences, residues Gly^{196}–Glu^{466}, and residues Asp^{200}–Asn^{475}, respectively, were amplified from their corresponding full-length constructs and cloned into pET30 vector. Plasmids were then propagated in *E. coli* DH10B, then isolated and confirmed by sequencing. Verified constructs were then transformed into *E. coli* BL21 (DE3) strain for protein expression. Primers used for constructing *E. coli* expression plasmids were summarized in **Table S3**.

Purification of recombinant full-length, mutant, and truncated AacA and AstC

Cell lysis procedures were described elsewhere. In brief, transformed *E. coli* BL21 (DE3) harboring AacA, AacA_D270N, AacA-TC, AstC, AstC_D273N, and AstC-TC expressing plasmids were seed cultured in 10 mL of LB medium and incubated at 37°C, 200 rpm constant shaking for 16 hours. Seed cultures were then transferred to 1 L fresh LB for further incubation under the

same conditions until optimal cell density reached (OD_{600} 0.4–0.6). The cells were then cooled to 16°C and induction of protein expression by adding final 0.1 mM isopropylthio-β-D-galactoside (IPTG), then incubate under 16°C, 200 rpm constant shaking for 16 hours. Pellet the cells by centrifugation and resuspend in 100 mL Buffer A, which composed of 20 mM Tris, pH 8, 500 mM NaCl, and 10 mM imidazole. Cells were then lysed by passing through a NanoLyzer N-2 High-Pressure Homogenizer twice under 18 kpsi. The subsequent lysate was centrifuged, and filtered the supernatant through 0.22 μm membrane. The lysate was then loaded to a gravity-flow column filled with 1 mL Ni^{2+}-charged Nuvia™ IMAC Resin (Bio-Rad) followed by Buffer A wash and stepwise gradient with increased contents of Buffer B (20 mM Tris, pH 8, 500 mM NaCl, 500 mM imidazole) to wash-off non-specific unbound proteins. Target proteins were eluted at imidazole concentration up to 150 mM. Purified proteins were concentrated and exchanged to storage buffer (50 mM Tris-HCl, pH 7.5, 5% glycerol) using an Amicon Ultra-30 Centrifugal Filter Unit, freeze under liquid nitrogen and stored under −80 °C for further uses.

For crystallization, proteins obtained from Ni^{2+} affinity purification are further purified by size exclusive chromatography Superdex 200 Increase 10/300 GL (Cytiva) column using NGC Discover 10 chromatography system (Bio-Rad). The proteins are concentrated to ~ 10 mg/mL with 0.5 mL total volume and manually injected to the column, followed by elution under 50 mM Tris-HCl, pH 7.5 buffer and collected by fraction collector. Fractions containing the target protein are confirmed by protein gel and pooled. The as purified proteins were4 concentrated to 5 – 10 mg/mL suitable for crystallization, and the concentration was measured by Nano-photometer N60 at 280 nm absorbance according to their molar extinction coefficient.

Protein identification

Protein in-gel digestion: The protein band on 1D gel was manually excised from the gel and cut into pieces and distained with 25% acetonitrile in 25 mM ammonium bicarbonate. The gel pieces were reduced with 50 mM DTE in 25 mM ammonium bicarbonate, pH 8.5, at 37°C for 1 hour, and subsequently alkylated with 100 mM iodoacetamide in 25 mM ammonium bicarbonate, pH 8.5, at room temperature in dark for 1 hour. The gel pieces were then washed with 25% acetonitrile in 25 mM ammonium bicarbonate, pH 8.5 for 5 minutes once, dehydrated with acetonitrile for 5 minutes and dried under vacuum. The pieces were rehydrated with 1:50 enzyme to protein ratio of Mass Spectrometry Grade Lys-C (Wako, 125-05061) in 25 mM ammonium bicarbonate, pH 8.5, digested at 37°C for 3 hours. Following Lys-C digestion, same amount of sequencing grade Trypsin (Promega, Madison, WI, USA) in 25 mM ammonium bicarbonate, pH 8.5, was added and digested at 37°C for 16 hours. Tryptic peptides were extracted twice with 50% acetonitrile containing 5% TFA for 3 min each time with moderate sonication. The extracted solutions were pooled and evaporated to dryness under vacuum. The peptide mixture was aliquoted, desalted, and concentrated on a C18-ZipTip (Millipore), and eluted with 50% acetonitrile in 0.1% formic acid.

Protein in-solution digestion: The samples were reduced with 10 mM DTE in 25 mM ammonium bicarbonate and ~6.9 M urea in 25 mM ammonium bicarbonate, pH 8.5, at 37°C for 1 hour, and subsequently alkylated with 25 mM iodoacetamide in 25 mM ammonium bicarbonate, pH 8.5, at room temperature in dark for 1 hour. Then the reaction was quenched with 25 mM DTE in 25 mM ammonium bicarbonate. The samples were digested with 1:50 enzyme to protein ratio of Mass Spectrometry Grade Lys-C (Wako, 125-05061) in 25 mM ammonium bicarbonate, pH 8.5, at 37°C for 3 hours with Urea concentration lower than 4 M. Following Lys-C digestion, same amount of

sequencing grade Trypsin (Promega, Madison, WI, USA) in 25 mM ammonium bicarbonate, pH 8.5, was added and digested at 37°C for 16 hours while urea concentration was lower than 1 M. The digestion reaction was quenched with 0.1% formic acid then evaporated to dryness under vacuum. The peptide mixture was aliquoted, desalted, and concentrated on a C18-ZipTip (Millipore), and eluted with 50 % acetonitrile in 0.1% formic acid.

Shotgun proteomic identifications: NanoLC−nanoESI-MS/MS analysis was performed on a nanoAcquity system (Waters, Milford, MA) connected to the LTQ Orbitrap Velos hybrid mass spectrometer (Thermo Electron, Bremen, Germany) equipped with a Thermo Nanospray Flex Ion Sources interface. Peptide mixtures were loaded onto a 75 μm ID, 25 cm length C18 BEH column (Waters, Milford, MA) packed with 1.7 μm particles with a pore with of 130 Å. Samples AstC_D271A, AstC_D271N, AacA_D268A and AacA_D268N were separated using a 30 minutes segmented gradient that increased solvent B (0.1% formic acid in acetonitrile) from 5% to 25% in 27.5 minutes, followed by 25% to 35% in 2.5 minutes, at a flow rate of 300 nl/min. Solvent A was 0.1% formic acid in water. Samples AstC (residues Asp^{200}–Asn^{475}) and AacA (residues Gly^{196}–Glu^{466}) were separated using a 90 minutes segmented gradient that increased solvent B from 5% to 25% in 82.5 minutes, followed by 25% to 35% in 7.5 minutes, at a flow rate of 300 nl/min. Solvent A and Solvent B were the same and column temperature was set as 35°C for both sets of samples. The mass spectrometer was operated in the data-dependent mode. Briefly, survey full scan MS spectra were acquired in the orbitrap (*m/z* 350–1600) with the resolution set to 60K at *m/z* 400 and automatic gain control (AGC) target at 10^6. The 20 most intense ions were sequentially isolated for CID MS/MS fragmentation and detection in the linear ion trap (AGC target at 10^4) with previously selected ions dynamically excluded for 60 seconds. Ions with singly and unrecognized charge state were also excluded. The MS and MS/MS raw data were processed by Proteome Discoverer (v 3.2.0.450; Thermo Scientific, Waltham, MA, USA) and searched against Swiss-Prot protein sequence database, a custom protein sequence database and cRAP contaminate database with the Mascot server (v.3.1.0; Matrix Science, Boston, MA, USA). Taxonomy was set as *Escherichia coli*. Search criteria used were trypsin digestion, static modifications set as carbamidomethyl (C), dynamic modifications set as oxidation (M) and allowing up to 2 missed cleavage, mass accuracy of 10 ppm for the parent ion and 0.6 Da for the fragment ions mass tolerance.

In vitro enzyme reactions

In vitro enzymatic reactions of AacA, AacA_D270N, AacA-TC, AstC, AstC_D273N, and AstC-TC were generally performed, unless otherwise noticed, using 1 μM enzymes (for boiled control, the enzyme is incubated 5 minutes on a 95℃ heating block) in a 125 μL buffer solution composed of 100 μM various of substrates (FPP, FsPP, compound **4**, **5**, and **6**), 100 mM Tris-HCl, pH 7.5, and 0.5 mM $MgCl_2$. After addition of the enzyme to the mixture, allowed the solution mixture to inoculate under room temperature for given time, aliquot 25 μL of the in vitro reaction mixture to a vial containing 30 μL acetonitrile to quench the reaction for LC-ESIMS analysis. For LC-ESIMS, samples were centrifuged 5 minutes (17,000 x *g*) and the supernatant was transferred to a sample vial, inject 8 μL for analysis. The remaining 100 μL reaction directly analyzed by SPME-GC-MS. For calf alkaline phosphatase (Quick CIP, New England Biolabs) treatment, the reactions were further incubated with the presence of 5 units of CIP (5 unit/μL) for 30 minutes prior to quenching or SPME-GC-MS.

Chemical analysis of transformed *S. cerevisiae*

Transformed yeasts harboring pXW55 (Uracil marker) with insert gene *drtB*, *aacA*, *astC*, and their mutant variants were plated on synthetic drop-out medium solid agar for 3 days to form colonies. Single-colony was picked and inoculated into 2 mL synthetic drop-out selection medium (minus uracil) and incubated at 28 °C with constant shaking at 200 rpm for 3 days. Aliquot 6 µL seed culture to inoculate into 3 mL YPD medium, culture under 28 °C with constant shaking at 200 rpm for 3 days. Transfer 100 µL crude culture mixtures to sample vials for SPME-GC-MS for volatile natural products analysis. Compounds were than identified according to the NIST database. For yeasts expressing *aacA* and *astC* mutant variants, the relative yield of drimanyl compounds were quantified by their corresponding TIC peak area under curve.

Absolute protein molecular weight determination by SEC-MALS

The size exclusion chromatography-multi-angle static light scattering (SEC-MALS) measurement was performed using 1260 Infinity HPLC system (Agilent) coupled to in-line detectors: laser light scattering (miniDAWN TREOS, Wyatt), quasi-elastic light scattering (QELS, Wyatt), refractive index (Optilab T-rEX, Wyatt), and UV detection. A 100 µL purified protein at 1 mg/mL concentration was injected onto an ENrich SEC 650 column (Bio-rad) pre-equilibrated with 50 mM Tris, pH7.5 buffer. Bovine serum albumin (BSA) was used for system calibration. The analysis was performed under a flow rate of 0.5 mL/min, and data acquisition was done by ASTRA 6 software (Wyatt) with the dn/dc value set at 0.185 mL/g.

Protein Crystallization, data collection, and structure determination

Two-domain AstC (residues Ile^{4}–Asn^{475} with D273N single mutation) and its C-terminal terpene cyclase domain (AstC-TC, Asp^{200}–Asn^{475}, D273N) were prepared in 50 mM Tris-HCl, pH 7.5, and concentrated to ~10 mg/mL. To obtain the AstC•FPP complex, $AstC^{4\text{-}475}$_D273N was incubated with 2 mM FPP on ice for ~ 1 hour prior to crystallization. Crystallization screening was conducted using a Phoenix RE crystallization robot (Rigaku), testing ~800 conditions. Crystals suitable for X-ray diffraction were obtained under the following conditions: $AstC^{4\text{-}475}$_D273N, 20% (w/v) PEG 4000, 100 mM HEPES, pH 7.5, and 10% (v/v) 2-propanol; $AstC^{200\text{-}475}$_D273N, 0.1 M sodium cacodylate trihydrate pH 6.5, 1.4 M Sodium acetate trihydrate. Crystals were grown at 20 °C by mixing equal volumes of protein solution and crystallization buffer via the sitting-drop vapor-diffusion method. To obtain the AstC-TC•**5** complex, crystals of the C-terminal domain were soaking in the mother liquor containing 5 mM AMP (cpd **5**). X-ray diffraction data were collected at beamlines TLS 15A1, TPS 05A1, or TPS 07A of the National Synchrotron Radiation Research Center (NSRRC), Hsinchu, Taiwan. Data were processed and scaled with the *HKL2000* package.[13] The crystals of AstC•FPP and AstC-TC belonged to the space group $P2_12_12_1$ and $P6_122$, respectively, with two AstC chains per asymmetric unit in both cases. Data collection statistics are summarized in **Table S1**.

The structure of full-length AstC was solved by the molecular replacement phasing method with the program *Phaser* within the *CCP4* software suite,[14] employing the AlphaFold 3-predicted model (https://alphafold.ebi.ac.uk/entry/AstC) as the search template.[15] The initial model was manually adjusted with *Coot*,[16] guided by $2F_o$-F_c and F_o-F_c maps, and further refined computationally with *Phenix*.[17] The final electron densities enabled model building for residues Pro^{6}–Gln^{473} in chain A

and Pro[6]–Ala[474] in chain B, as well as placement of 2 FPP, 1 HEPES, and 299 water molecules in the density maps. The structures of the C-terminal domain were determined by employing the refined AstC structure. The structural quality was evaluated with MolProbity.[18] Final refinement statistics are provided in **Table S1**. The structures have been deposited in the Protein Data Bank under accession number 22TZ, 22TY, and 22UA.

General information on the chemical synthesis

All reactions involving air- or moisture-sensitive reagents were carried out in flame-dried glassware under an atmosphere of dry nitrogen, using standard syringe–septum techniques. Anhydrous solvents were freshly dried and purified by conventional methods before use. Reaction progress was monitored by thin-layer chromatography (TLC) on silica gel 60 F_{254} precoated glass plates (Merck). ^{1}H, ^{13}C, and ^{31}P NMR spectra were recorded on a Bruker Ascend 600 MHz spectrometer equipped with an AVANCE III HD console and a BBFO probe. Chemical shifts (δ) are reported in parts per million (ppm) relative to the residual solvent peaks: D_2O (δ 4.79 ppm in ^{1}H NMR), MeOD (δ 3.31 ppm in ^{1}H NMR and δ 49.0 ppm in ^{13}C NMR). Coupling constants (J) are given in Hertz (Hz), and signal multiplicities are designated as follows: s = singlet, d = doublet, t = triplet, m = multiplet.

((8a*S*)-5,5,8a-trimethyl-2-methylenedecahydronaphthalen-1-yl)methyl dihydrogen phosphate bisammonium salt

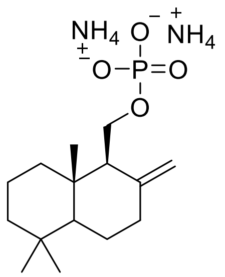

Trichloroacetonitrile (24 μL, 0.234 mmol) was added rapidly to a stirred solution of albicanol1 (8 mg, 0.036 mmol) and tetra-n-butylammonium dihydrogen phosphate (61 mg, 0.18 mmol) in dichloromethane (1 mL). The reaction mixture was stirred at RT for 2 hours, and then the solvent was removed in vacuo. The residue was dissolved in THF (1 mL), and 25% ammonium hydroxide solution in water (0.1 mL) was added. After 30 min, 1:1 toluene:methanol (5 mL) was added and the mixture stirred for a further 20 min. After this time, the resultant precipitate was removed by filtration. The solvent was removed in vacuo, the residue was washed with petroleum ether, dissolved in methanol (2 mL), and filtered. Excess Dowex 50WX8 (NH_4^+ form) was added, and the mixture was stirred for 30 min and then filtered. The solvent was removed in vacuo, and the residue was purified by flash column chromatography on silica gel (water:isopropanol:ethyl acetate = 0.3:1:1, Rf = 0.47), affording the ammonium salt (3.6 mg, 30%). ^{1}H NMR (600 MHz, MeOD) δ 4.83 (s, 1H), 4.78 (s, 1H), 4.15 (m, 1H), 4.00 (m, 1H), 2.38 (m, 1H), 2.03 (m, 1H), 1.84 (m, 1H), 1.74 (m, 1H), 1.60 (m, 1H), 1.49 (m, 1H), 1.35 (m, 3H), 1.20 (m, 3H), 0.89 (s, 3H), 0.84 (s, 3H), 0.78 (s, 3H); ^{13}C NMR (150 MHz, MeOD) δ 148.2, 108.3, 71.4, 62.9 (d, J = 5.2 Hz), 58.0

(d, J = 7.8 Hz), 56.5, 43.3, 40.3, 40.0, 38.9, 34.4, 25.2, 22.2, 20.3, 15.7; 31P NMR (243 MHz, MeOD) δ 1.18.

(2*E*,6*E*)-3,7,11-trimethyldodeca-2,6,10-trien-1-yl phosphate bisammonium salt

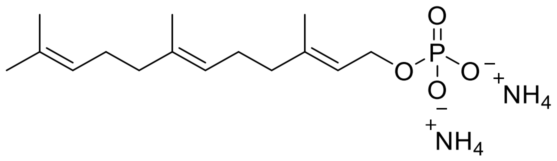

Trichloroacetonitrile (150 μL, 1.5 mmol) was added rapidly to a stirred solution of farnesol (50 mg, 0.225 mmol) and tetra-n-butylammonium dihydrogen phosphate (424 mg, 1.25 mmol) in dichloromethane (3.5 mL). The reaction mixture was stirred at RT for 1 hour, and then the solvent was removed in vacuo. The residue was dissolved in THF (2 mL), and 25% ammonium hydroxide solution in water (0.2 mL) was added. After 30 min, 1:1 toluene:methanol (5 mL) was added and the mixture stirred for a further 20 min. After this time, the resultant precipitate was removed by filtration. The solvent was removed in vacuo, the residue was washed with petroleum ether, dissolved in methanol (2 mL), and filtered. Excess Dowex 50WX8 (NH_4^+ form) was added, and the mixture was stirred for 30 min and then filtered. The solvent was removed in vacuo, and the residue was purified by flash column chromatography on silica gel (water:iso-propanol:ethyl acetate = 0.3:1:1, Rf=0.43) to afford the ammonium salt (63 mg, 83%). ^{1}H NMR (600 MHz, MeOD) δ 5.40 (t, J = 6.9 Hz, 1H), 5.14-5.06 (m, 2H), 4.44 (d, J = 6.5 Hz, 2H), 2.15-1.95 (m, 8H), 1.69 (s, 3H), 1.66 (s, 3H), 1.60 (s, 3H), 1.59 (s, 3H); ^{31}P NMR (243 MHz, MeOD) δ 0.93.

(2*E*,6*E*)-3,7,11-trimethyldodeca-2,6,10-trien-1-yl diphosphate triammonium salt

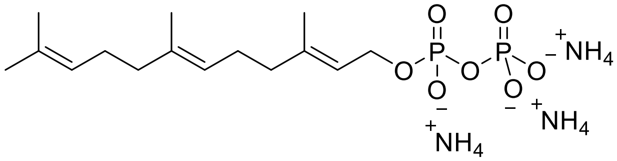

To a solution of farnesol (100 mg, 0.45 mmol) in trichloroacetonitrile (10 mL) was added a solution of bis-triethylammonium phosphate (10 mL) (TEAP; prepared by mixing 4.6 mL of a solution of 1.0 mL of phosphoric acid in 3.6 mL acetonitrile and 7.5 mL of a solution of 3.9 mL triethylamine in 3.6 mL acetonitrile). The reaction mixture was stirred at room temperature for 3 hours. Formation of the product was monitored by TLC (isopropanol/conc. NH_4OH/H_2O; 12/5/3, Rf = 0.3). Upon completion of the reaction, the solvent was removed, and the residue was purified by flash chromatography to afford the ammonium salt (10 mg, 5%). ^{1}H NMR (600 MHz, D2O) δ 5.46 (t, J = 6.9 Hz, 1H), 5.24-5.15 (m, 2H), 4.49 (d, J = 6.0 Hz, 2H), 2.20-2.00 (m, 8H), 1.73 (s, 3H), 1.69 (s, 3H), 1.63 (s, 3H), 1.62 (s, 3H); ^{31}P NMR (243 MHz, D2O) δ -10.7(m, 2P).

Supplementary Tables

Table S1. X-ray data collection and refinement statistics.

**Data collection**			
Crystal[a]	AstC•FPP	AstC-TC	AstC-TC•**5**
Sequence	Ile^{4}–Asn^{475}_D273N	Asp^{200}–Asn^{475}_D273N	Asp^{200}–Asn^{475}_D273N
Ligand	FPP	n.a. (apo form)	**5**
Space group	$P2_12_12_1$	$P6_122$	$P6_122$
Cell dimensions			
a, b, c (Å)	89.9, 104.2, 127.4	160.2, 160.2, 154.3	160.7, 160.7, 156.0
α, β, γ (°)	90.0, 90.0, 90.0	90.0, 90.0, 120.0	90.0, 90.0, 120.0
Resolution (Å)	30-2.95 (3.06-2.95)[b]	30-3.45 (3.57-3.45)[a]	30-2.85 (2.95-2.85)[a]
Unique reflection	25,641 (2,507)	15,869 (1,550)	28,137 (2,756)
Multiplicity	6.8 (5.5)	17.8 (16.8)	11.9 (9.2)
Completeness (%)	99.9 (99.2)	100.0 (100.0)	100.0 (100.0)
$I/\sigma(I)$	8.3 (1.2)	8.0 (1.6)	14.3 (1.8)
R_{pim}	0.107 (0.723)	0.086 (0.329)	0.091 (0.964)
**Refinement**			
Resolution (Å)	30-2.95	29.71-3.45	29.55-2.85
Reflections (> 0 $\sigma(F)$)			
Working/test	23,012/1,226	15,682/1,429	25,926/1,245
R_{work}/R_{free}[c]	0.172/0.229	0.217/0.271	0.258/0.288
RMS deviations			
Bond lengths (Å)	0.002	0.012	0.005
Bond angles (°)	0.59	1.31	0.84
B-factor ($Å^2$)/no. of atoms			
Protein	56.2/7,483	69.9/4,434	73.5/4,434
FPP or **5**	49.9/48		60.1/20
Other ligands	73.4/15	44.2/18	51.4/6
Solvent	44.7/229	26.9/145	52.9/164
MolProbity score	1.47	2.04	2.73
Clashscore	4.50	14.98	16.80
Ramachandran plot (%)[d]			
Favored	97.53	94.87	91.58
Allowed	2.47	5.13	8.42
Outliers	0	0	0
PDB ID code	22TZ	22TY	22UA

[a] All AstC samples carried the D273N mutation.
[b] Values in parentheses correspond to the highest resolution shell.
[c] Five percent of randomly selected data were set aside for calculating R_{free}.
[d] Calculated with MolProbity (http://molprobity.biochem.duke.edu)

Table S2. Homologous HAD-like TCs in the phylogenetic tree in Figure 1F.

Seq_ID	Species	Phylum
AstC	*Aspergillus oryzae*	Ascomycota
AacA	*Aspergillus aculeatus*	Ascomycota
AncA	*Antrodia cinnamomea*	Basidiomycota
AncC	*Antrodia cinnamomea*	Basidiomycota
AsDMS	*Aquimarina spongiae*	Bacteroidetes
DS3	*Termitomyces sp.*	Basidiomycota
DrtB	*Aspergillus calidoustus*	Ascomycota
XP_035346972.1	*Talaromyces rugulosus*	Eukaryota
KOS21320.1	*Escovopsis weberi*	Eukaryota
KAJ7609737.1	*Mycena polygramma*	Eukaryota
MCJ1454562.1	*Mycoblastus sanguinarius*	Eukaryota
XP_047853911.1	*Annulohypoxylon truncatum*	Eukaryota
KAK6860636.1	*Apiospora arundinis*	Eukaryota
KAL0571849.1	*Marasmius crinis-equi*	Eukaryota
TVY82214.1	*Lachnellula suecica*	Eukaryota
EJD42963.1	*Auricularia subglabra TFB-10046 SS5*	Eukaryota
KAF6761193.1	*Tulosesus angulatus*	Eukaryota
WP_362890005.1	*Streptomyces wuyuanensis*	Bacteria
KAF2090523.1	*Saccharata proteae CBS 121410*	Eukaryota
XP_024679696.1	*Aspergillus novofumigatus IBT 16806*	Eukaryota
KAF2847659.1	*Plenodomus tracheiphilus IPT5*	Eukaryota
XP_013330136.1	*Rasamsonia emersonii CBS 393.64*	Eukaryota
XP_016615570.1	*Cladophialophora bantiana CBS 173.52*	Eukaryota
MFW7381560.1	*Oligoflexus sp.*	Bacteria
MCA9707313.1	*Myxococcales bacterium*	Bacteria
HBO99618.1	*Candidatus Uhrbacteria bacterium*	Bacteria
MBN2291051.1	*Pirellulales bacterium*	Bacteria

Table S3. Primers used in this study

**Primer name**	**Sequence (5' → 3')**
**pXW55_*aacA***	
AacA_xw55_F	gattataaggatgatgatgataagactagtATGCCCTCCGTCAAAGCACTG
AacA _xw55_R	tgatggtgatggtgatgcacgtgTCGAATGTCAGTCGTCAATCCCCAC
**pXW55_*aacA* mutant variants**	
AacA_Y253F_F	GCCTTGGGATCGAACATGGAACTTCTTCATCGGGGTTCCTCAGTCGCCG
AacA_F254Y_F	GCCTTGGGATCGAACATGGAACTACTACATCGGGGTTCCTCAGTCGCCG
AacA_Y253F/F254Y_F	GCCTTGGGATCGAACATGGAACTTCTACATCGGGGTTCCTCAGTCGCCG
AacA_F253_flank_R	GTTCCATGTTCGATCCCAAGGC
AacA_S260L/P261T_F	CATCGGGGTTCCTCAGTTGACGACGAGCACCTTCCCCAAC
AacA_S260L/P261T/T262Q_F	CATCGGGGTTCCTCAGTTGACGCAGAGCACCTTCCCCAACGACC
AacA_S260_flank_R	ACTGAGGAACCCCGATGAAG
AacA_D268A_F	CGAGCACCTTCCCCAACGCCCTGGACACCACCTCTATCGCG
AacA_D268N_F	CGAGCACCTTCCCCAACAACCTGGACACCACCTCTATCGCG
AacA_D268_flank_R	GTTGGGGAAGGTGCTCGTCGG
AacA_D270N_F	GCACCTTCCCCAACGACCTGAACACCACCTCTATCGCGCTC
AacA_D270N_flank_R	CAGGTCGTTGGGGAAGGTGC
AacA_Y306F_F	CAACGACGGGATAGTCCCAACATTTTTGATCCCACTCGCCCTCGAG
AacA_Y306_flank_R	TGTTGGGACTATCCCGTCGTTG
AacA_R356A_F	GAGCCTACCTGCAGGGCACGGCATATTACATCAGTCCGGACGCCTTC
AacA_R356A_Flank_R	CGTGCCCTGCAGGTAGGCTC
AacA_R356A/Y357F_F	GAGCCTACCTGCAGGGCACGGCATTTTACATCAGTCCGGACGCCTTC
AacA_Y357F_F	GCCTACCTGCAGGGCACGAGATTTTACATCAGTCCGGACGCCTTC
AacA_Y357_flank_R	CTCGTGCCCTGCAGGTAGGC
AacA_Y306F/R311K_F	CCCAACATTTTTGATCCCACTAAGCCTCGAGTCGACCCAGTCGTG
AacA_Y306F/R311K_flank_R	AGTGGGATCAAAAAATGTTGGG
AacA_F444Y_F	CGGCTTCCCTACAAGTTGGGTCTATAGATATGGATCGACCGGAGTGAAGATTGG
AacA_F444_Flank_R	CTATAGACCCAACTTGTAGGGAAGCCG
AacA_F444Y/T447K_F	GTTGGGTCTATAGATATGGATCGAAGGGAGTGAAGATTGGGAACAGG
AacA_F444Y/T447_flank_R	CGATCCATATCTATAGACCCAAC
AacA_R443H/F444Y/G445A_F	CTTCCCTACAAGTTGGGTCTATCACTATGCATCGACCGGAGTGAAGATTGGGAAC
AacA_R443_flank_R	ATAGACCCAACTTGTAGGGAAGCCG
**pXW55_*astC***	

AstC_xw55_F	catatggctagcgattataaggatgatgatgataagactagtATGACCAAGATCAACCCCTACAAGG
AstC_xw55_R	tcatttaaattagtgatggtgatggtgatgcacgtgAATTCCAGCGCACTGGTTGGCTTG
	**pXW55_*astC* mutant variants**
AstC_S35A_F	GCTCCCTATCAACACCTTCAAAGCTATTCTGTGCTGTGGTGCAACGGC
AstC_S35_flank_R	TTTGAAGGTGTTGATAGGGAGCTTGATCTGGTC
AstC_C38A_F	CCCTATCAACACCTTCAAATCTATTCTGGCCTGTGGTGCAACGGCTCAATATCAATG
AstC_C38_flank_R	CAGAATAGATTTGAAGGTGTTGATAGGGAGC
AstC_Y256F_F	GAATACCACCCAACTACATGGAACTTCTTCATTGGTACACCCGTACTCACAC
AstC_F257Y_F	GAATACCACCCAACTACATGGAACTACTACATTGGTACACCCGTACTCACACAG
AstC_Y256F/F257Y_F	GAATACCACCCAACTACATGGAACTTCTACATTGGTACACCCGTACTCACACAG
AstC_Y256_flank_R	AGTTCCATGTAGTTGGGTGGTATTCAAG
AstC_D271A_F	CACAGACAGAGTTTCCGCATGCTCTGGACACAACATCGCTTGCCAC
AstC_D271N_F	CACAGACAGAGTTTCCGCATAATCTGGACACAACATCGCTTGCCAC
AstC_D271_flank_R	ATGCGGAAACTCTGTCTGTGTGAGTAC
AstC_D273N_F	CAGAGTTTCCGCATGATCTGAACACAACATCGCTTGCCACG
AstC_D273_flank_R	CAGATCATGCGGAAACTCTG
AstC_F309Y_F	CGATGACCTCATGCTGACATACTTCACAGATTTTAAAAACCGTGTGGATCC
AstC_F309_flank_R	ATGTCAGCATGAGGTCATCGTCGC
AstC_K314A_F	CATGCTGACATTCTTCACAGATTTTGCAAACCGTGTGGATCCAGTTGTATGC
AstC_K314R_F	CATGCTGACATTCTTCACAGATTTTCGAAACCGTGTGGATCCAGTTGTATGC
AstC_K314_flank_R	AAAATCTGTGAAGAATGTCAGCATGAGGTC
AstC_R316A_F	GCTGACATTCTTCACAGATTTTAAAAACGCTGTGGATCCAGTTGTATGCTGCAATG
AstC_R316_flank_R	GTTTTTAAAATCTGTGAAGAATGTCAGCATGAGG
AstC_A359R_F	CGAGCATACATCAATGGGACTCGATTCTATCCTATGCCTGAAGCCTTTCTG
AstC_F360Y_F	CGAGCATACATCAATGGGACTGCATACTATCCTATGCCTGAAGCCTTTCTGTATTTC
AstC_A359R/F360Y_F	CGAGCATACATCAATGGGACTCGATACTATCCTATGCCTGAAGCCTTTCTGTATTTC
AstC_A359_flank_R	AGTCCCATTGATGTATGCTCGTCG
AstC_Y446F_F	GCTGGGATCTAGGAACGATGTTCCATTACGCATCTAAAAGGCTGCCG
AstC_Y446A_F	GCTGGGATCTAGGAACGATGGCCCATTACGCATCTAAAAGGCTGCCG
AstC_Y446_flank R	CATCGTTCCTAGATCCCAGCTGCC
AstC_H447A_F	GCTGGGATCTAGGAACGATGTACGCTTACGCATCTAAAAGGCTGCCGATCG

AstC_Y448A_F	GGATCTAGGAACGATGTACCATGCCGCATCTAAAAGGCTGCC GATCG
AstC_Y448A_flank_R	ATGGTACATCGTTCCTAGATCCCAGC
AstC_Y448F_F	GATCTAGGAACGATGTACCATTTCGCATCTAAAAGGCTGCCGA TC
AstC_Y448_flank_R	AATGGTACATCGTTCCTAGATCCCAGC
AstC_H447K/Y448F/A449P_F	CTGGGATCTAGGAACGATGTACAAGTTCCCATCTAAAAGGCTG CCGATCGGAAACC
AstC_H447R/Y448F/A449G_F	CTGGGATCTAGGAACGATGTACAGGTTCGGATCTAAAAGGCTG CCGATCGGAAACC
AstC_H447_flank_R	GTACATCGTTCCTAGATCCCAGCTGC
**pCold_*aacA***	
AacA_pCold_F	catcatcatcatatcgaaggtaggcatATGCCCTCCGTCAAAGCACTG
AacA_pCold_R	cagagattacctatctagactgcaggtcgacctaTCGAATGTCAGTCGTCAATC CCCAC
**pET30_*aacA*$_{aa196-466}$**	
AacA_aa196_pET30_F	ggattataaggatgatgatgataagactagtATGGGAGACCCTGTCTCTCGG GG
AacA_aa466_pET30_R	gtggtggtgatggtgatgctcgagCTCAATGGCCTTTATTGCAAGTGCAG
**pCold_*astC***	
AstC_pCold_F	catcatcatcatatcgaaggtaggcatATGACCAAGATCAACCCCTACAAG
AstC_pCold_R	cagagattacctatctagactgcaggtcgacAATTCCAGCGCACTGGTTGGC
**pCold_*astC* $_{aa5-475}$**	
AstC_aa5_pColdI_F	catcatcatcatatcgaaggtaggcatatgAACCCCTACAAGGGGATACTGG TTG
AstC_aa475_pColdI_R	gagattacctatctagactgcaggtcgacctaGTTGGCTTGACATTGCTTGATT GC
**pET30_*astC* $_{aa200-475}$**	
AstC_aa200_pET30_F	ggatgatgatgataagactagtATGGACCATATCTCCCGTGCGGAAC
AstC_aa475_pET30_R	ggtggtggtgatggtgatgctcgagGTTGGCTTGACATTGCTTGATTGC
**Others**	
pCold_3'_F	gtcgacctgcagtctagatagg
pCold_5'_R	atgcctaccttcgatatgatgatgatgatg
pET30_3'_F	ctcgagcatcaccatcaccaccacc
pET30_5'_R	catactagtcttatcatcatcatccttataatccatatgatgtatatctc
ori_3'_F	ctgcgcgtaatctgctgcttgcaaac
ori_5'_R	gtttgcaagcagcagattacgcgcag
ADH2P_3’_R	gatggtattacgatatagttaatagttgatagttgattg
ADH2T_5’_F	cacgtgcatcaccatcaccatcactaatttaaatg
URA3_3'_F	ctaggttcctttgttacttcttctgccg
URA3_5'_R	cggcagaagaagtaacaaaggaacctag

Table S4. Mass spectrometric protein identification of AacA and AstC variants.

Uniport code	Description [a]	Sun PEP Score	Coverage %	PSMs	Unique Peptides	AAs	MW (kDa)	Gene Symbol	Source	Detected in samples
	sample 1. **AacA-TC** (Gly^{196}–Glu^{466}) [b]	158.458	61	97	29	295	32.8	*aacA-TC*	*A. aculeatus*	1
	sample 2. **AacA_D268A** [c]	341.964	81	444	55	499	56.5	*aacA_D268A*	*A. aculeatus*	2
	sample 3. **AacA_D268N** [c]	301.917	75	362	52	499	56.5	*aacA_D268N*	*A. aculeatus*	3
	sample 4. **AstC-TC** (Asp^{200}–Asn^{475}) [b]	294.694	49	464	41	502	33.8	*aacA-TC*	*A. oryzae*	4
	sample 5. **AstC_D271A** [c]	302.386	66	241	45	502	55.6	*astC_D271A*	*A. oryzae*	5
	sample 6. **AstC_D271N** [c]	275.133	69	269	45	502	55.6	*astC_D271N*	*A. oryzae*	6
**A1AJ51**	Chaperonin GroEL 1 [e]	662.827	99	1261	84	548	57.3	*groEL1*	*E. coli*	1 – 6
**A7ZHA4**	Chaperone protein DnaK	290.84	79	93	47	638	69.1	*dnaK*	*E. coli*	1, 3, 4, 5, 6
**P0ABB9**	Magnesium-transporting ATPase, P-type 1	248.501	51	78	52	898	99.4	*mgtA*	*E. coli*	1, 4
**A7ZUE6**	ATP-dependent protease ATPase subunit HslU [e]	136.375	58	38	29	443	49.6	*hslU*	*E. coli*	1 – 6
**A7ZTY0**	Guanosine-5'-triphosphate,3'-diphosphate pyrophosphatase	78.669	41	22	17	494	54.9	*gppA*	*E. coli*	1 – 6
**P37617**	Zinc/cadmium/lead-transporting P-type ATPase	45.192	21	11	9	732	76.8	*zntA*	*E. coli*	1 – 6
**P0AF25**	Ribonucleotide monophosphatase NagD [d]	35.838	50	12	9	250	27.1	*nagD*	*E. coli*	1, 4
**A7ZV11**	Co-chaperonin GroES	23.495	69	7	6	97	10.4	*groES*	*E. coli*	1, 4
**A7ZKD6**	Probable phosphatase YcdX	19.151	30	4	4	245	26.8	*ycdX*	*E. coli*	1, 4
**P0A7B0**	Inorganic pyrophosphatase	1.801	6	2	1	176	19.7	*ppa*	*E. coli*	1

[a] All data was filtered at 1% false discovery rate (FDR) at Peptide.
[b] Sample preparation for sample 1 and 4 (SDS-PAGE shown in Figure S3 and S10, respectively) are using in-solution digestion.
[c] Sample preparation for sample 2, 3, 5, and 6 are using in-gel digestion of excised band at ~50 kDa (SDS-PAGE shown in Figure S3 and S10).
[d] NagD[19] is a characterized *E. coli* ribonucleotide monophosphatase that belongs to the HAD-like enzyme family.
[e] GroEL[20] and HslU[21] are two major *E. coli* proteins bearing phosphatase-like activity detected in all samples.
Quantitative values of *E. coli* endogenous proteins are abstracted from sample 1. **AacA-TC** (Gly^{196}–Glu^{466}).

Table S5. Mass spectrometric protein identification of major co-purified *E. coli* host proteins in AacA-TC and AstC-TC.

Uniport code	Description	Sum PEP Score		Coverage (%)		Numbers of Peptides		PSMs		Unique Peptides		AAs	MW (kDa)	Gene symbol
		I[a]	II[a]	I	II	I	II	I	II	I	II			
**A1AJ51**	Chaperonin GroEL 1	115.83	171.428	74	78	27	36	54	83	27	36	548	57.3	groEL1
**A7ZSK6**	Large ribosomal subunit protein uL2	66.874	104.779	63	72	20	31	58	65	20	31	273	29.8	rplB
**C4ZU97**	Bifunctional polymyxin resistance protein ArnA	51.75	53.33	29	32	16	17	18	19	2	17	660	74.3	arnA
**P0A9R9**	Cell division ATP-binding protein FtsE	45.464	50.862	82	79	14	14	23	18	14	14	222	24.4	ftsE
**P0AF25**	Ribonucleotide monophosphatase NagD	30.921	13.357	56	30	9	5	11	5	9	5	250	27.1	nagD
**A7ZUJ7**	Large ribosomal subunit protein	30.699	26.453	56	41	10	7	13	7	10	7	234	24.7	rplA
**A7ZHA4**	Chaperone protein DnaK	18.983	50.823	17	26	6	16	8	20	6	16	638	69.1	dnaK
**A7ZUE6**	ATP-dependent protease ATPase subunit HslU	7.18	24.149	5	24	2	8	2	8	2	8	443	49.6	hslU

[a] Samples I and II denote in-gel digestion followed by proteomic analysis of co-purified *E. coli* host protein bands (~25 kDa), excised from AacA-TC and AstC-TC samples, respectively. The corresponding bands are indicated by purple arrow in Figures S3 and S10.
[b] List of *E. coli* host proteins identified from the excised bands that are associated with chaperone or phosphatase-related functions.

Supplementary Figures

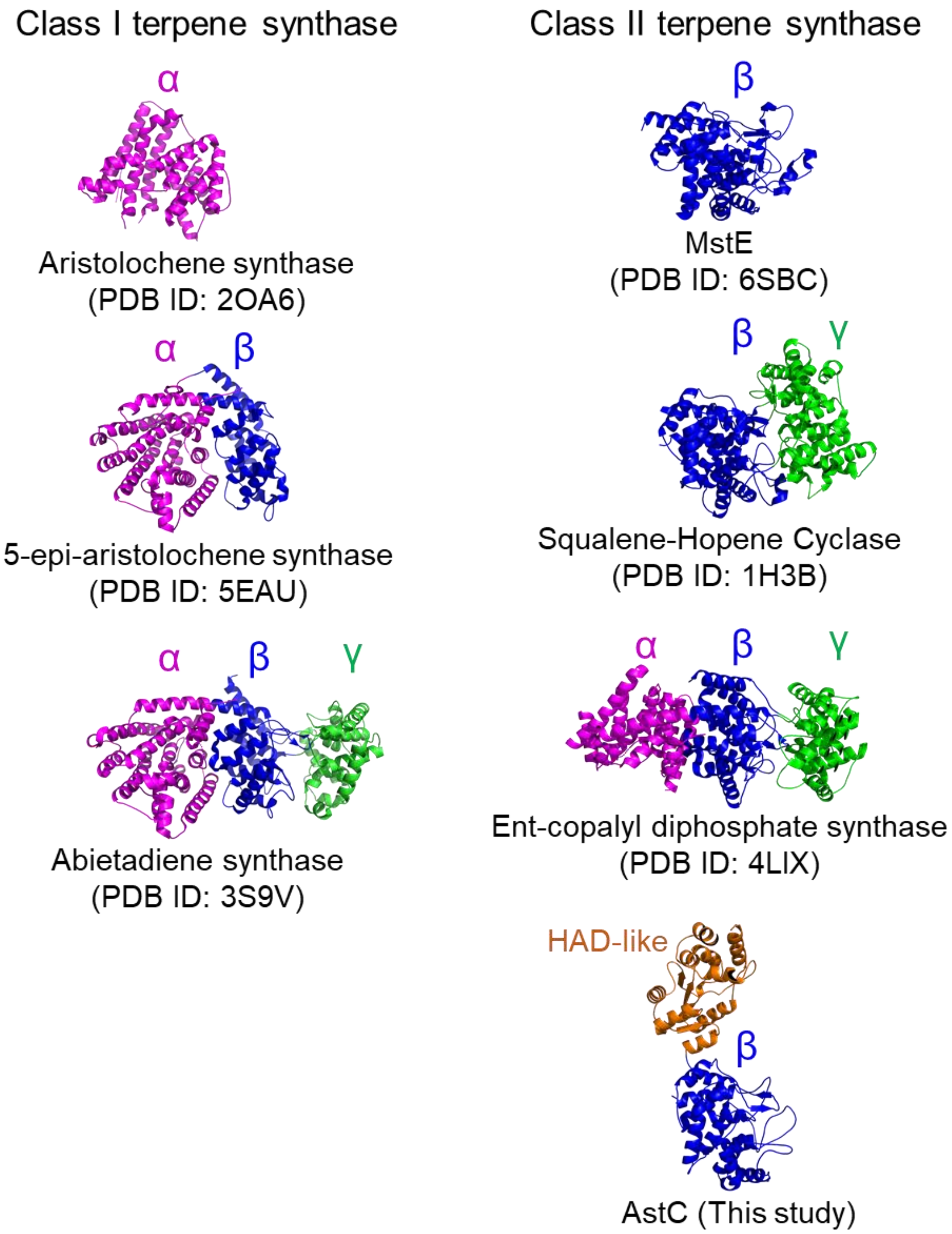

Figure S1. Overview of the architectures of class I and class II terpene cyclases.

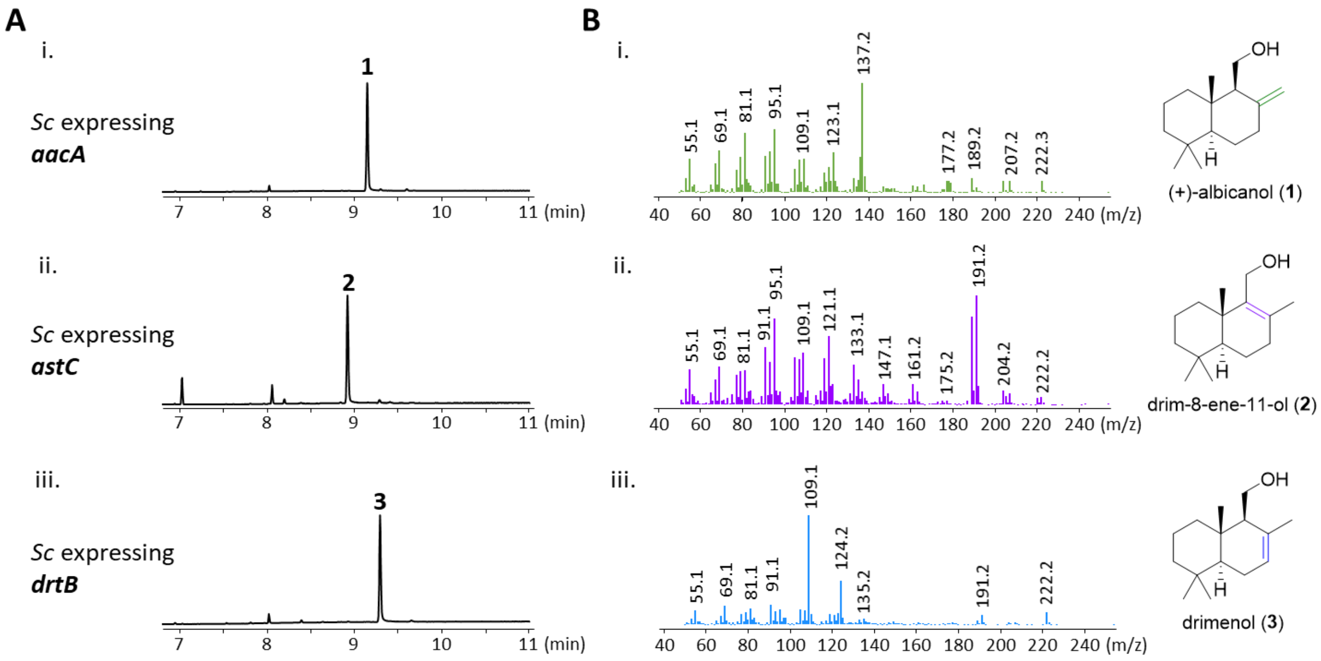

Figure S2. GC–MS analysis of fungal HAD-like terpene cyclase homologs expressed in *S. cerevisiae*. (A) GC chromatograms of yeast strains expressing *aacA*, *astC*, or *drtB*. (B) EI–MS spectra of the corresponding sesquiterpene products: (+)-albicanol (**1**), drim-8-ene-11-ol (**2**), and drimenol (**3**).

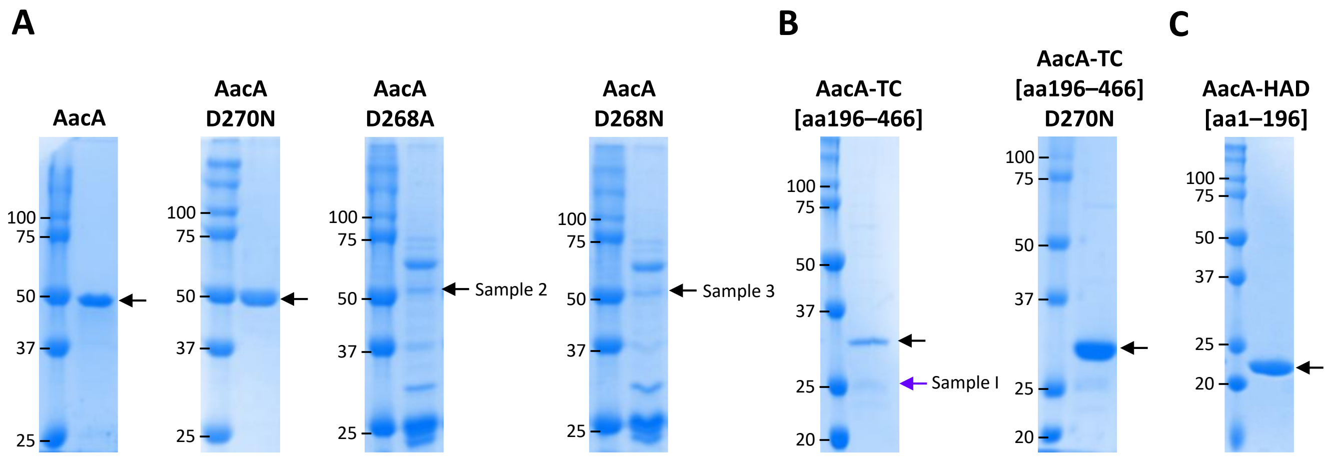

Figure S3. SDS-PAGE of purified recombinant proteins AacA and its variants. (A) Two-domain AacA (residues Met^{1}–Glu^{466}) and its mutant variants (calculated molecular weight 56.5 kDa). (B) AacA-TC domain (residues Gly^{196}–Glu^{466}) and its D270N mutant variant (32.8 kDa). (C) AacA-HAD domain (residues Met^{1}–Gly^{196}, 24.0 kDa). Note that all proteins carry an N-terminal 6×His tag. The AacA-D268A and AacA-D268N proteins were confirmed by protein identification (samples 2 and 3, respectively, in Table S4). The purple arrow indicates a co-purified *E. coli* host protein band, which was excised and subjected to in-gel digestion followed by proteomic analysis (sample I in Table S5).

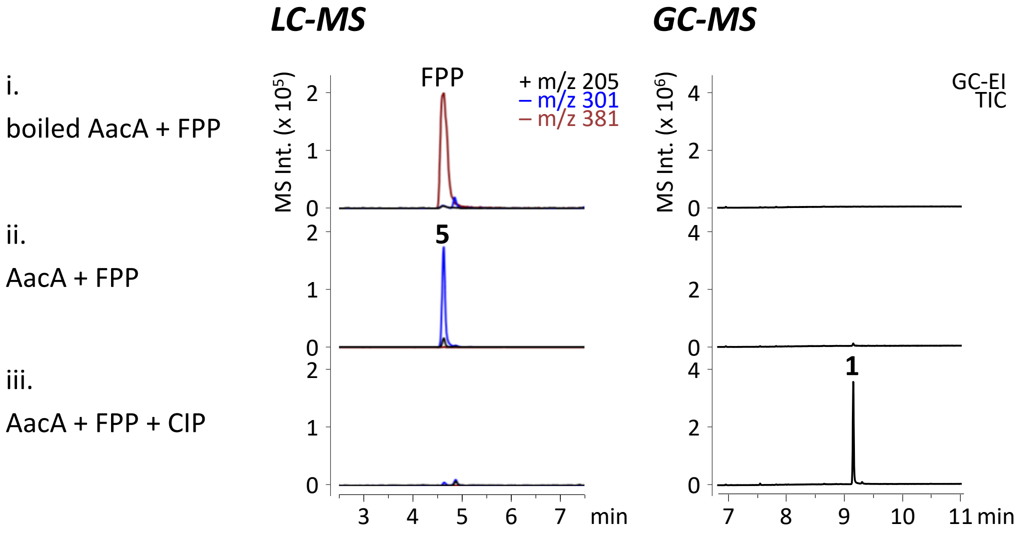

Figure S4. LC–MS and GC–MS analysis of *in vitro* FPP conversion by AacA. Reaction mixtures contained 1 µM AacA and 100 µM FPP in the buffer described in Materials and Methods and were incubated at ambient temperature for 30 min. Calf intestinal alkaline phosphatase (CIP; 2 units) was added after the 30 min incubation.

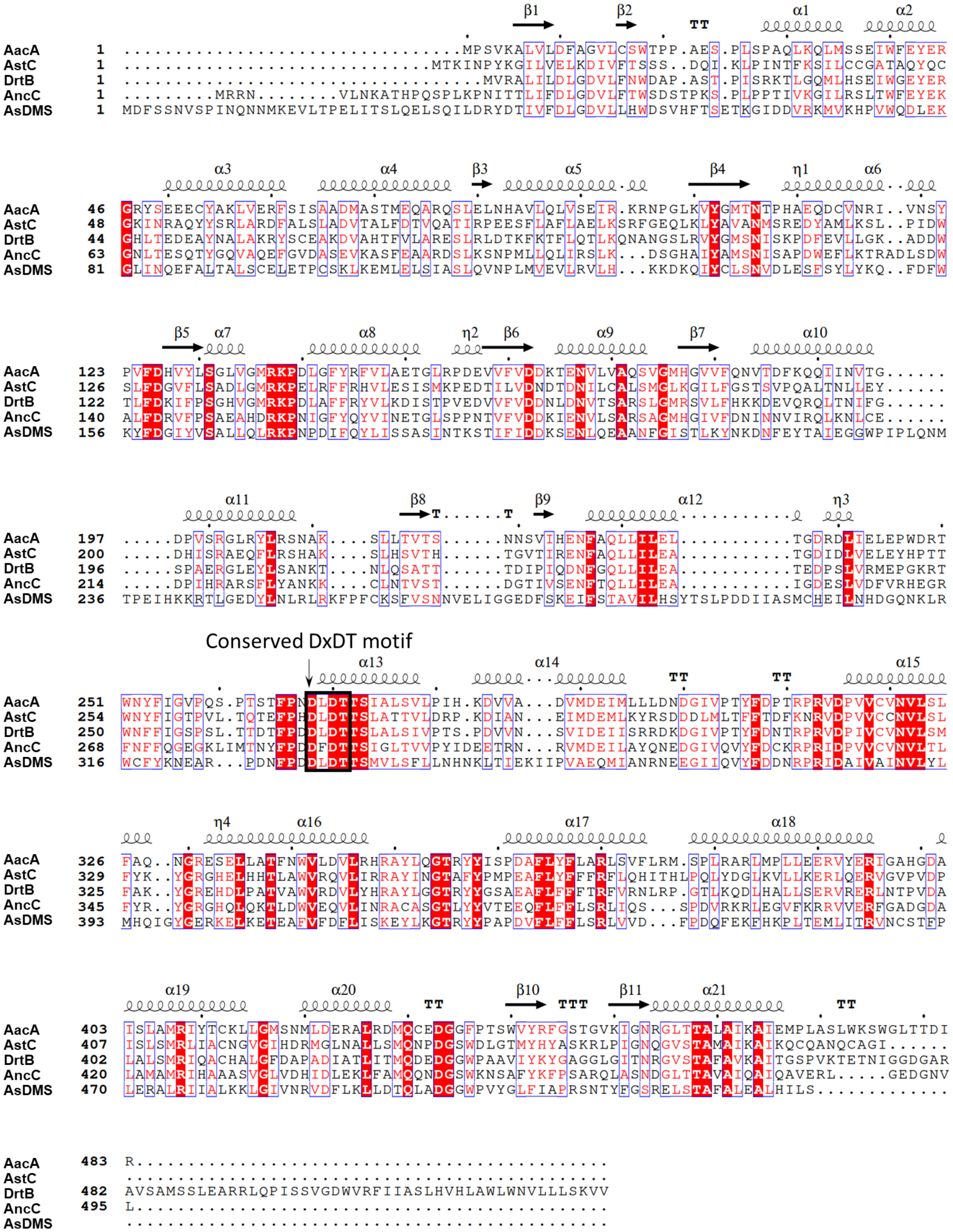

Figure S5. Amino acid sequence alignment of HAD-like TCs.

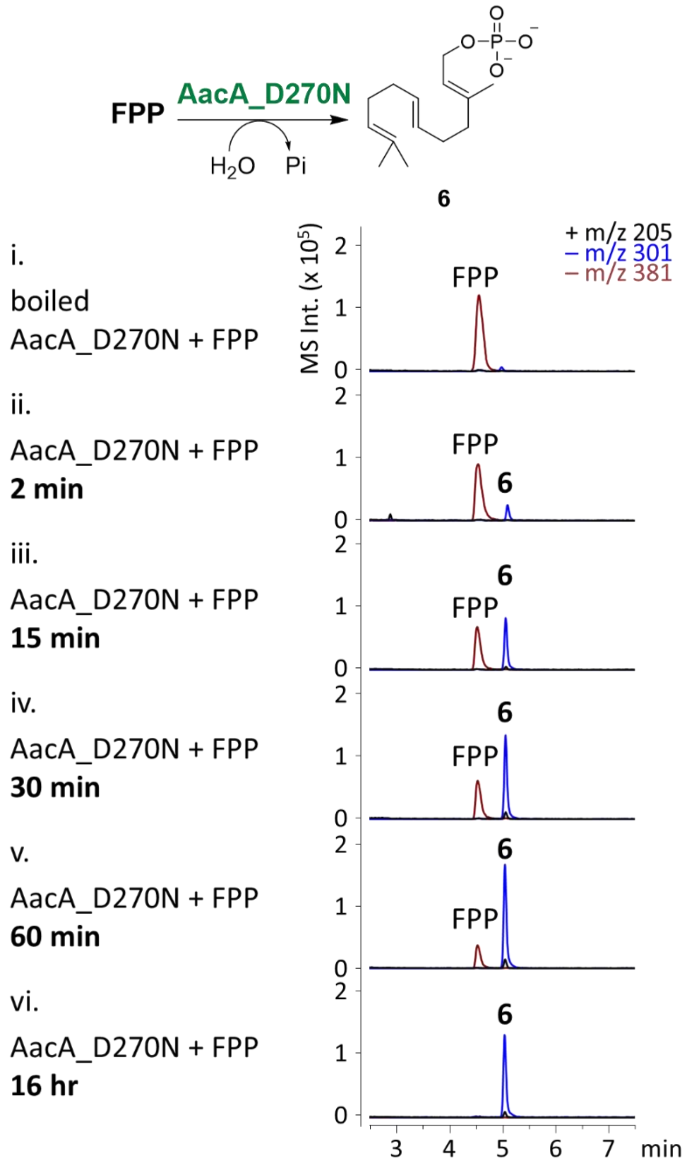

Figure S6. LC–MS analysis of *in vitro* FPP conversion by AacA_D270N over a 2 min–16 hr time course.

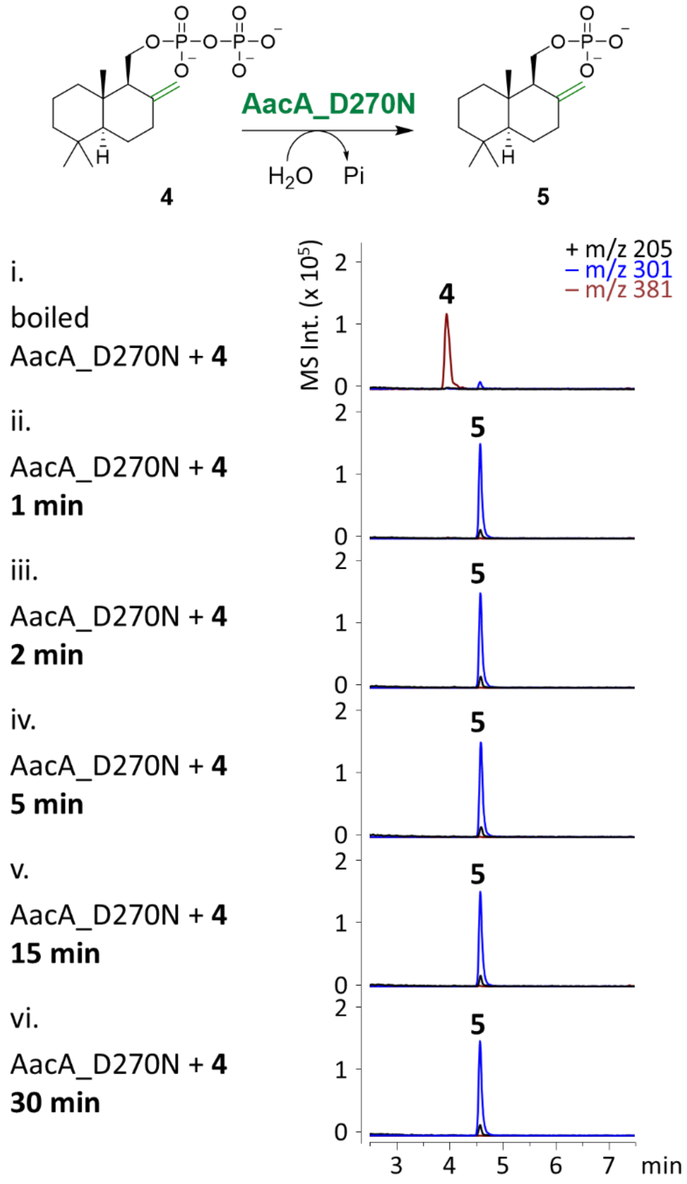

Figure S7. LC–MS analysis of *in vitro* compound **4** conversion by AacA_D270N over a 1–30 min time course.

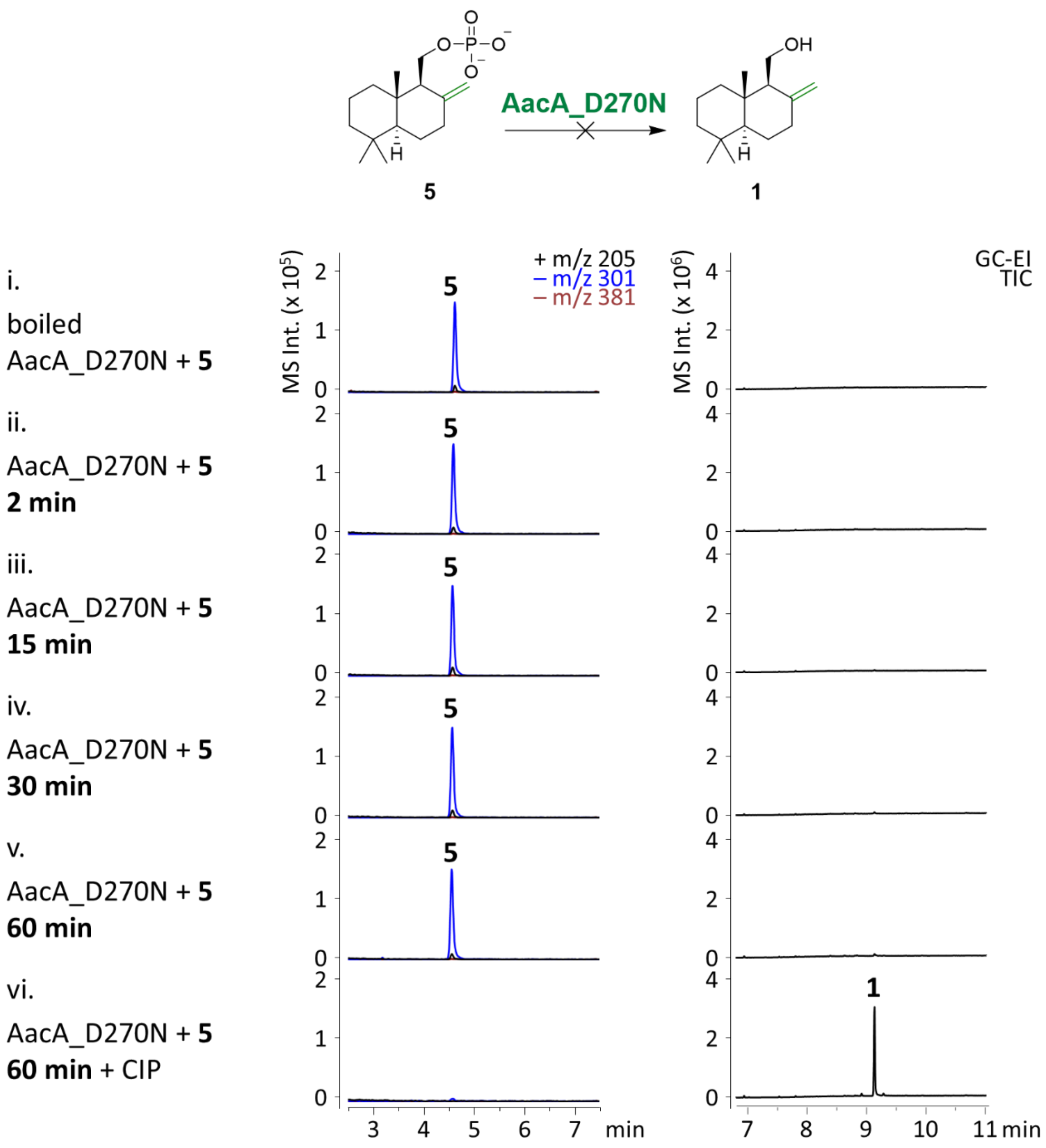

Figure S8. LC–MS and GC–MS analysis of *in vitro* assays of **5** with AacA_D270N over a 2–60 min time course.

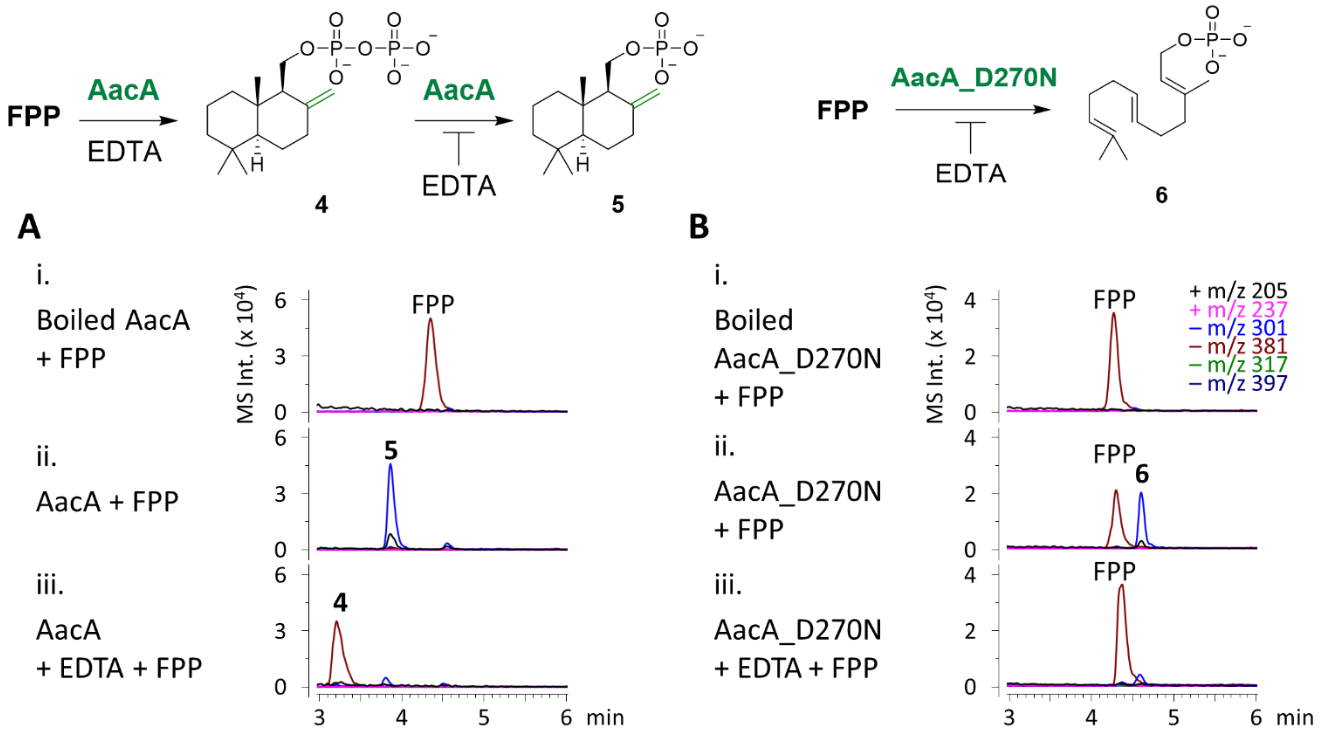

Figure S9. LC-MS analysis of EDTA effects on (A) AacA-catalyzed FPP conversion; (B) AacA_D270N-catalyzed FPP conversion.

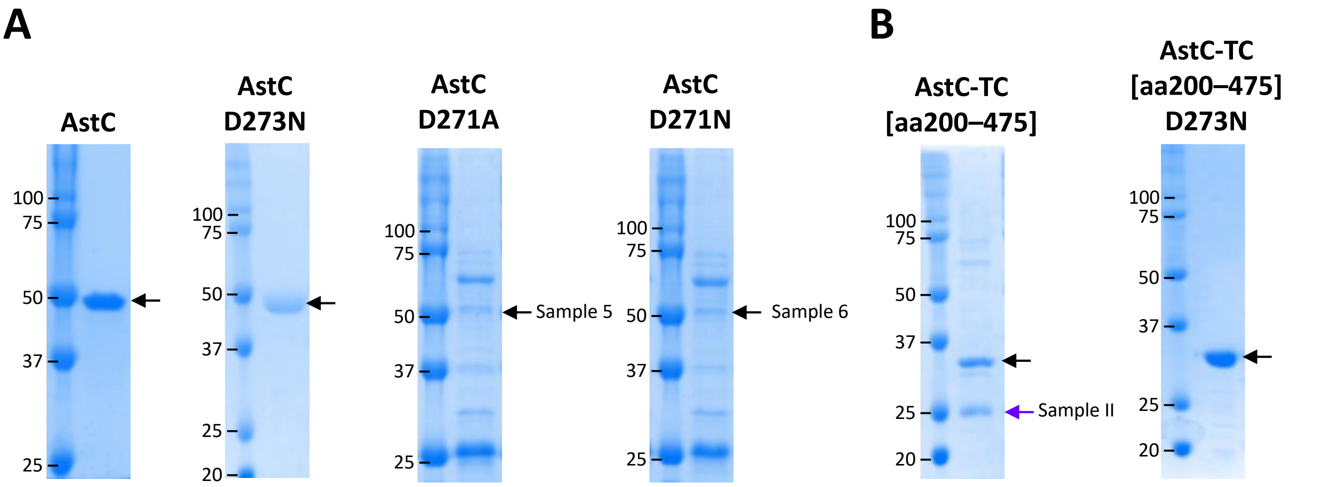

Figure S10. SDS-PAGE of purified recombinant AstC and its variants. (A) Two-domain AstC (residues Ile^{4}–Asn^{475}) and its mutant variants (calculated molecular weight 55.6 kDa). (B) AstC-TC single domain (residues Asp^{200}–Asn^{475}) and the D273N mutant variant (33.8 kDa). Note that all proteins carry an N-terminal 6×His tag. The AstC-D271A and AstC-D271N proteins were confirmed by protein identification (samples 5 and 6, respectively, in Table S4). The purple arrow indicates a co-purified *E. coli* host protein band, which was excised and subjected to in-gel digestion followed by proteomic analysis (sample II in Table S5).

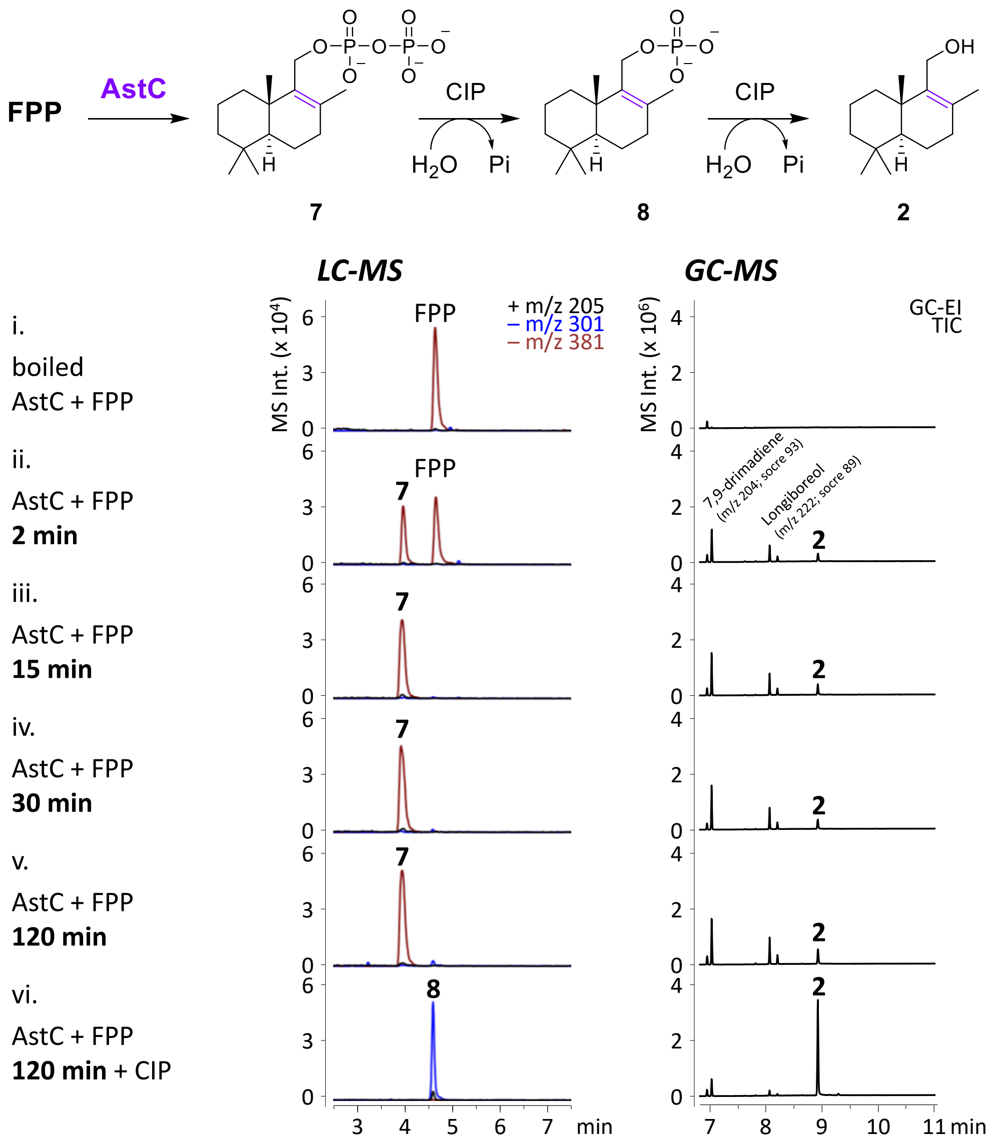

Figure S11. LC–MS and GC–MS analysis of *in vitro* assays of FPP with AstC over a 2–120 min time course. Additional peaks observed in the GC chromatograms at t_R 7.1 min (*m/z* 204) and t_R 8.1 min (*m/z* 222) are thermally decomposed products derived from the reaction intermediates.

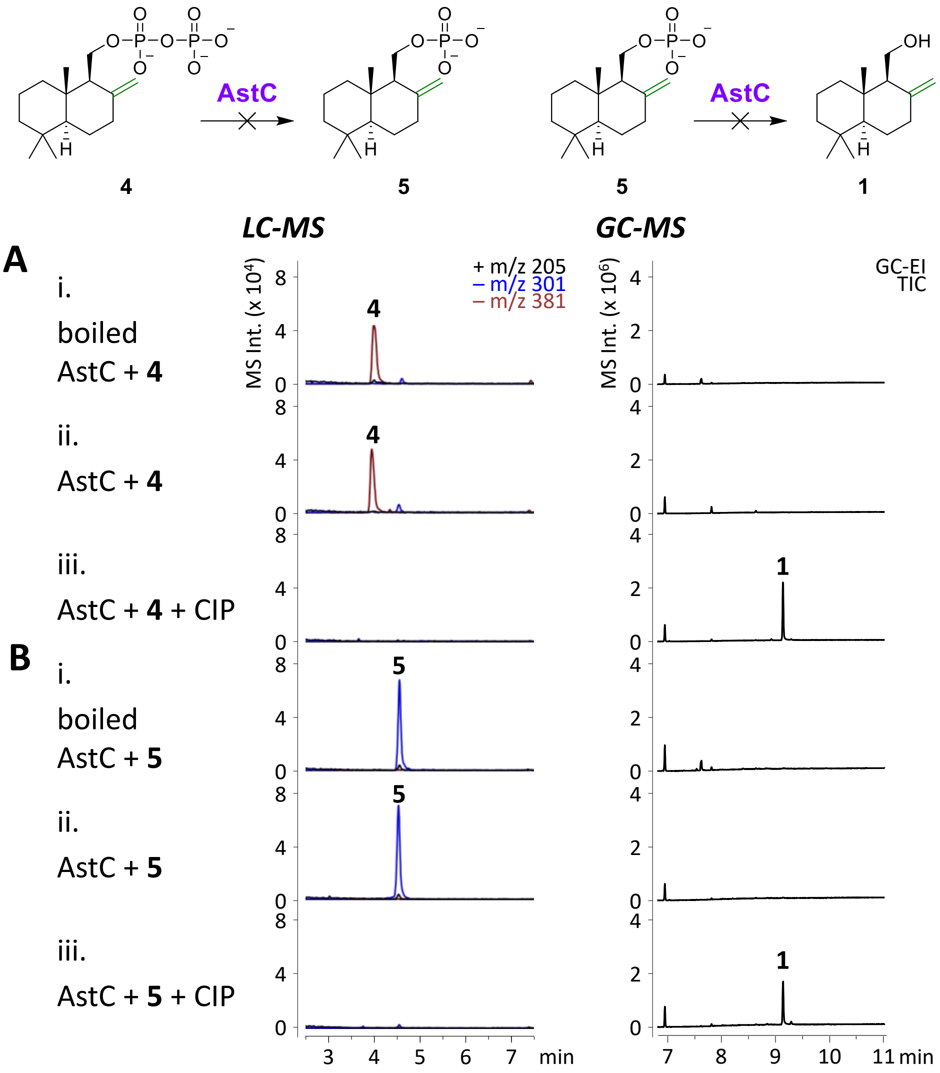

Figure S12. LC–MS and GC–MS analysis of *in vitro* assays of (A) **4**, or (B) **5** with AstC. Reaction mixtures contained 1 μM AstC and 100 μM substrate and were incubated at ambient temperature for 30 min. CIP (2 units) was added after the 30 min incubation. Reactions were quenched with acetonitrile and analyzed by LC–ESI–MS (left panels). The same reactions were also directly analyzed by SPME–GC–EI–MS (right panels).

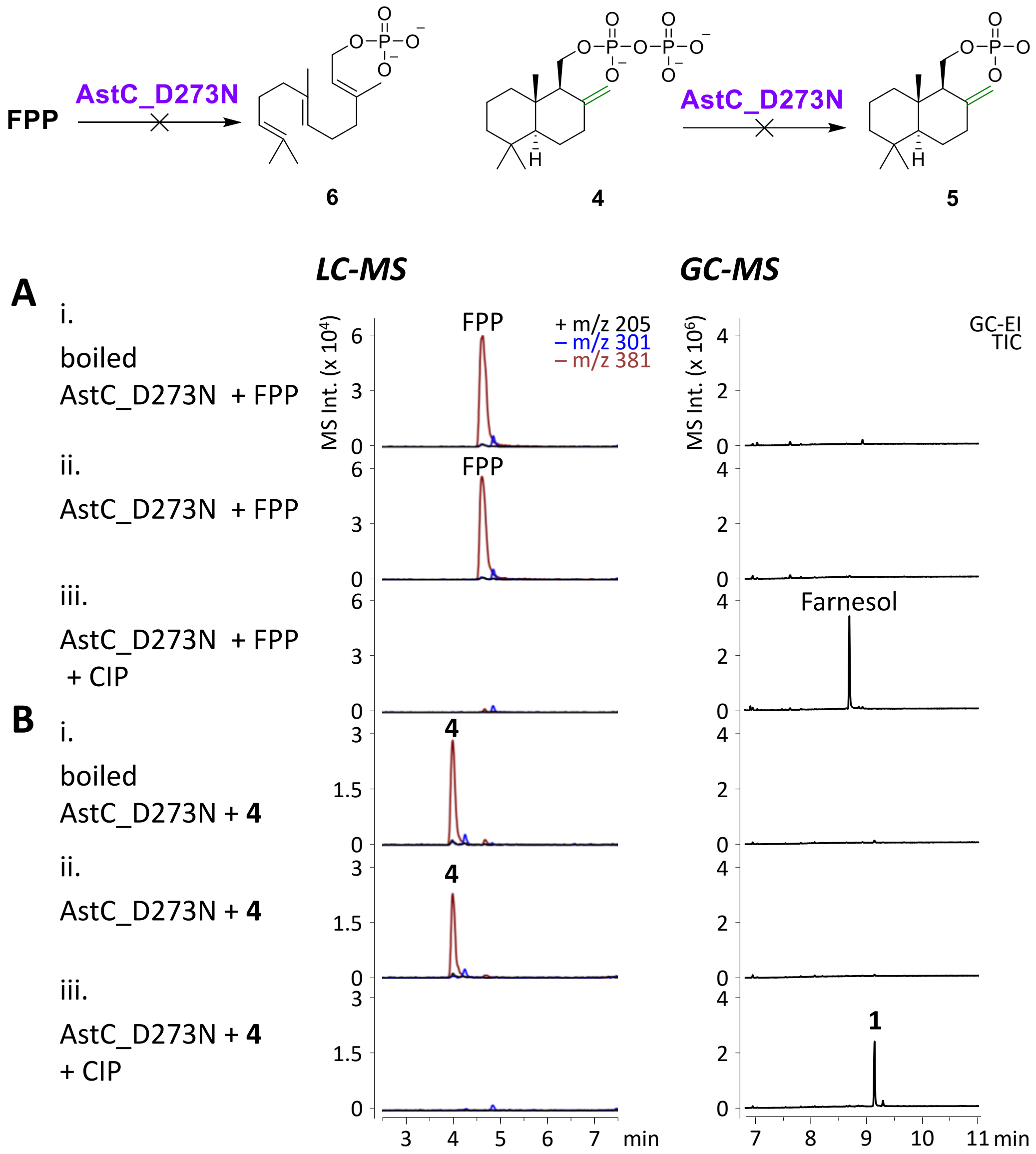

Figure S13. LC–MS and GC–MS analysis of *in vitro* assays of (A) FPP, or (B) **4** with AstC_D273N. Reaction mixtures contained 1 μM AstC_D273N and 100 μM substrate and were incubated at ambient temperature for 30 min. CIP (2 units) was added after the 30 min incubation. Reactions were quenched with acetonitrile and analyzed by LC–ESI–MS (left panels). The same reactions were also directly analyzed by SPME–GC–EI–MS (right panels).

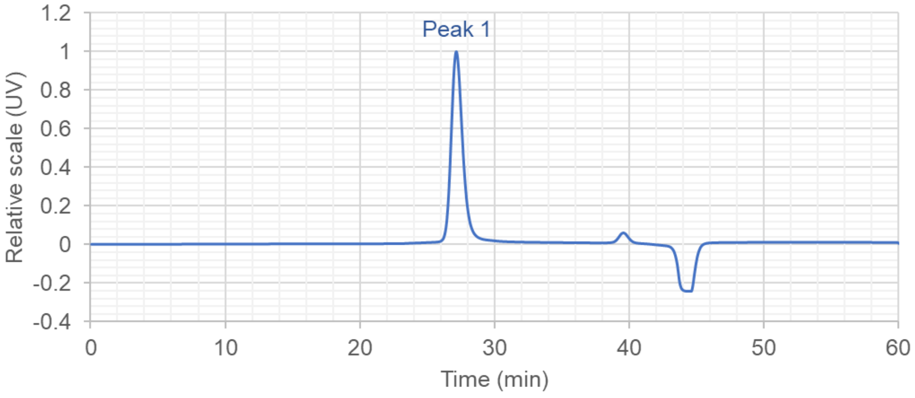

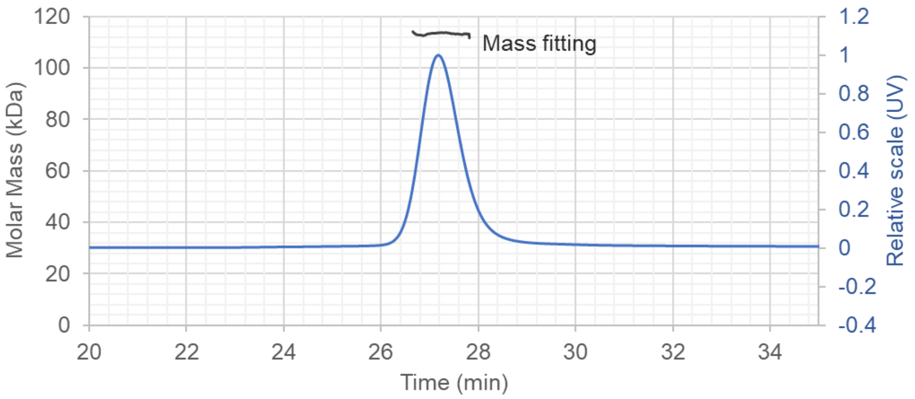

	Peak 1
Masses	
Injected Mass (μg)	100.00
Calculated Mass (μg)	72.37
Mass recovery (%)	72.4
Mass fraction (%)	100.0
Molar mass moments (g/mol)	
Mn	1.134×10^5 (±1.889%)
Mp	1.137×10^5 (±2.145%)
Mw	1.134×10^5 (±1.888%)
Mz	1.134×10^5 (±4.220%)
M(avg)	**1.132×10^5** (±0.047%)

Figure S14. SEC–MALS analysis of AstC_D273N. AacA_D273N (residues Ile^4–Asn^{475}) carries the catalytic inactivating mutation D273N and has a calculated monomer mass of 55.6 kDa. SEC–MALS measured an average molar mass of 1.132×10^5 Da for peak 1, consistent with a dimeric assembly in solution.

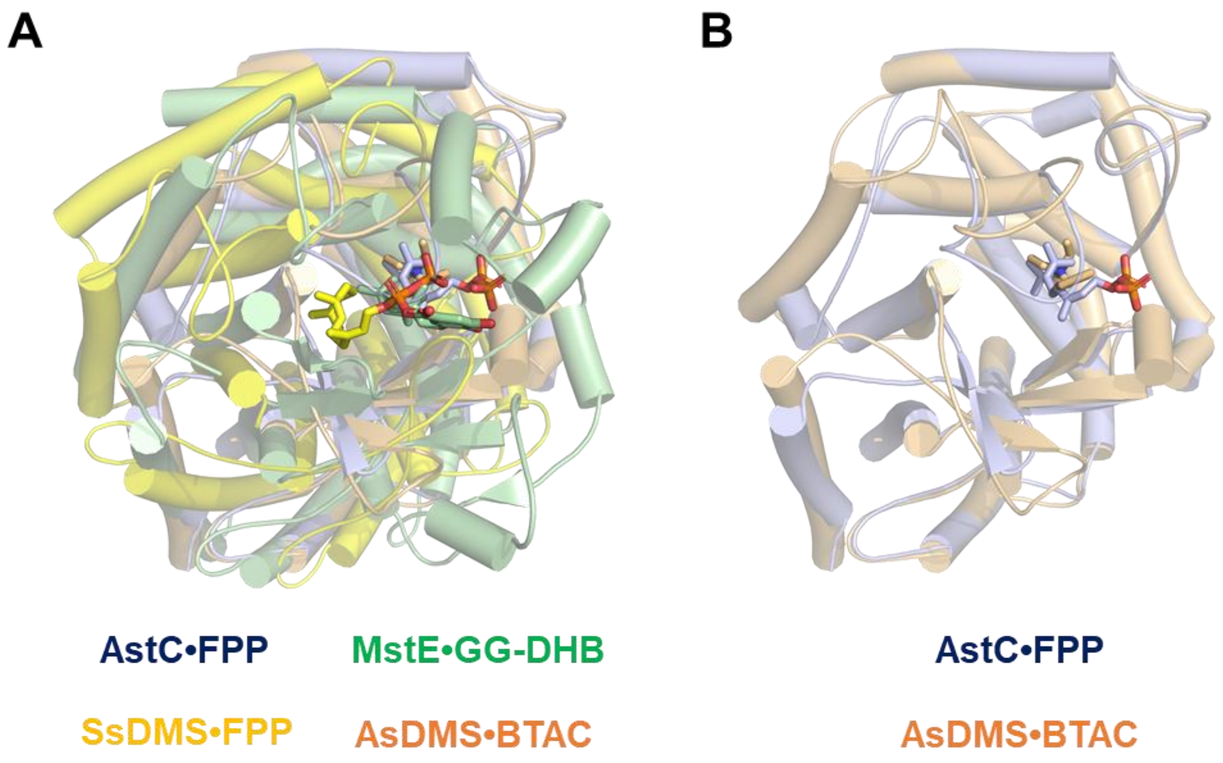

Figure S15. Structural comparison of AstC with other class II terpene cyclases. (A) Superposition of the TC domain of AstC•FPP with MstE•GG-DHB (PDB 6SBC), SsDMS•FPP (PDB 7XRA), and AsDMS•BTAC (PDB 9MHS), and (B) with AsDMS•BTAC (PDB 9MHS).

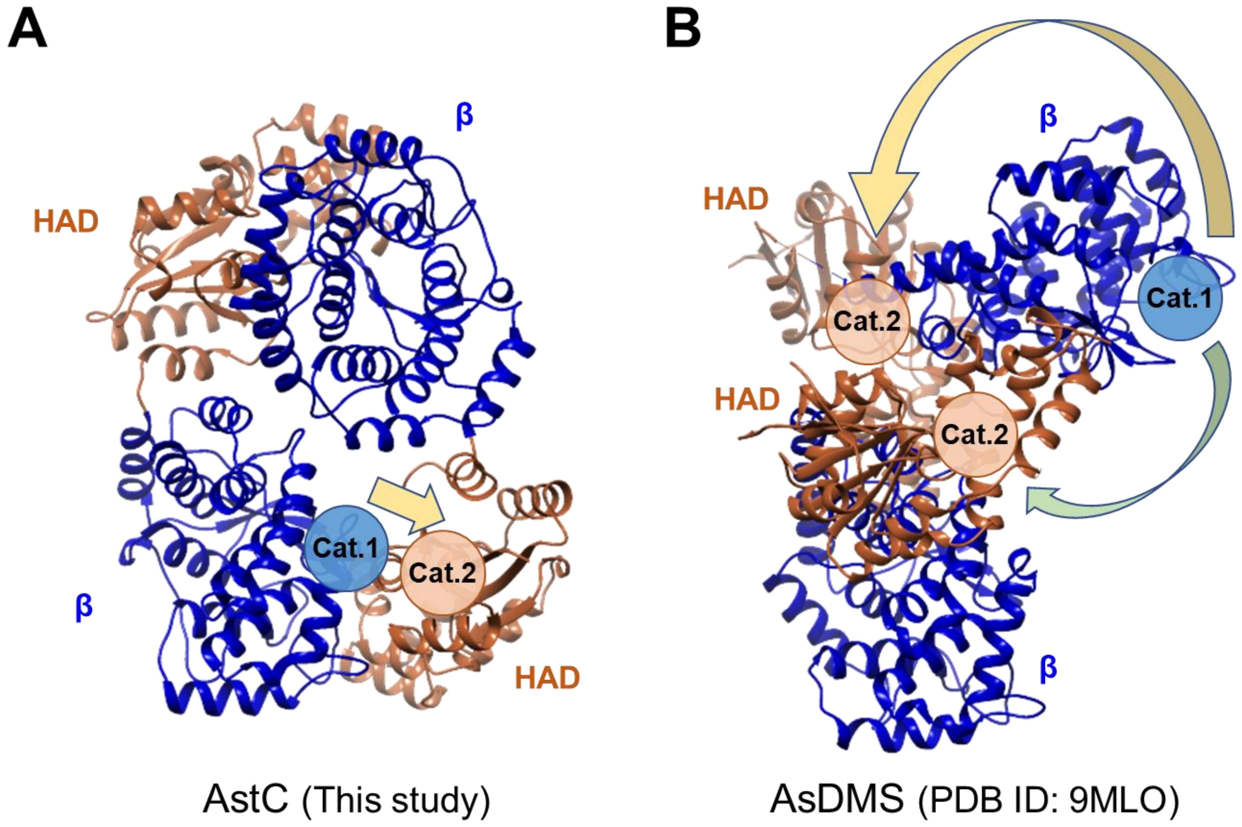

Figure S16. Comparison of the dimeric architectures of AstC and AsDMS. (A) AstC dimer highlighting the close proximity between catalytic site 1 in the TC domain (chain A; blue circle) and catalytic site 2 in the HAD-like domain (chain B; brown circle). (B) AsDMS dimer showing larger inter- and intraprotomer separations between sites 1 and 2, consistent with intermediate transfer via bulk-solvent diffusion, which is promoted by the electrostatic environment with positively charged interface between the TC and HAD domains.

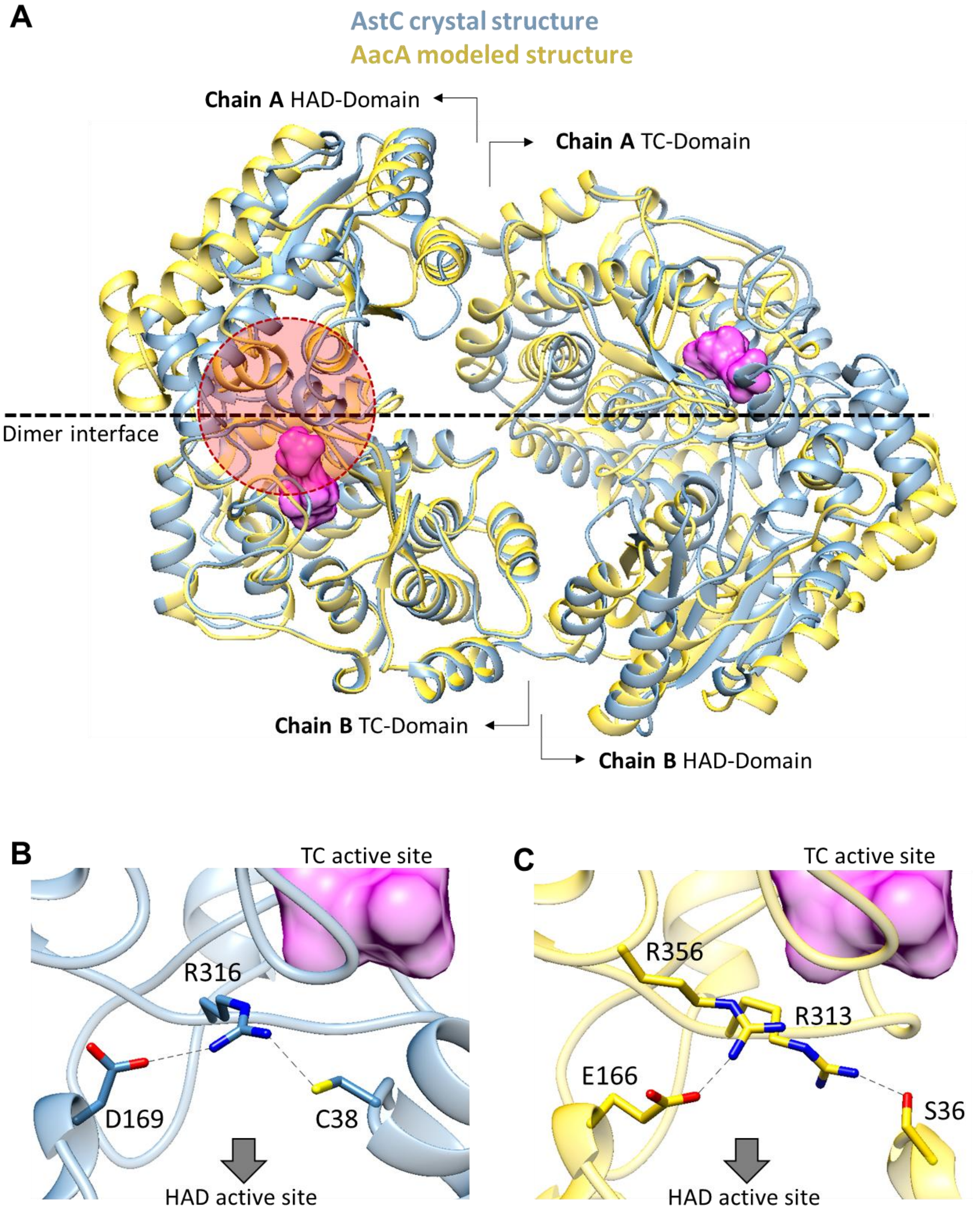

Figure S17. Structural superposition of the AstC crystal structure and an AlphaFold2-predicted AacA model. (A) Overlay of the dimeric assemblies of AstC and AacA. Salt-bridge residues at the TC–HAD domain interface are shown for (B) AstC and (C) AacA. Pink spheres indicate the position of FPP, based on the AstC•FPP complex structure.

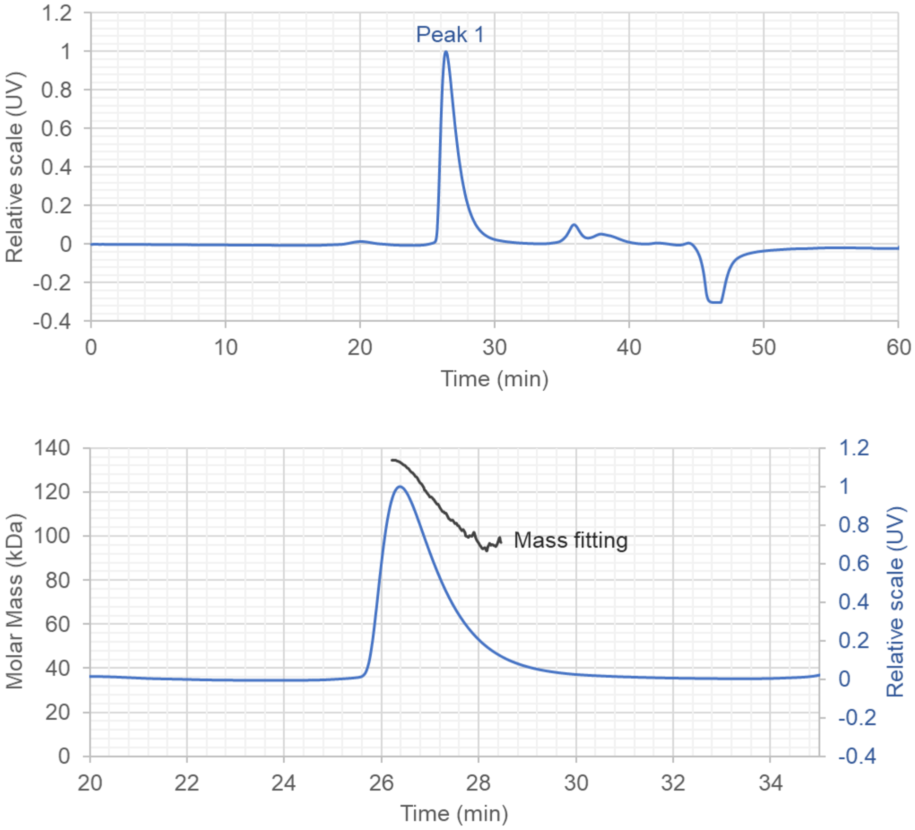

	Peak 1
Masses	
Injected Mass (µg)	100.00
Calculated Mass (µg)	79.94
Mass recovery (%)	79.9
Mass fraction (%)	97.9
Molar mass moments (g/mol)	
Mn	1.121×10^5 (±2.971%)
Mp	1.208×10^5 (±1.338%)
Mw	1.124×10^5 (±2.863%)
Mz	1.126×10^5 (±6.309%)
M(avg)	**1.143×10^5** (±0.243%)

Figure S18. SEC–MALS analysis of AacA_D270N. AacA_D270N (Met^1–Glu^{466}) carries the catalytic inactivating mutation D270N and has a calculated monomer mass of 56.5 kDa. SEC–MALS measured an average molar mass of 1.143×10^5 Da for peak 1, consistent with a dimeric assembly in solution.

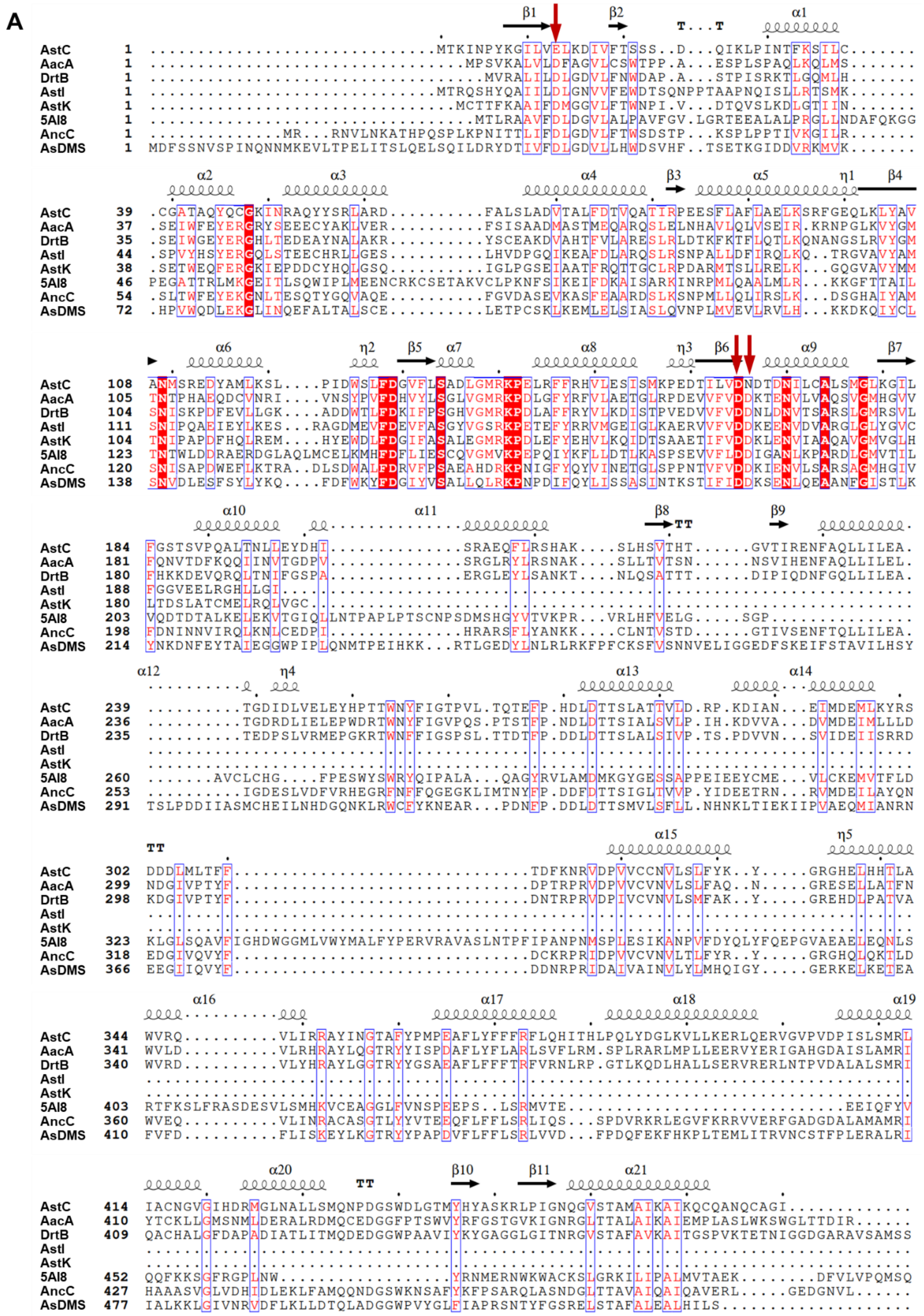

Figure S19. Amino acid sequence alignment of HAD-like enzymes. Red arrows indicate conserved residues implicated in Mg^{2+} coordination.

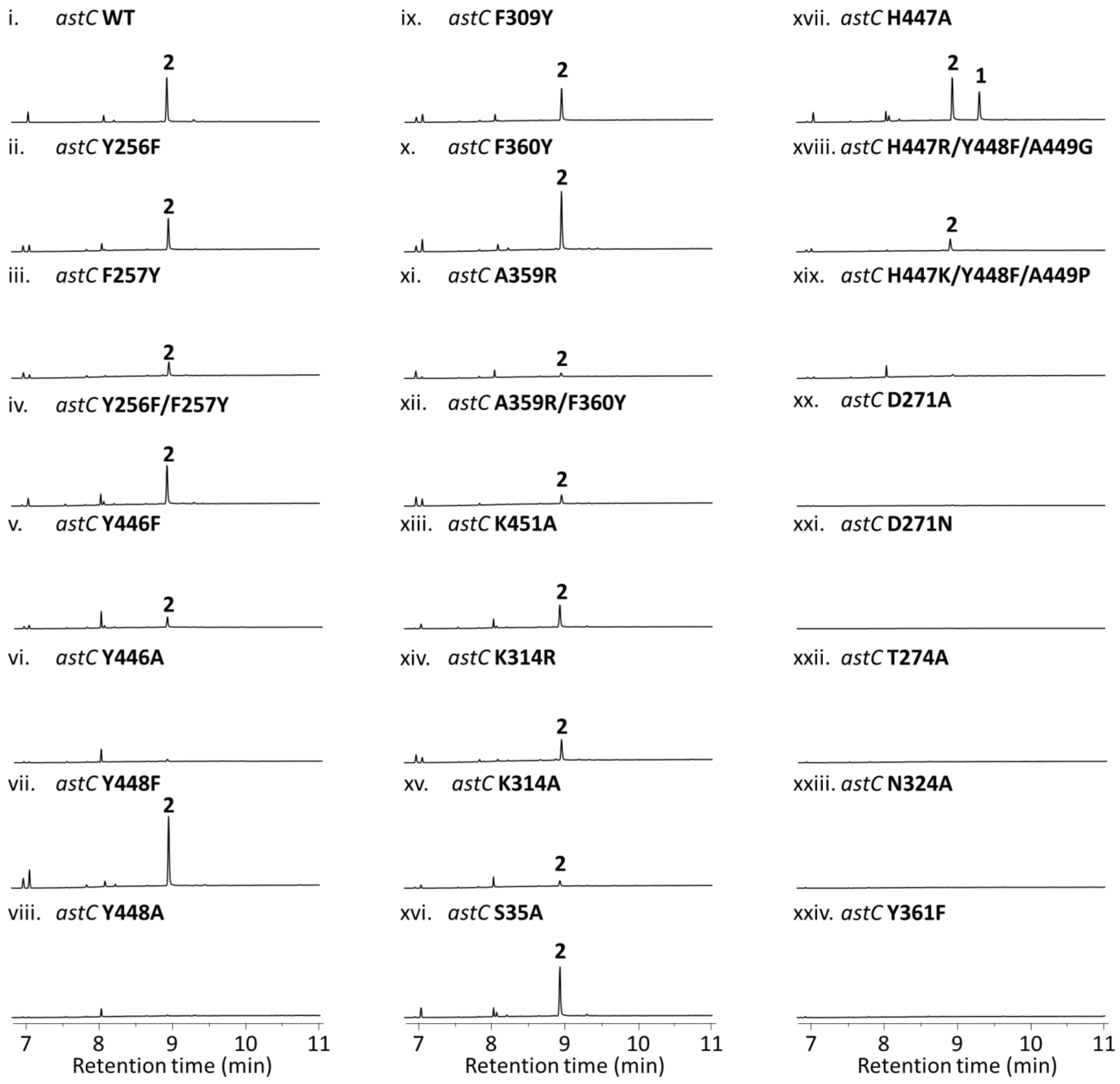

Figure S20. GC-MS of AstC mutants expressed in *S. cerevisiae*. The peak areas corresponding to drimanyl products were quantified and summarized in Figure 4G.

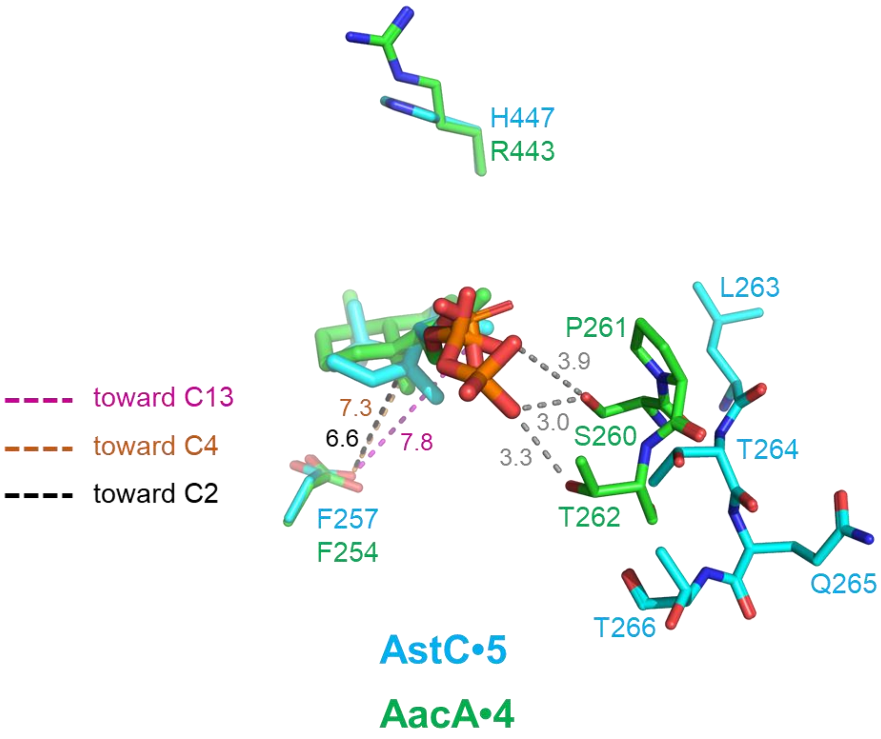

Figure S21. Superposition of the AstC-TC•**5** structure with an AlphaFold2-predicted AacA model docked with **4**, showing Ser^{260}–Thr^{262} as contributors to pyrophosphate binding.

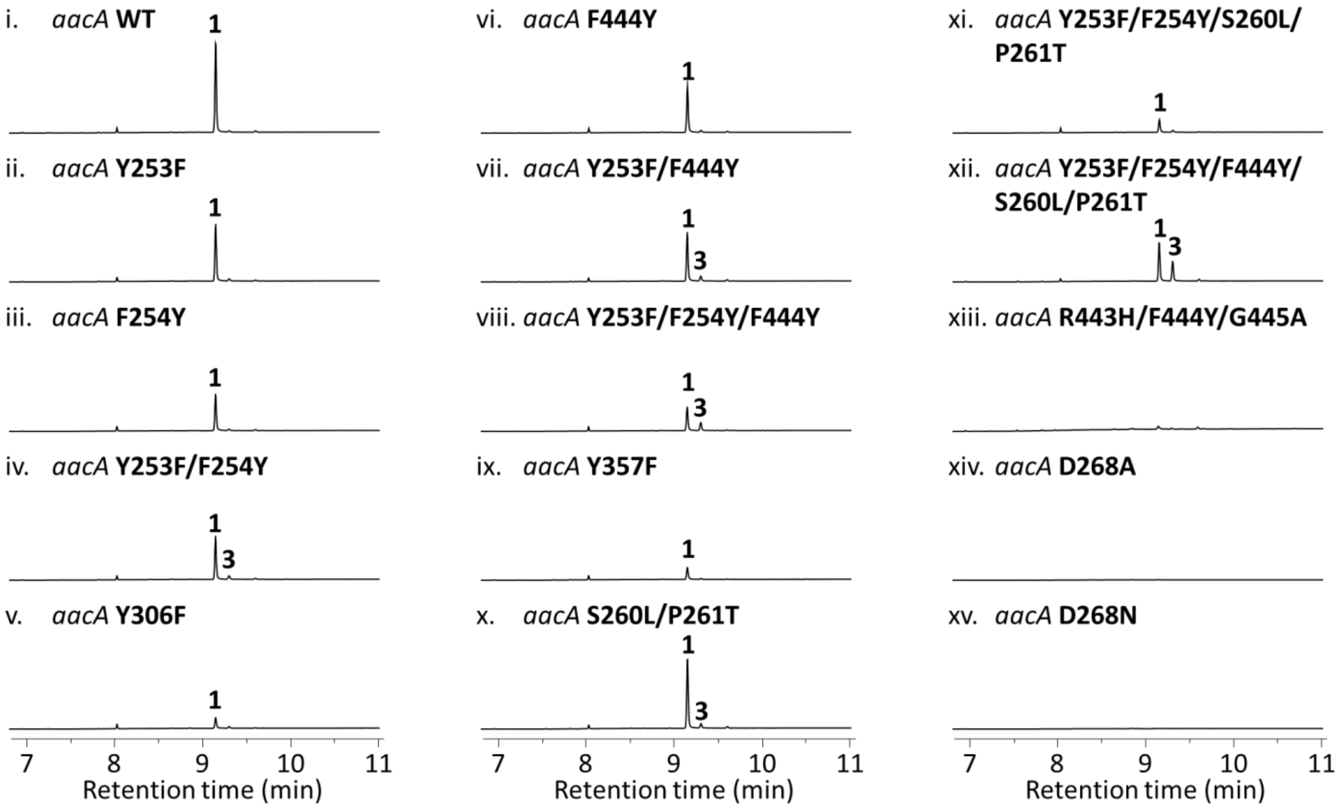

Figure S22. GC-MS of AacA mutants expressed in *S. cerevisiae*. The peak areas corresponding to drimanyl products were quantified and summarized in Figure 4H.

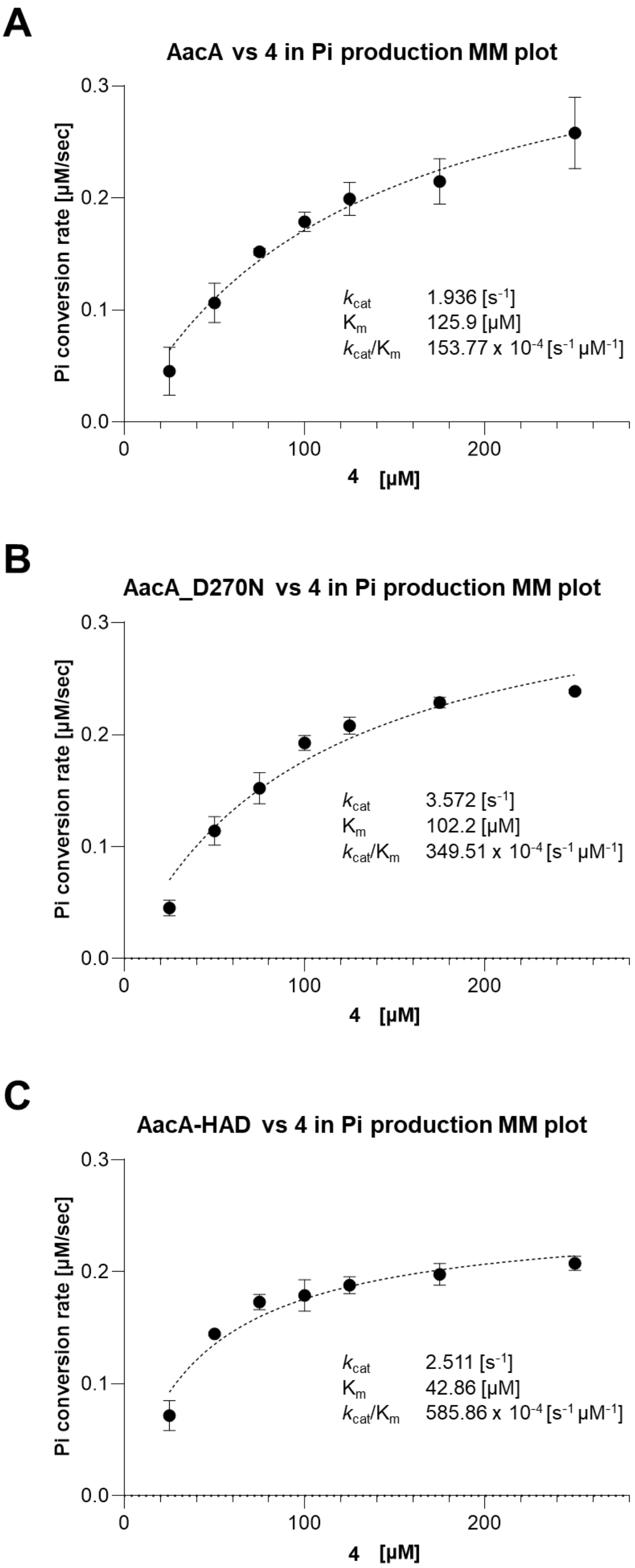

Figure S23. Michaelis-Menten plots of (A) AacA, (B) AacA_D270N, and (C) AacA-HAD with substrate **4**.

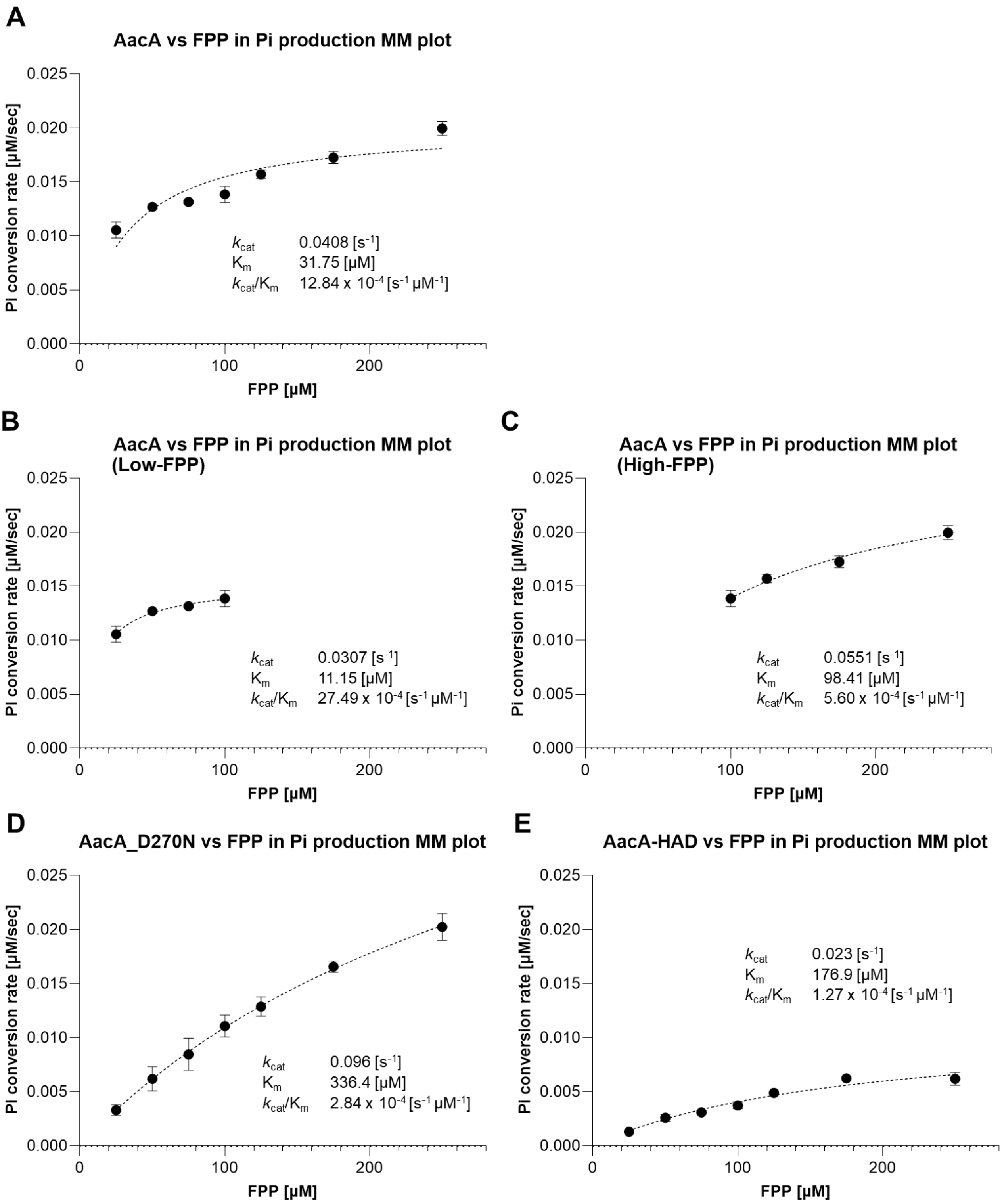

Figure S24. Michaelis-Menten plots of (A–C) AacA, and (D) AacA_D270N, and (E) AacA-HAD with FPP. (A) AacA with full range of FPP concentration (25–250 μM); (B) AacA with low-FPP regime (25–75 μM); (C) AacA with high-FPP regime (100–250 μM). The concentration of AacA was 0.5 μM for the kinetic assays.

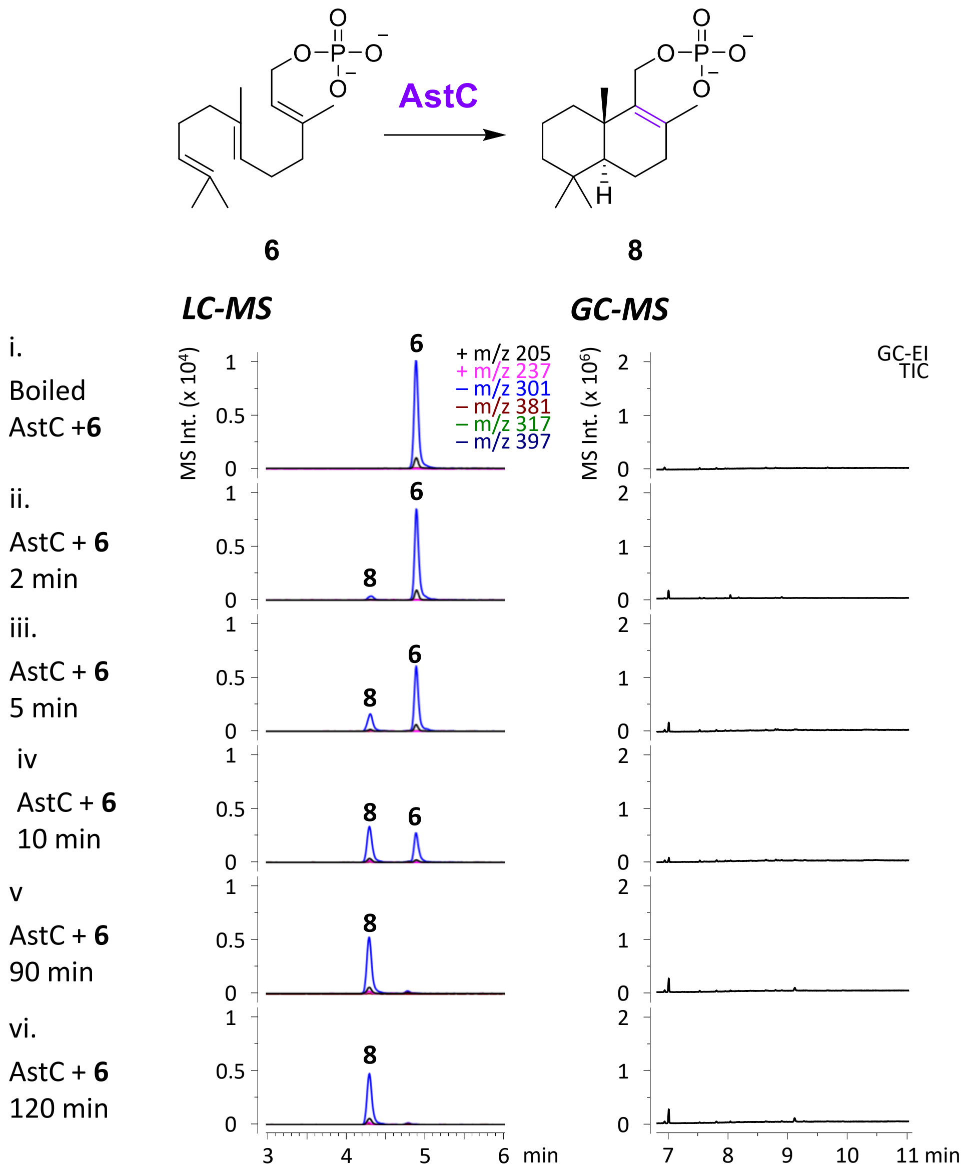

Figure S25. LC–MS and GC–MS analysis of *in vitro* assays of **6** with AstC over a 2–120 min time course. Reaction mixtures contained 1 µM AstC and 100 µM **6** and were incubated at ambient temperature. Reactions were quenched with acetonitrile and analyzed by LC–ESI–MS (left panels). The same reactions were also directly analyzed by SPME–GC–EI–MS (right panels).

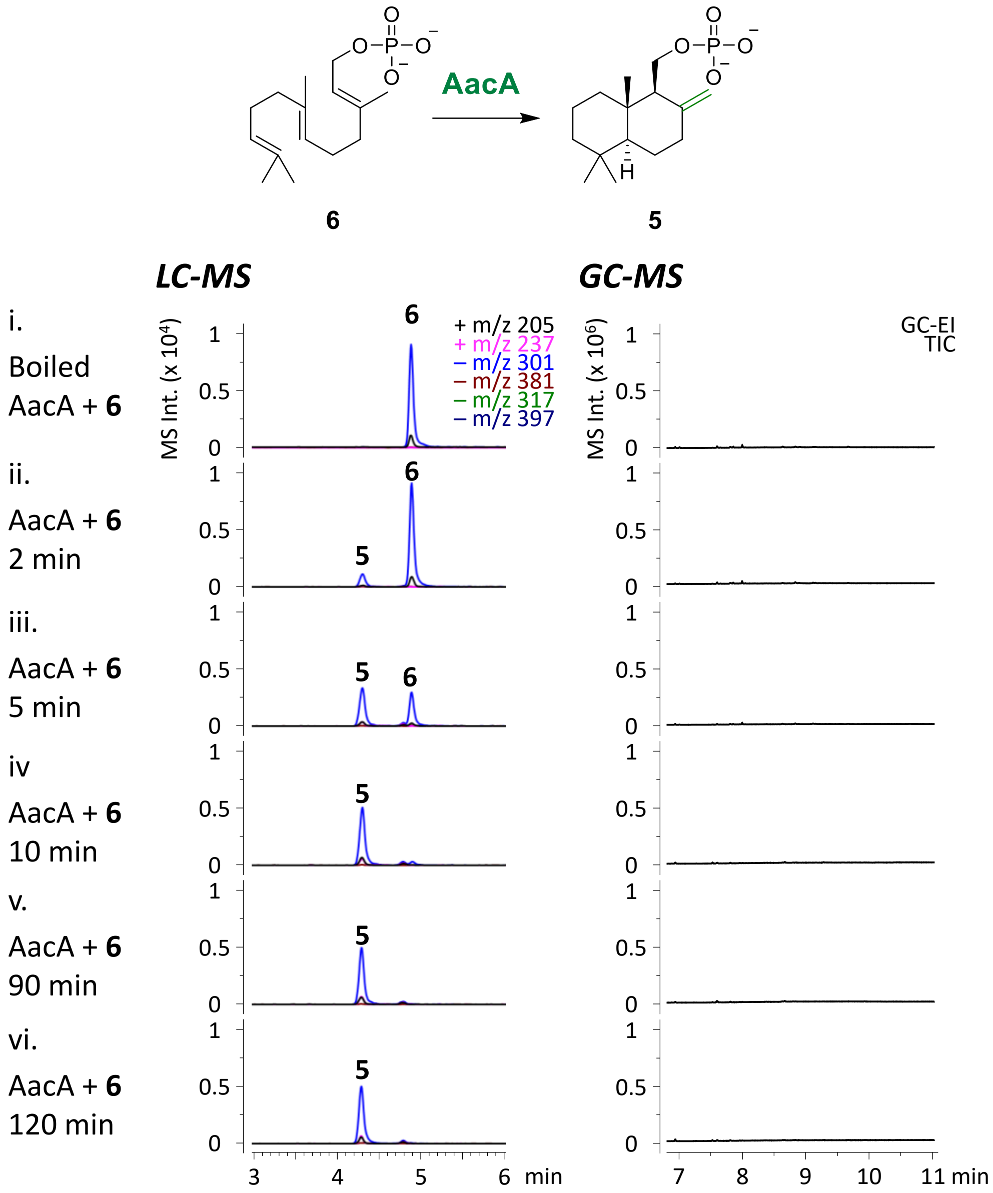

Figure S26. LC–MS and GC–MS analysis of *in vitro* assays of **6** with AacA over a 2–120 min time course. Reaction mixtures contained 1 μM AacA and 100 μM **6** and were incubated at ambient temperature. Reactions were quenched with acetonitrile and analyzed by LC–ESI–MS (left panels). The same reactions were also directly analyzed by SPME–GC–EI–MS (right panels).

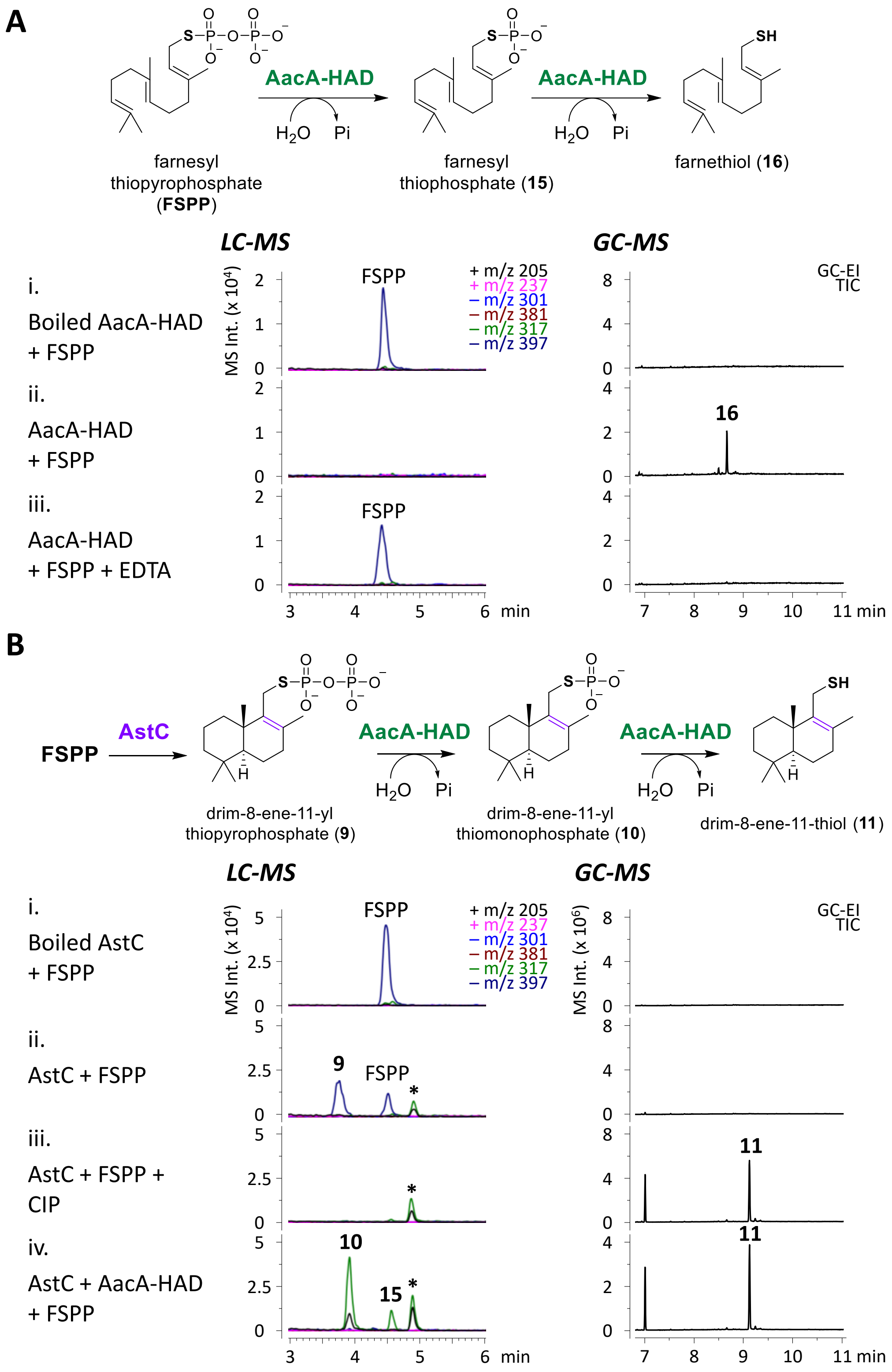

Figure S27. LC–MS and GC–MS analysis of *in vitro* assays of FSPP with (A) AacA-HAD, or (B) AstC with or without AacA-HAD. Reaction mixtures contained 1 μM enzyme and 100 μM FSPP in 100 mM Tris–HCl (pH 7.5) supplemented with either 0.5 mM $MgCl_2$ or 2 mM EDTA, and were incubated at ambient temperature. Reactions were quenched with acetonitrile and analyzed by LC–ESI–MS (left panels). The same reactions were also directly analyzed by SPME–GC–EI–MS (right panels). The peak marked with an asterisk is hypothesized to be a shunt product.

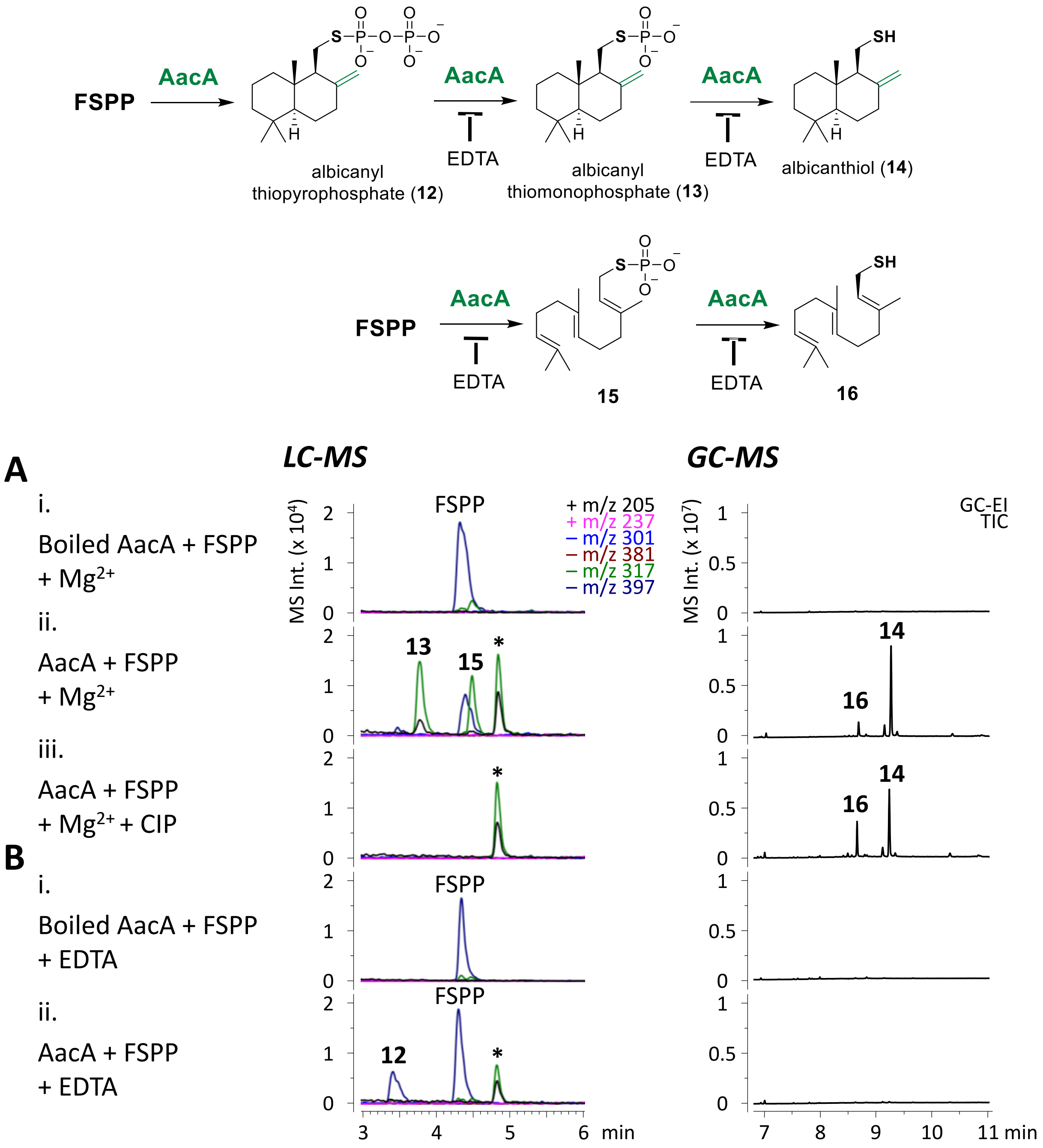

Figure S28. LC–MS and GC–MS analysis of *in vitro* assays of FSPP with (A) AacA, or (B) AacA and EDTA. Reaction mixtures contained 1 µM AacA and 100 µM FSPP in 100 mM Tris-HCl (pH 7.5) supplemented with either 0.5 mM $MgCl_2$ or 2 mM EDTA, and were incubated at ambient temperature. Reactions were quenched with acetonitrile and analyzed by LC–ESI–MS (left panels). The same reactions were also directly analyzed by SPME–GC–EI–MS (right panels). The peak marked with an asterisk is hypothesized to be a shunt product.

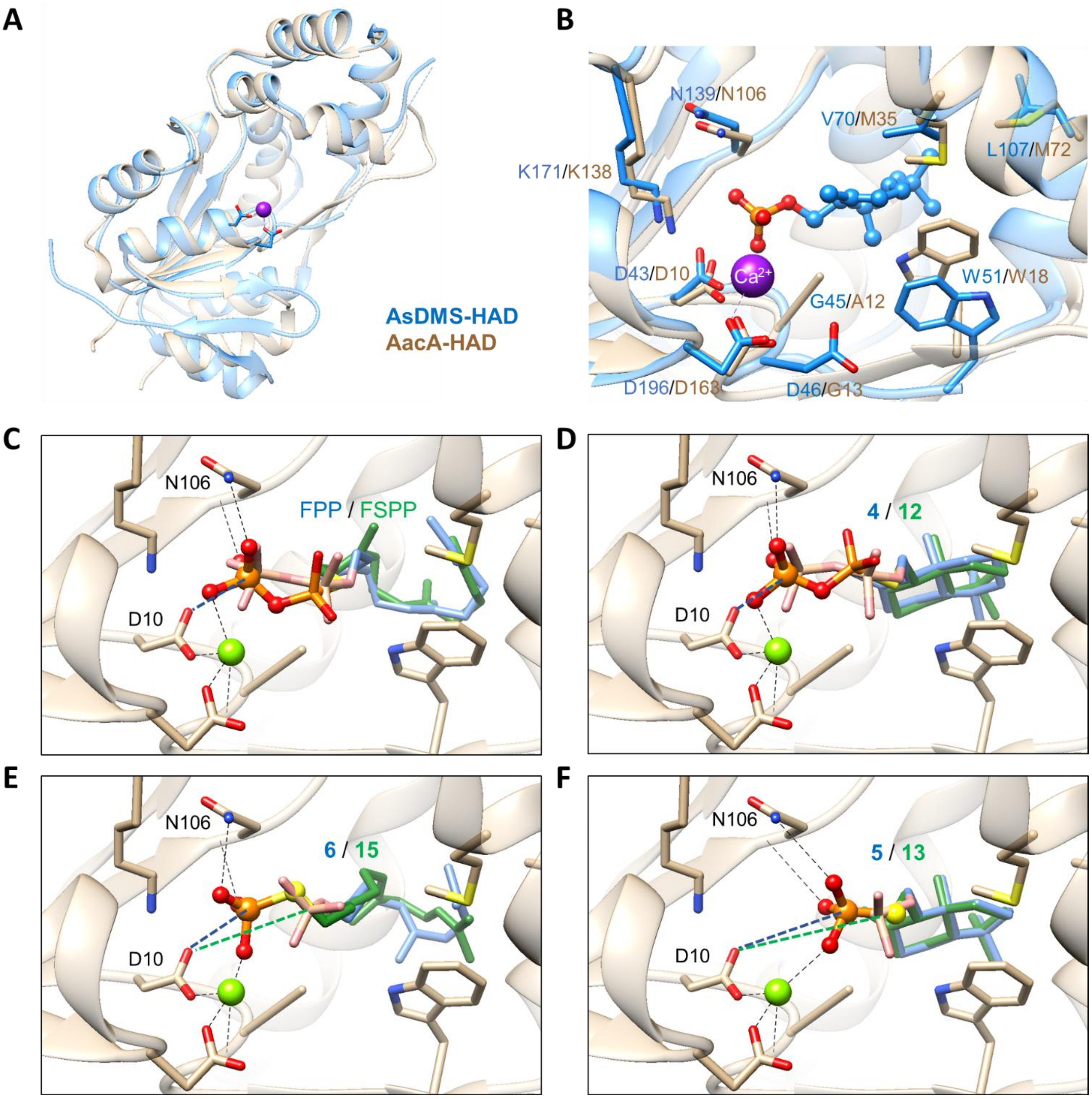

Figure S29. Structural comparison and docking analysis rationalizing preferential α-phosphate hydrolysis of thiophosphate substrates by AacA. **(A)** Structural superposition of the HAD-like domain of the AlphaFold 3[19]-predicted AacA model (tan) with the crystal structure of AsDMS in complex with drimenyl monophosphate (blue; PDB 9M7E), showing conservation of the overall HAD fold. **(B)** Close-up view of the HAD active site. Conserved AacA residues D10, N106, and D163 align with AsDMS D43, N139, and D196, respectively, and define the phosphate/metal-binding center (Ca^{2+} from AsDMS shown as a purple sphere). In contrast, residues shaping the hydrophobic prenyl-binding pocket differ, including AacA A12, G13, W18, M35, and M72 (corresponding to AsDMS G45, D46, W51, V70, and L107), indicating a remodeled substrate-binding cavity. **(C–F)** Representative docking poses of phosphate- and thiophosphate-containing ligands in the AacA HAD active site. FPP and FSPP **(C)**, as well as albicanoyl pyrophosphate **4** and albicanoyl thiopyrophosphate **12 (D)**, adopt similar pre-hydrolytic binding modes, in which the α-phosphate engages N106 and the Mg^{2+} cofactor (~3 Å) and places the phosphorus atom within ~6 Å of catalytic D10 (blue dashed line), consistent with a geometry competent for nucleophilic attack. In contrast, the corresponding monophosphate ligands show divergent binding modes: farnesyl

monophosphate **6** and albicanoyl monophosphate **5** dock distal to the catalytic center (>6 Å, green dashed line), whereas farnesyl thiomonophosphate **15** and albicanoyl thiomonophosphate **13** remain positioned closer to the catalytic core **(E, F)**. These results suggest that sulfur substitution alters headgroup geometry and active-site interactions in a manner that preserves productive binding of thiophosphate-derived intermediates, thereby providing a structural rationale for the observed stepwise β- and α-phosphate hydrolysis of thiophosphate substrates by AacA.

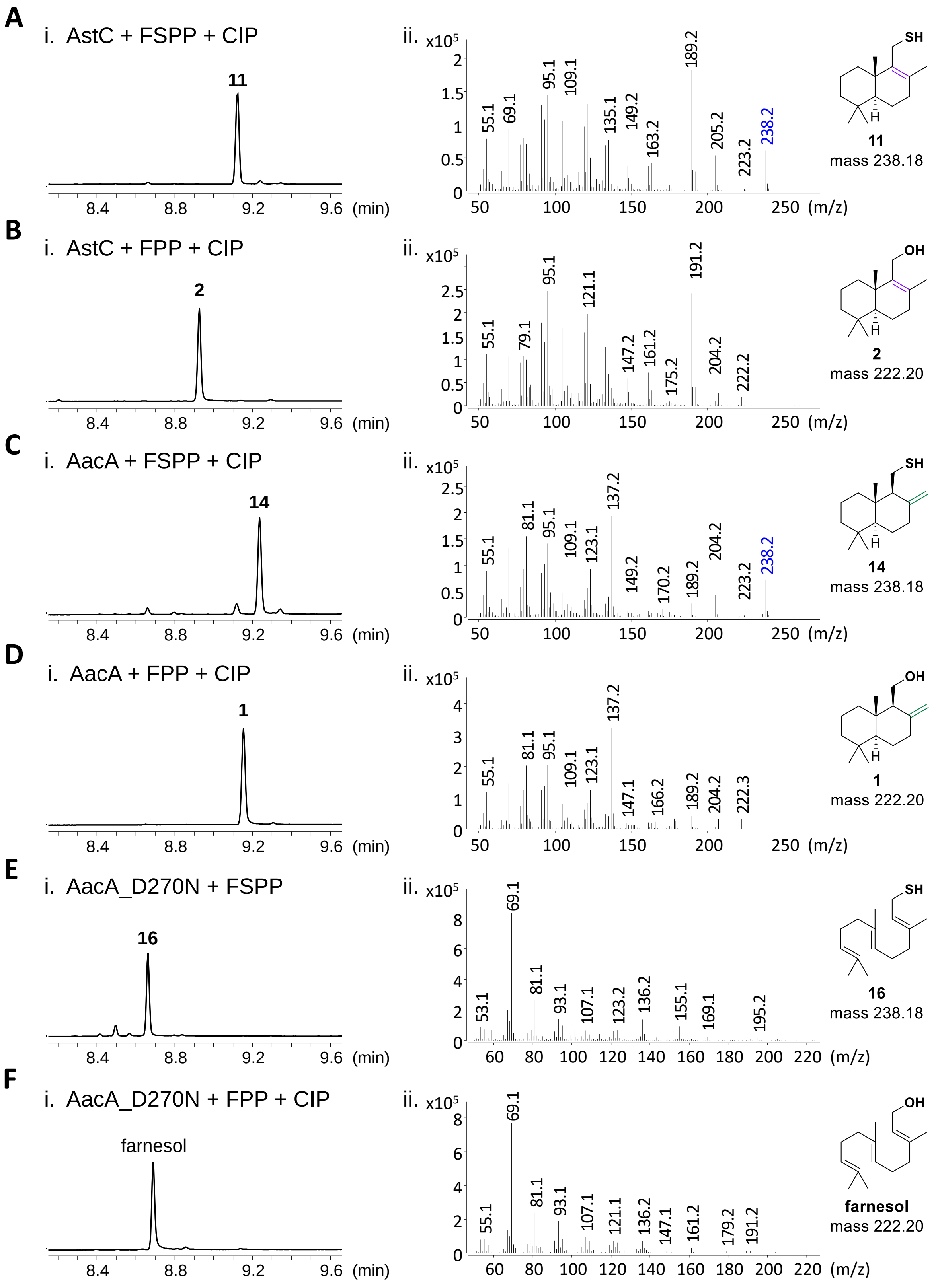

Figure S30. GC–MS analysis and the EI mass spectra of FSPP or FPP derived compounds, (A) **11**, (B) **2**, (C) **14**, (D) **1**, (E) **16**, and (F) farnesol. Reaction mixtures contained 5 μM enzyme and 100 μM substrate incubated at ambient temperature for 30 min and analyzed by SPME–GC–EIMS.

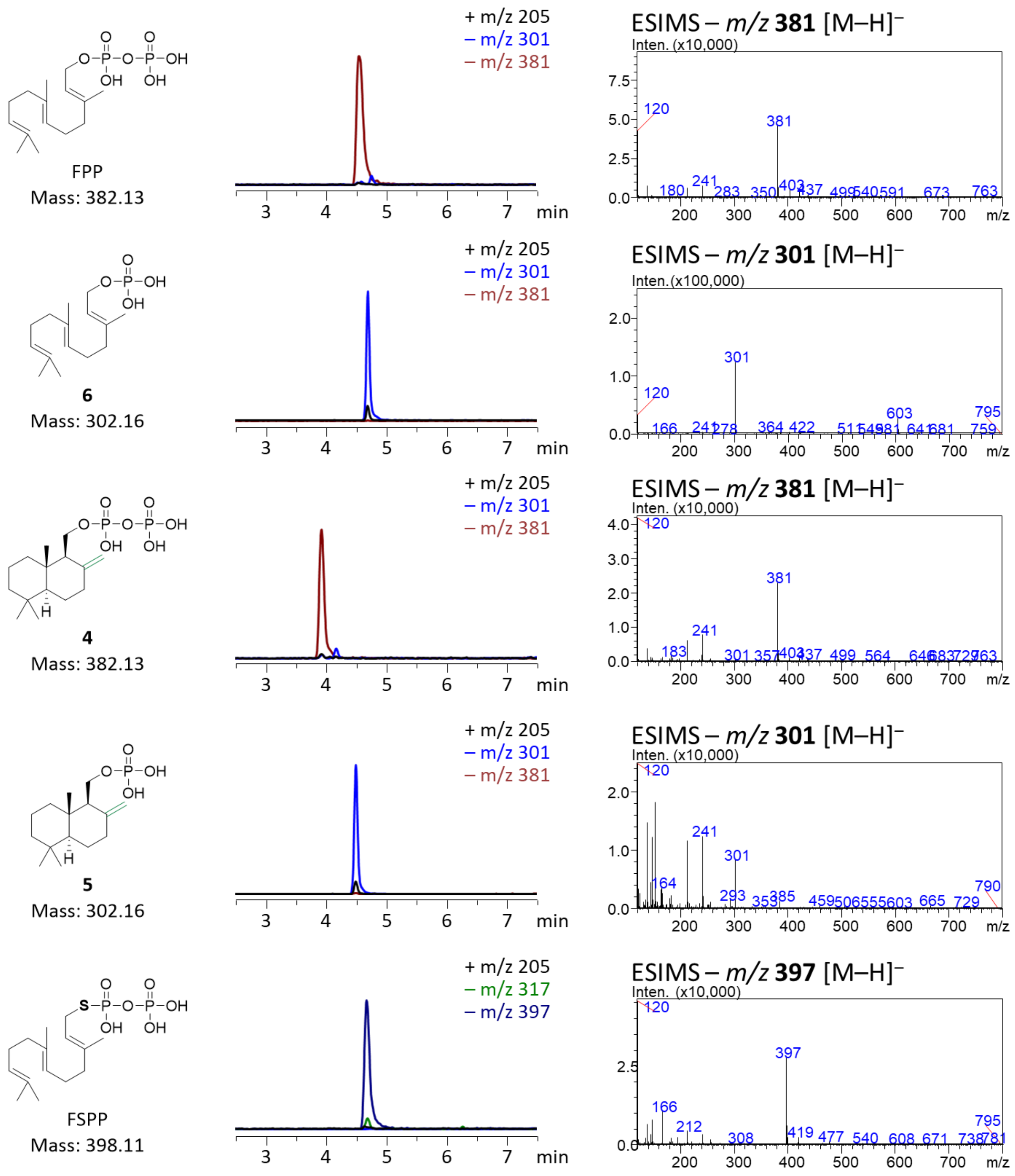

Figure S31. LC–MS analysis and the mass spectra of FPP, **6**, **4**, **5**, and FSPP. MS spectra are at negative mode.

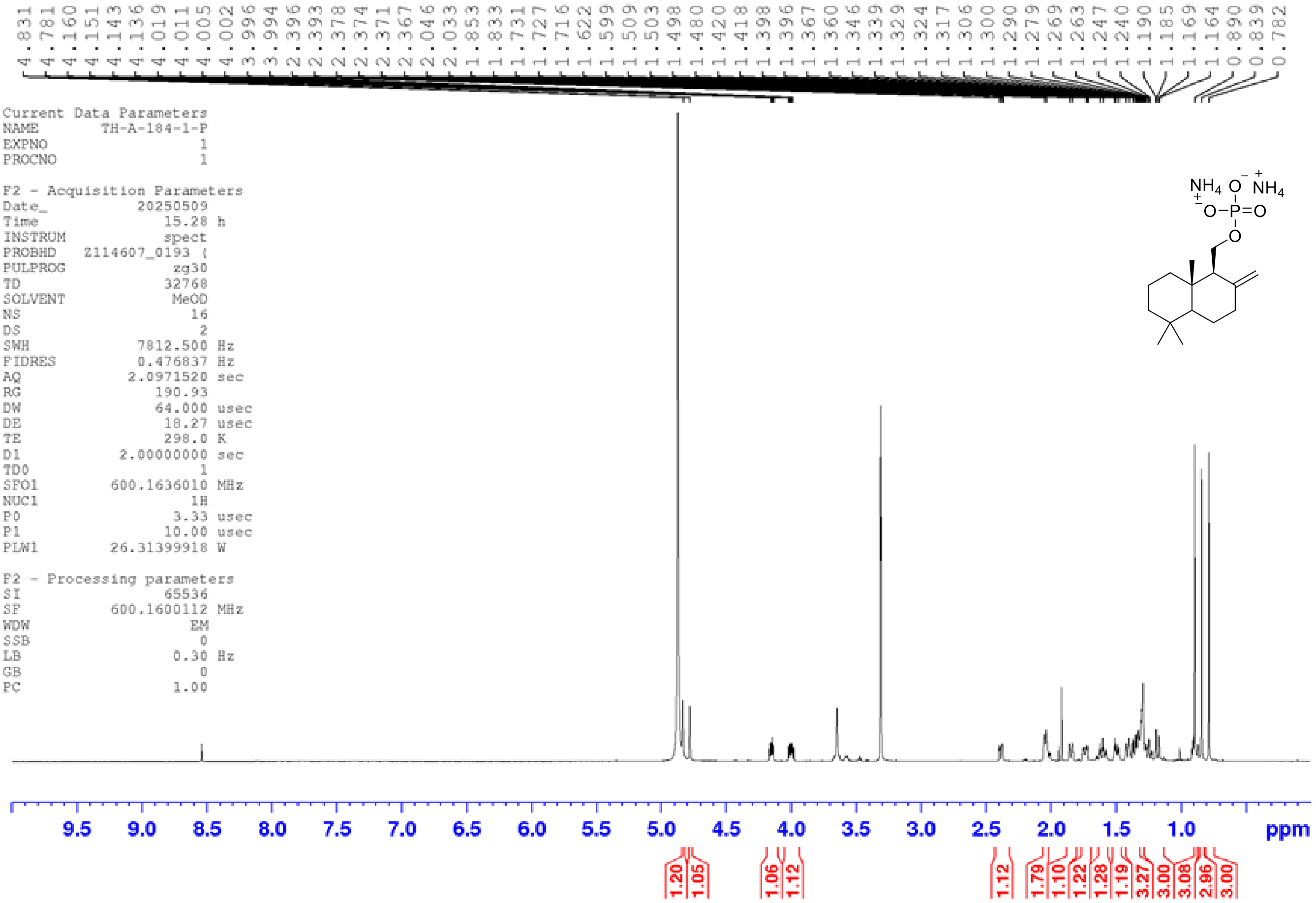

Figure S32. ^1H NMR spectrum of **4** (CD_3OD, 600 MHz).

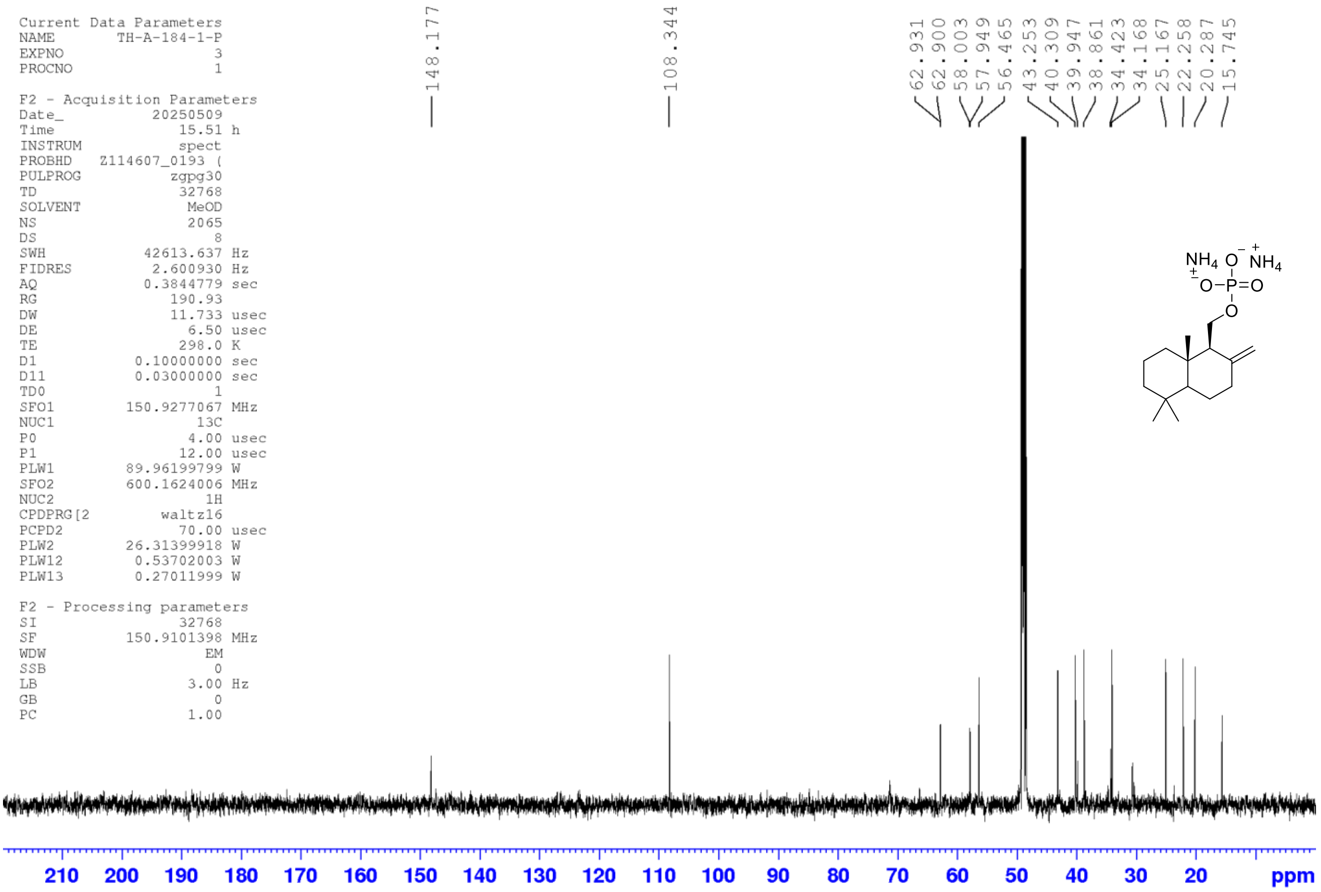

Figure S33. ^{13}C NMR spectrum of **4** (CD_3OD, 150 MHz).

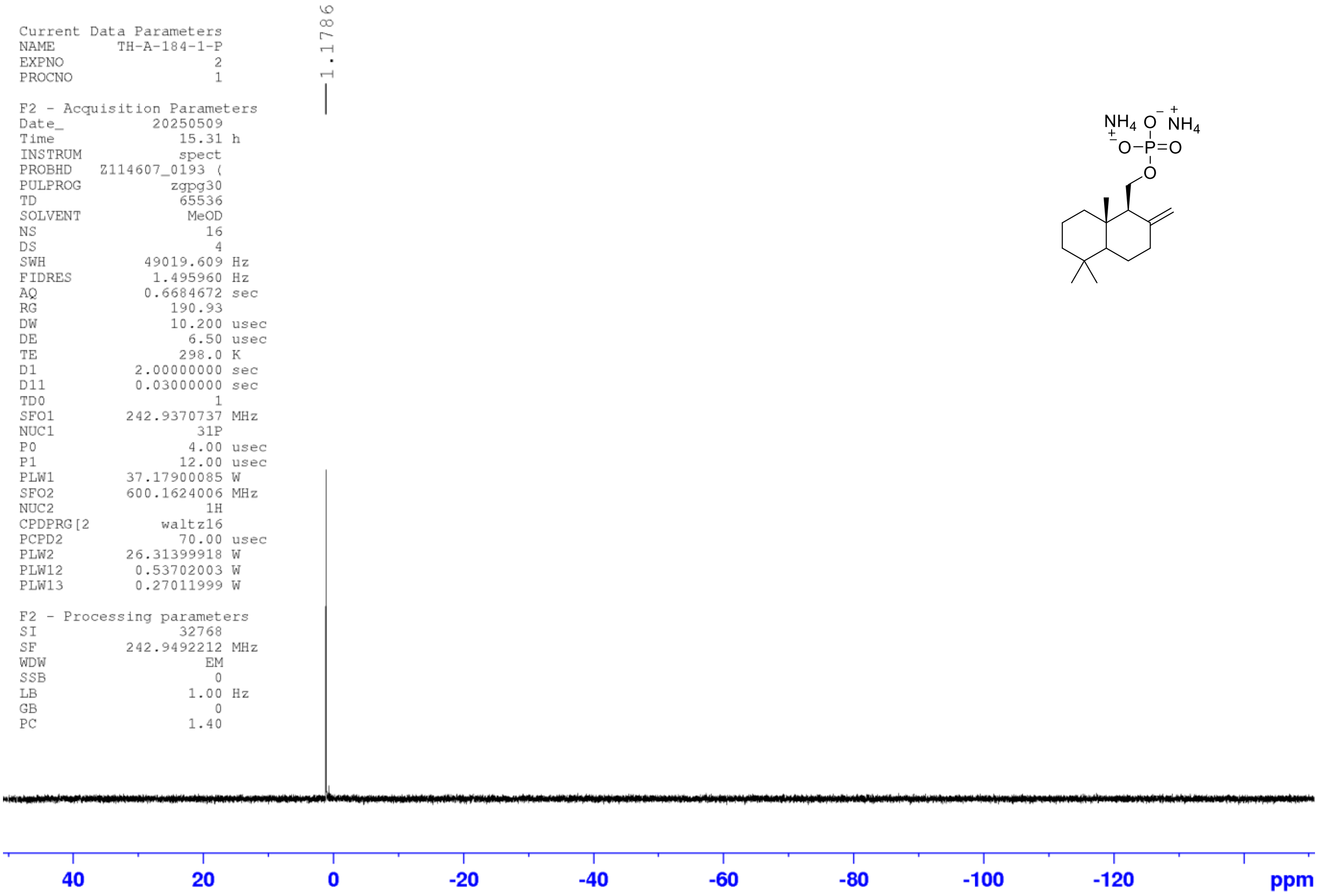

Figure S34. ^{31}P NMR spectrum of **4** (CD_3OD, 243 MHz).

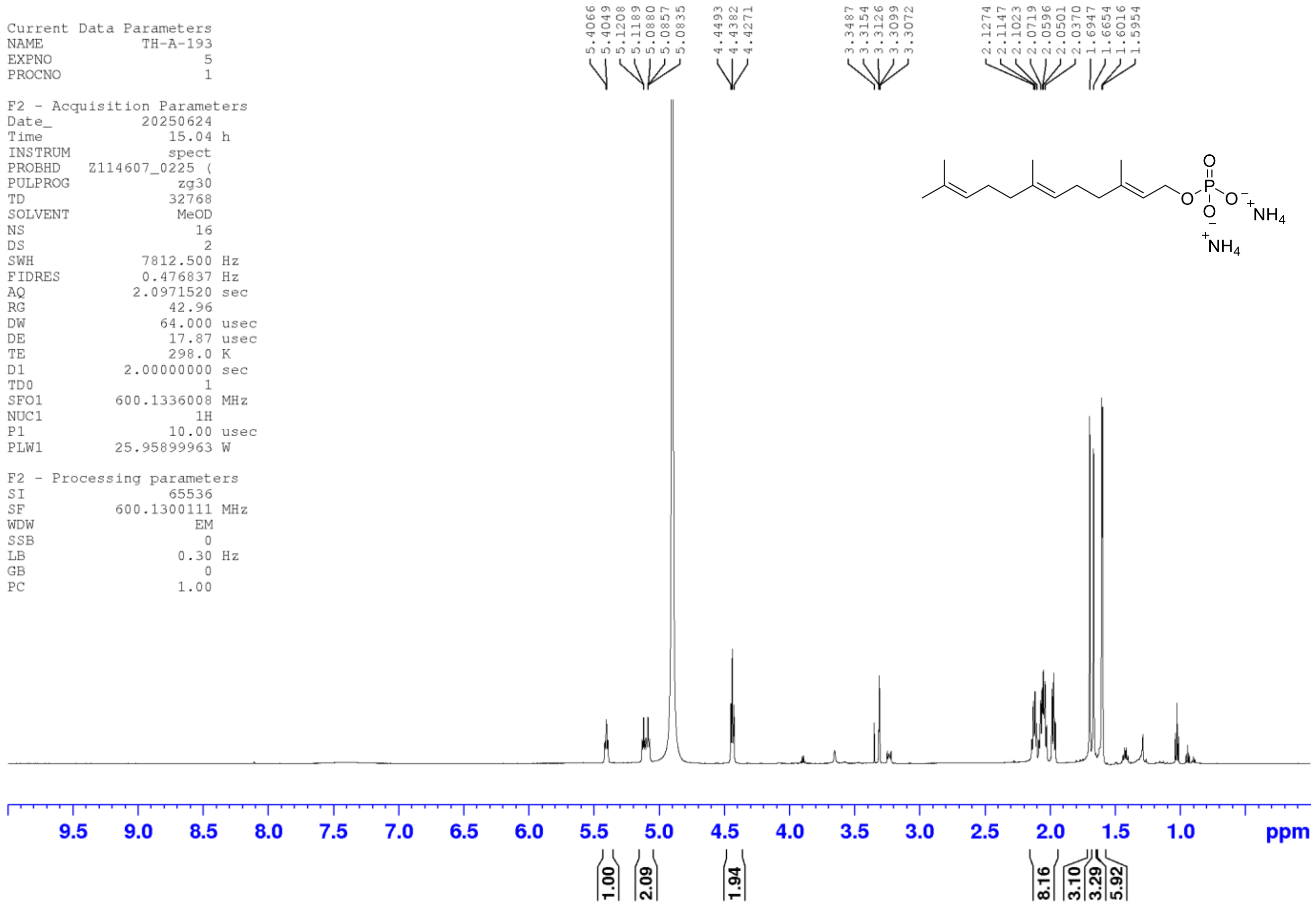

Figure S35. ^{1}H NMR spectrum of **6** (CD_3OD, 600 MHz).

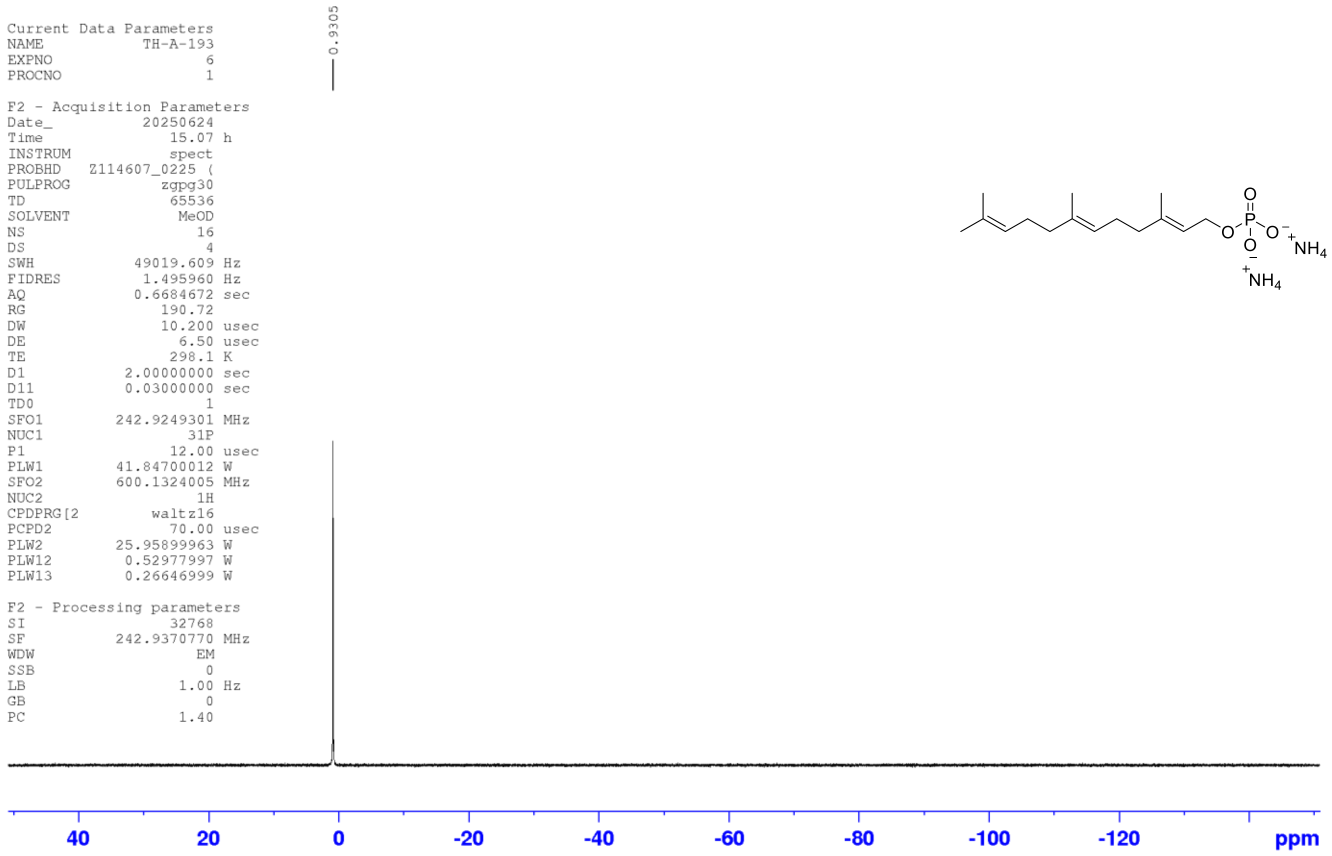

Figure S36. ^{31}P NMR spectrum of **6** (CD_3OD, 243 MHz).

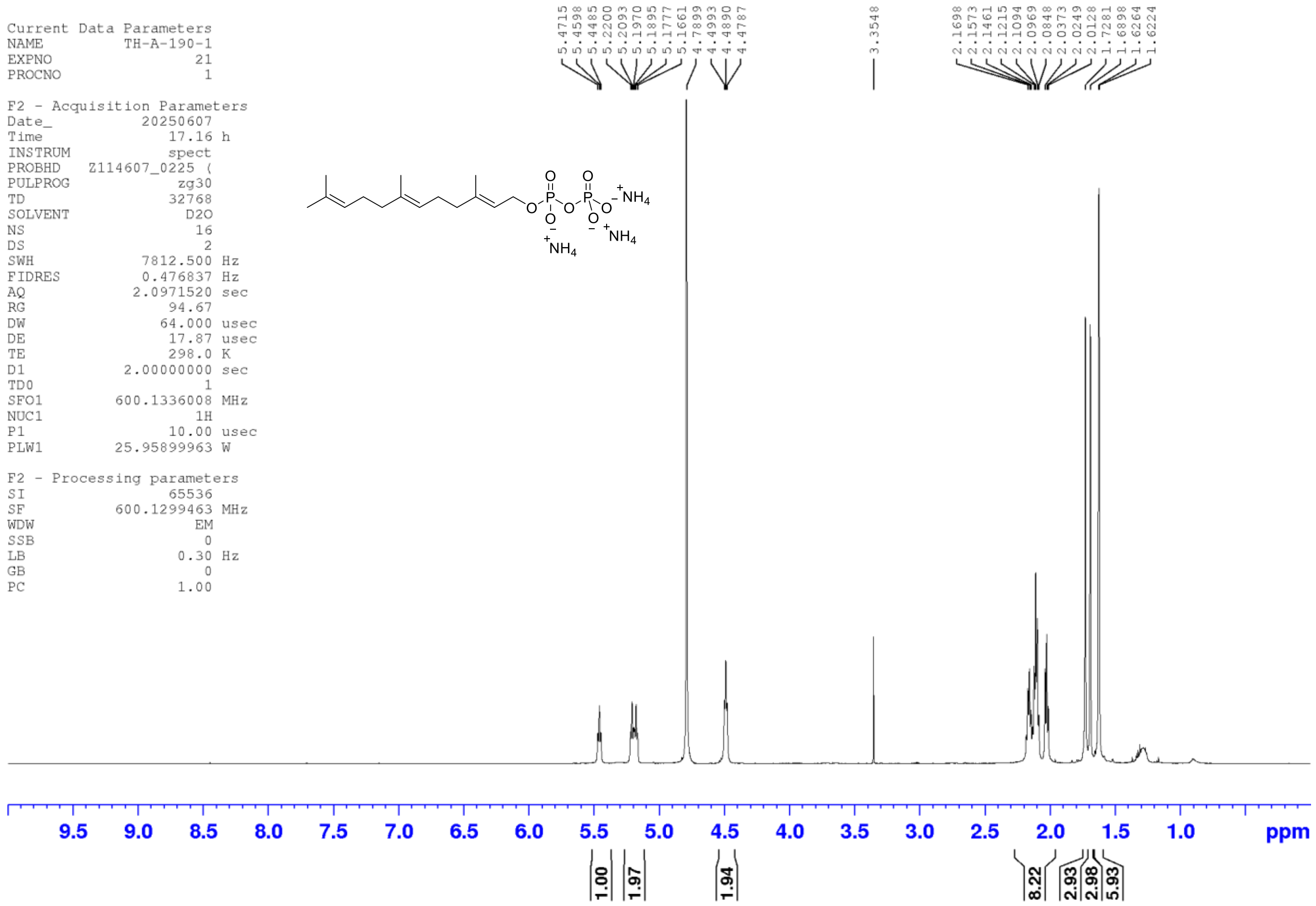

Figure S37. ^{1}H NMR spectrum of FPP (D_2O, 600 MHz).

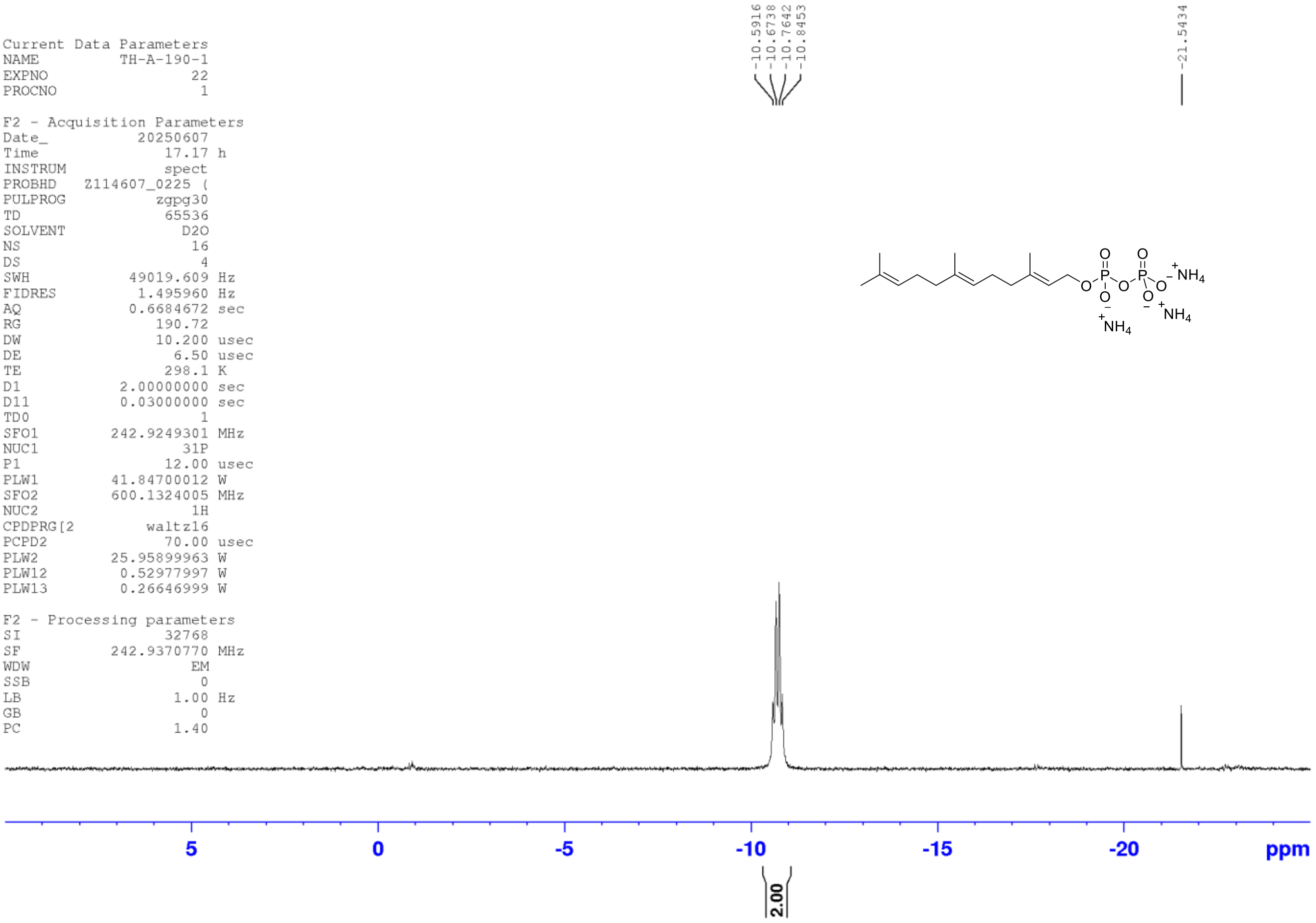

Figure S38. ^{31}P NMR spectrum of FPP (D_2O, 243 MHz).

References

(1) Chen, T.; Chen, C.; Lee, C.; Huang, R.; Chen, K.; Lu, Y.; Liang, S.; Pham, M.; Rao, Y.; Wu, S.; Chein, R.; Lin, H., The biosynthetic gene cluster of mushroom-derived antrocin encodes two dual-functional haloacid dehalogenase-like terpene cyclases. *Angew. Chem. Int. Ed.* **2023,** *62*, e202215566.

(2) Altschul, S. F.; Madden, T. L.; Schaffer, A. A.; Zhang, J. H.; Zhang, Z.; Miller, W.; Lipman, D. J., Gapped BLAST and PSI-BLAST: a new generation of protein database search programs. *Nucleic Acids Res.* **1997,** *25*, 3389-3402.

(3) Enright, A. J.; Van Dongen, S.; Ouzounis, C. A., An efficient algorithm for large-scale detection of protein families. *Nucleic Acids Res.* **2002,** *30*, 1575-1584.

(4) Huang, Y.; Niu, B. F.; Gao, Y.; Fu, L. M.; Li, W. Z., CD-HIT Suite: a web server for clustering and comparing biological sequences. *Bioinformatics* **2010,** *26*, 680-682.

(5) Huerta-Cepas, J.; Serra, F.; Bork, P., ETE 3: Reconstruction, analysis, and visualization of phylogenomic data. *Mol. Biol. Evol.* **2016,** *33*, 1635-1638.

(6) Katoh, K.; Misawa, K.; Kuma, K.; Miyata, T., MAFFT: a novel method for rapid multiple sequence alignment based on fast Fourier transform. *Nucleic Acids Res.* **2002,** *30*, 3059-3066.

(7) Price, M. N.; Dehal, P. S.; Arkin, A. P., FastTree 2-approximately maximum-likelihood trees for large alignments. *Plos One* **2010,** *5*, e9490.

(8) Mai, U.; Mirarab, S., TreeShrink: fast and accurate detection of outlier long branches in collections of phylogenetic trees. *Bmc Genomics* **2018,** *19*, 272.

(9) Yu, G. C.; Smith, D. K.; Zhu, H. C.; Guan, Y.; Lam, T. T. Y., GGTREE: an R package for visualization and annotation of phylogenetic trees with their covariates and other associated data. *Methods Ecol. Evol.* **2017,** *8*, 28-36.

(10) Team, R. C., A language and environment for statistical computing. R Foundation for Statistical Computing. ISBN 3-900051-07-0, **2013**. URL http://www.R-project.org.

(11) Menardo, F.; Loiseau, C.; Brites, D.; Coscolla, M.; Gygli, S. M.; Rutaihwa, L. K.; Trauner, A.; Beisel, C.; Borrell, S.; Gagneux, S., Treemmer: a tool to reduce large phylogenetic datasets with minimal loss of diversity. *Bmc Bioinformatics* **2018,** *19*,164.

(12) Huang, Y.; Hoefgen, S.; Valiante, V., Biosynthesis of Fungal Drimane-Type Sesquiterpene Esters. *Angew. Chem. Int. Ed.* **2021,** *60*, 23763-23770.

(13) Otwinowski, Z.; Minor, W., Processing of X-ray diffraction data collected in oscillation mode. *Methods Enzymol.* **1997,** *276*, 307-26.

(14) McCoy, A. J.; Grosse-Kunstleve, R. W.; Adams, P. D.; Winn, M. D.; Storoni, L. C.; Read, R. J., Phaser crystallographic software. *J. Appl. Crystallogr.* **2007,** *40*, 658-674.

(15) Abramson, J.; Adler, J.; Dunger, J.; Evans, R.; Green, T.; Pritzel, A.; Ronneberger, O.; Willmore, L.; Ballard, A. J.; Bambrick, J.; Bodenstein, S. W.; Evans, D. A.; Hung, C. C.; O'Neill, M.; Reiman, D.; Tunyasuvunakool, K.; Wu, Z.; Zemgulyte, A.; Arvaniti, E.; Beattie, C.; Bertolli, O.; Bridgland, A.; Cherepanov, A.; Congreve, M.; Cowen-Rivers, A. I.; Cowie, A.; Figurnov, M.; Fuchs, F. B.; Gladman, H.; Jain, R.; Khan, Y. A.; Low, C. M. R.; Perlin, K.; Potapenko, A.; Savy, P.; Singh, S.; Stecula, A.; Thillaisundaram, A.; Tong, C.; Yakneen, S.; Zhong, E. D.; Zielinski, M.; Zidek, A.; Bapst, V.; Kohli, P.; Jaderberg, M.; Hassabis, D.; Jumper, J. M., Accurate structure prediction of biomolecular interactions with AlphaFold 3. *Nature* **2024,** *630*, 493-500.

(16) Emsley, P.; Cowtan, K., Coot: model-building tools for molecular graphics. *Acta Crystallogr. D Biol. Crystallogr.* **2004,** *60*, 2126-32.

(17) Adams, P. D.; Afonine, P. V.; Bunkoczi, G.; Chen, V. B.; Davis, I. W.; Echols, N.; Headd, J. J.; Hung, L. W.; Kapral, G. J.; Grosse-Kunstleve, R. W.; McCoy, A. J.; Moriarty, N. W.; Oeffner, R.; Read, R. J.; Richardson, D. C.; Richardson, J. S.; Terwilliger, T. C.; Zwart, P. H., PHENIX: a comprehensive Python-based system for macromolecular structure solution. *Acta Crystallogr. D Biol. Crystallogr.* **2010,** *66*, 213-21.

(18) Williams, C. J.; Headd, J. J.; Moriarty, N. W.; Prisant, M. G.; Videau, L. L.; Deis, L. N.; Verma, V.; Keedy, D. A.; Hintze, B. J.; Chen, V. B.; Jain, S.; Lewis, S. M.; Arendall, W. B., 3rd; Snoeyink, J.; Adams, P. D.; Lovell,

S. C.; Richardson, J. S.; Richardson, D. C., MolProbity: More and better reference data for improved all-atom structure validation. *Protein Sci.* **2018,** *27* (1), 293-315.

(19) (a) Kuznetsova, E.; Proudfoot, M.; Gonzalez, C. F.; Brown, G.; Omelchenko, M. V.; Borozan, I.; Carmel, L.; Wolf, Y. I.; Mori, H.; Savchenko, A. V.; Arrowsmith, C. H.; Koonin, E. V.; Edwards, A. M.; Yakunin, A. F., Genome-wide analysis of substrate specificities of the *Escherichia coli* haloacid dehalogenase-like phosphatase family. *J. Biol. Chem.* **2006,** *281*, 36149-36161; (b) Tremblay, L. W.; Dunaway-Mariano, D.; Allen, K. N., Structure and activity analyses of *Escherichia coli* K-12 NagD provide insight into the evolution of biochemical function in the haloalkanoic acid dehalogenase superfamily. *Biochemistry* **2006,** *45*, 1183-1193.

(20) Rye, H. S.; Burston, S. G.; Fenton, W. A.; Beechem, J. M.; Xu, Z.; Sigler, P. B.; Horwich, A. L., Distinct actions of cis and trans ATP within the double ring of the chaperonin GroEL. *Nature* **1997,** *388*, 792-798.

(21) Rohrwild, M.; Coux, O.; Huang, H. C.; Moerschell, R. P.; Yoo, S. J.; Seol, J. H.; Chung, C. H.; Goldberg, A. L., HslV-HslU: A novel ATP-dependent protease complex in *Escherichia coli* related to the eukaryotic proteasome. *Proc. Natl. Acad. Sci. U.S.A.* **1996,** *93*, 5808-5813.
